# Supplementary material for: Synthesis and Molecular Docking Studies of Alkoxy- and Imidazole-Substituted Xanthones as α-Amylase and α-Glucosidase Inhibitors
Source: Molecules. 2023 May 18;28(10):4180. doi: 10.3390/molecules28104180 (PMC10222686; doi:10.3390/molecules28104180)
Supplement: Supplementary file 1 [file molecules-28-04180-s001.zip › molecules-2374915-supplementary.pdf]

Supplementary Material

Synthesis and Molecular Docking Studies of Alkoxy- and Imidazole-Substituted Xanthones as α-Amylase and α-Glucosidase Inhibitors

Dolores G. Aguila-Muñoz [1], Gabriel Vázquez-Lira [1], Erika Sarmiento-Tlale [1], María C. Cruz-López [1], Fabiola E. Jiménez-Montejo [1], Víctor E. López y López [1], Carlos H. Escalante [2], Dulce Andrade-Pavón [3,4], Omar Gómez-García [2], Joaquín Tamariz [2] and Aarón Mendieta-Moctezuma [1,*]

[1] Centro de Investigación en Biotecnología Aplicada, Instituto Politécnico Nacional, Carretera Estatal Santa Inés Tecuexcomax-Tepetitla, Km 1.5, Tepetitla de Lardizábal, Tlaxcala 90700, Mexico; lupaita@gmail.com (D.G.A.-M.); eagle_vl@hotmail.com (G.V.-L.); erika16.st@gmail.com (E.S.-T.); ccruzl@ipn.mx (M.C.C.-L.); fejimenezm@ipn.mx (F.E.J.-M.); vlopezyl@ipn.mx (V.E.L.y.L.)

[2] Departamento de Química Orgánica, Escuela Nacional de Ciencias Biológicas, Instituto Politécnico Nacional, Prol. Carpio y Plan de Ayala S/N, Mexico City 11340, Mexico; escalantecah@gmail.com (C.H.E.); gogamanj@hotmail.com (O.G.-G.); jtamariz@yahoo.com.mx (J.T.)

[3] Departamento de Fisiología, Escuela Nacional de Ciencias Biológicas, Instituto Politécnico Nacional, Av. Wilfrido Massieu S/N, Mexico City 11340, México; andrade_eclud88@hotmail.com

[4] Departamento de Microbiología, Escuela Nacional de Ciencias Biológicas, Instituto Politécnico Nacional, Prol. Carpio y Plan de Ayala S/N, Mexico City 11340, Mexico

* Correspondence: amendietam@ipn.mx or epsilon_1409@yahoo.com.mx

Copies of ^{1}H and ^{13}C NMR, and HRMS spectra for compounds **3-12**

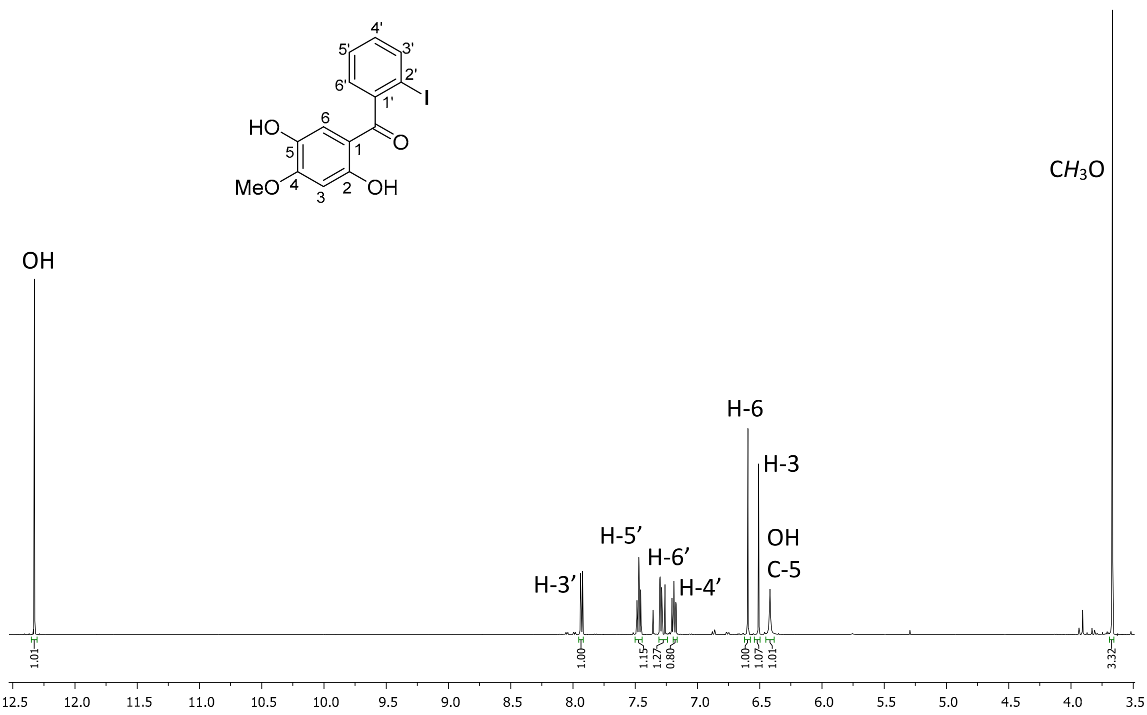

Figure S1. ^{1}H NMR (500 MHz, CDCl$_3$) spectrum of **3a**.

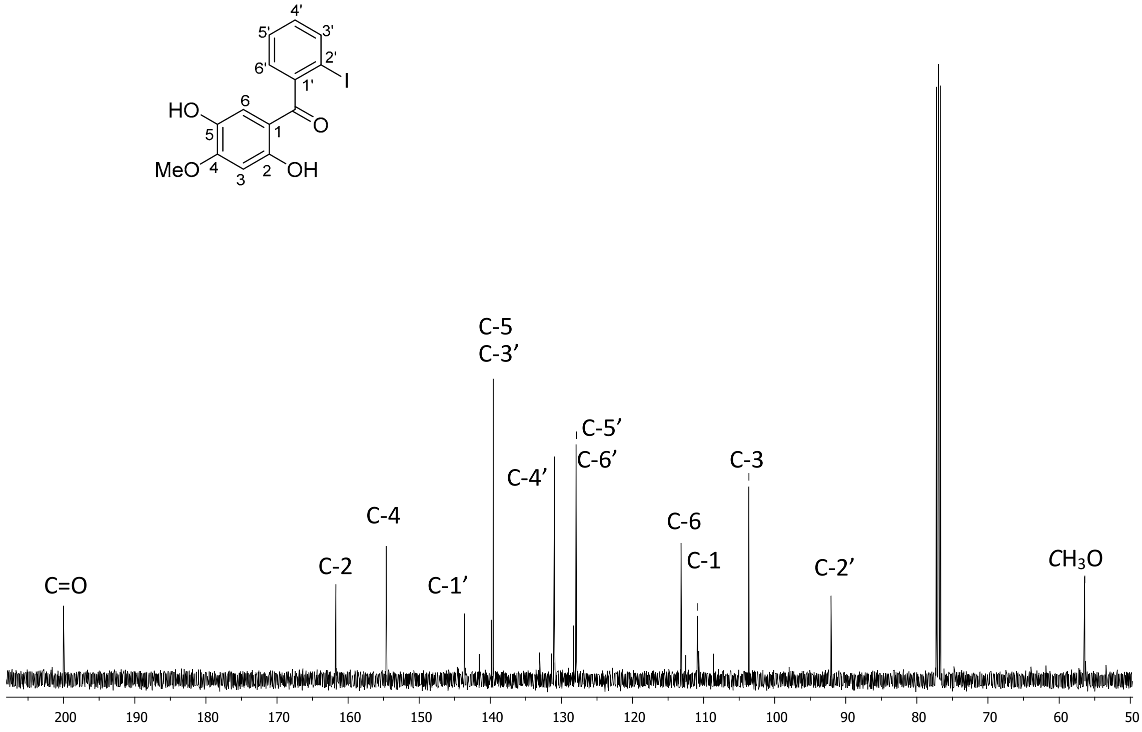

Figure S2. ^{13}C NMR (125 MHz, CDCl$_3$) spectrum of **3a**.

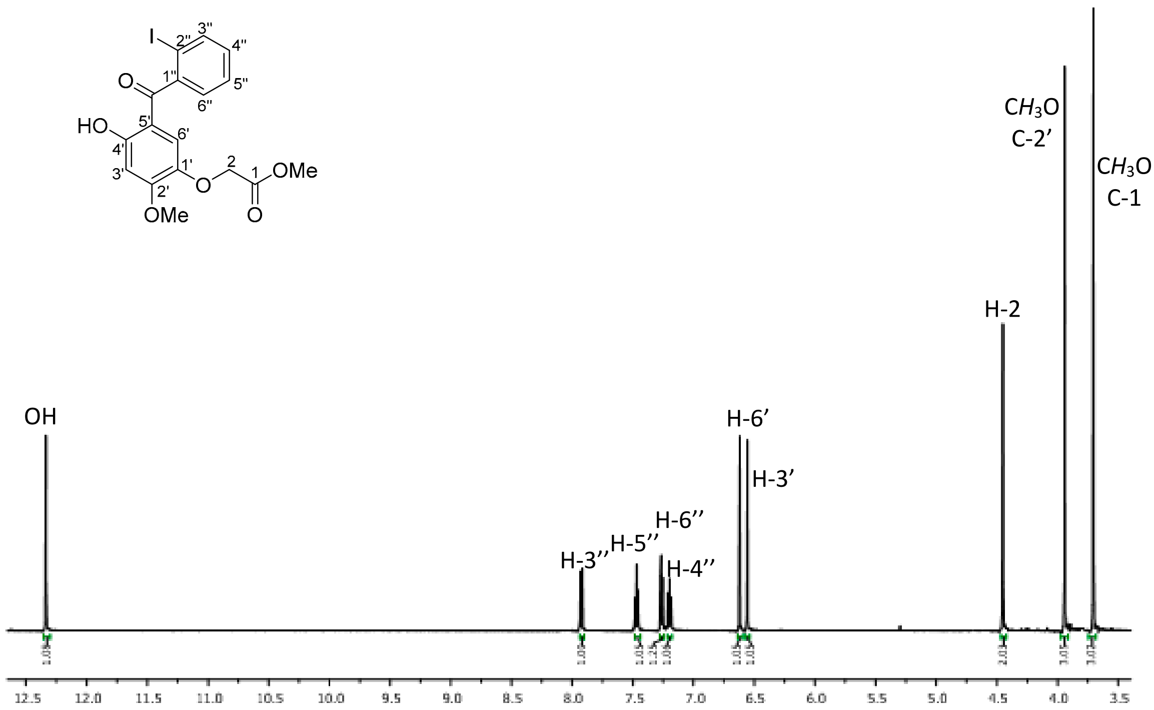

Figure S3. ^{1}H NMR (500 MHz, $CDCl_3$) spectrum of **3b**.

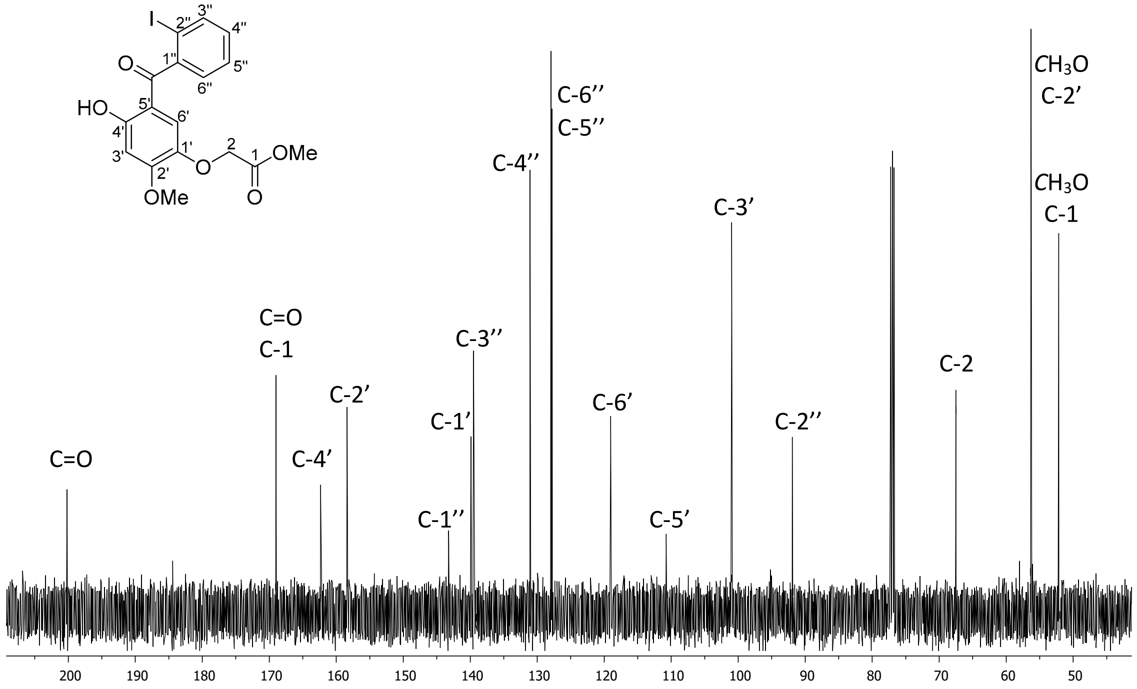

Figure S4. ^{13}C NMR (125 MHz, $CDCl_3$) spectrum of **3b**.

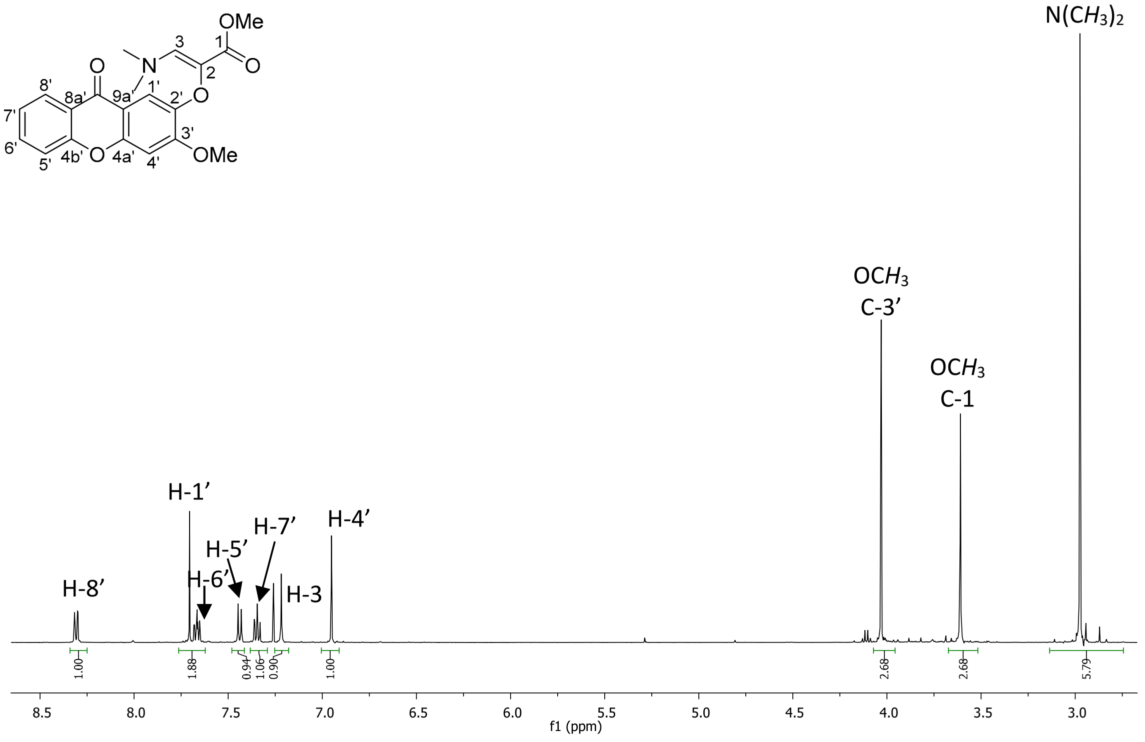

Figure S5. ^{1}H NMR (500 MHz, $CDCl_3$) spectrum of **5**.

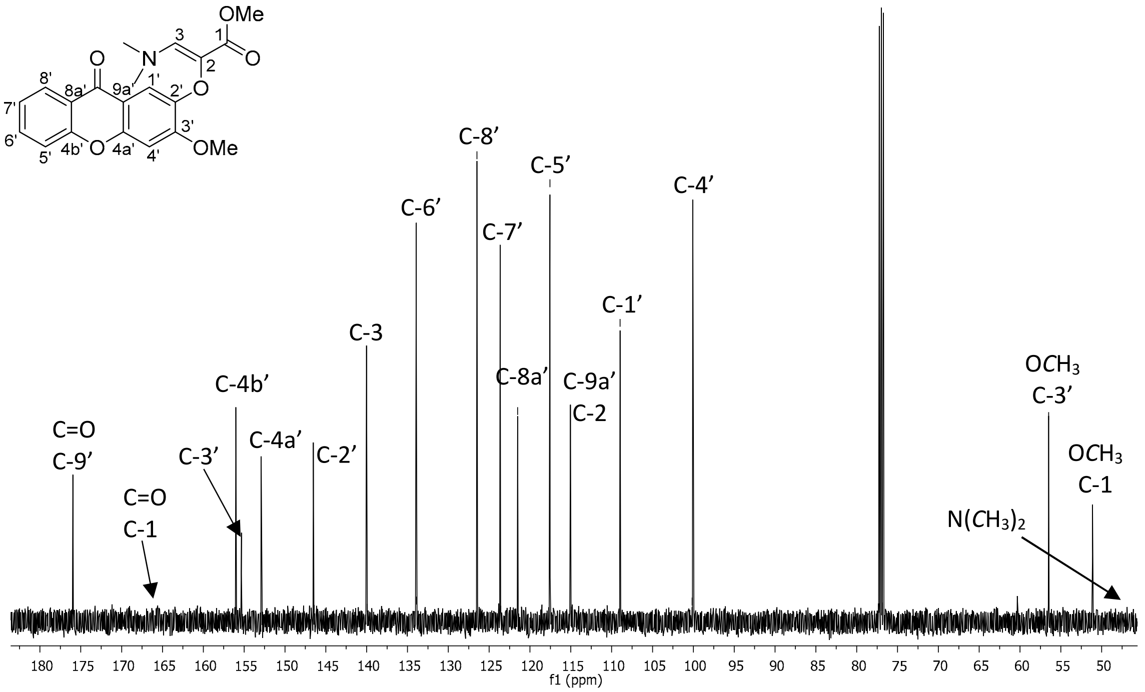

Figure S6. ^{13}C NMR (125 MHz, $CDCl_3$) spectrum of **5**.

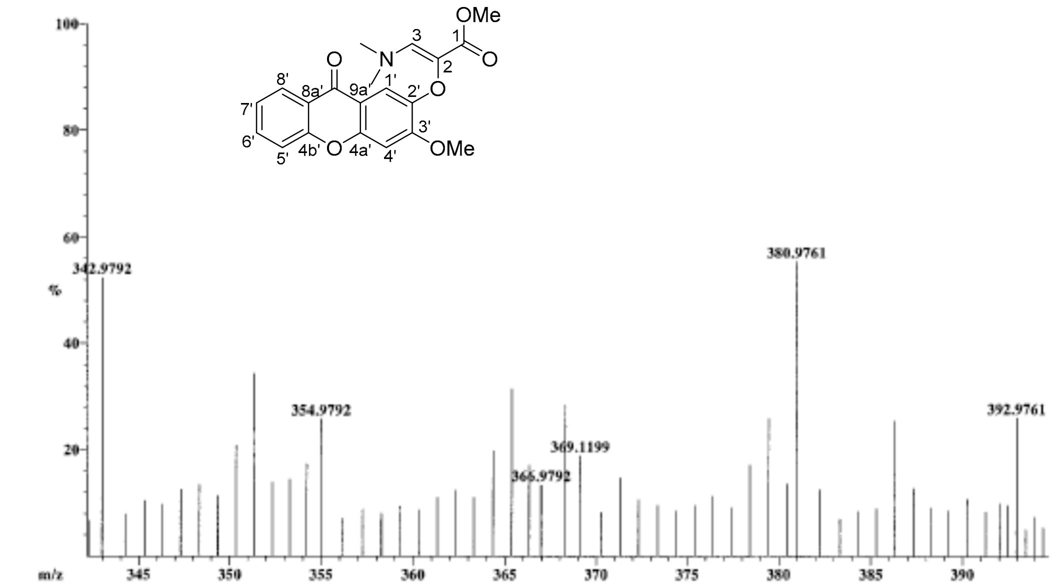

Selected Isotopes : $H_{0\text{-}19}C_{0\text{-}20}N_{0\text{-}1}O_{0\text{-}6}$ Error Limit : 5 ppm

Measured Mass	% Base	Formula	Calculated Mass	Error
369.1199	19.0%	$C_{20}H_{19}NO_6$	369.1212	-3.6

Figure S7. HRMS (EI) spectra of compound **9a**.

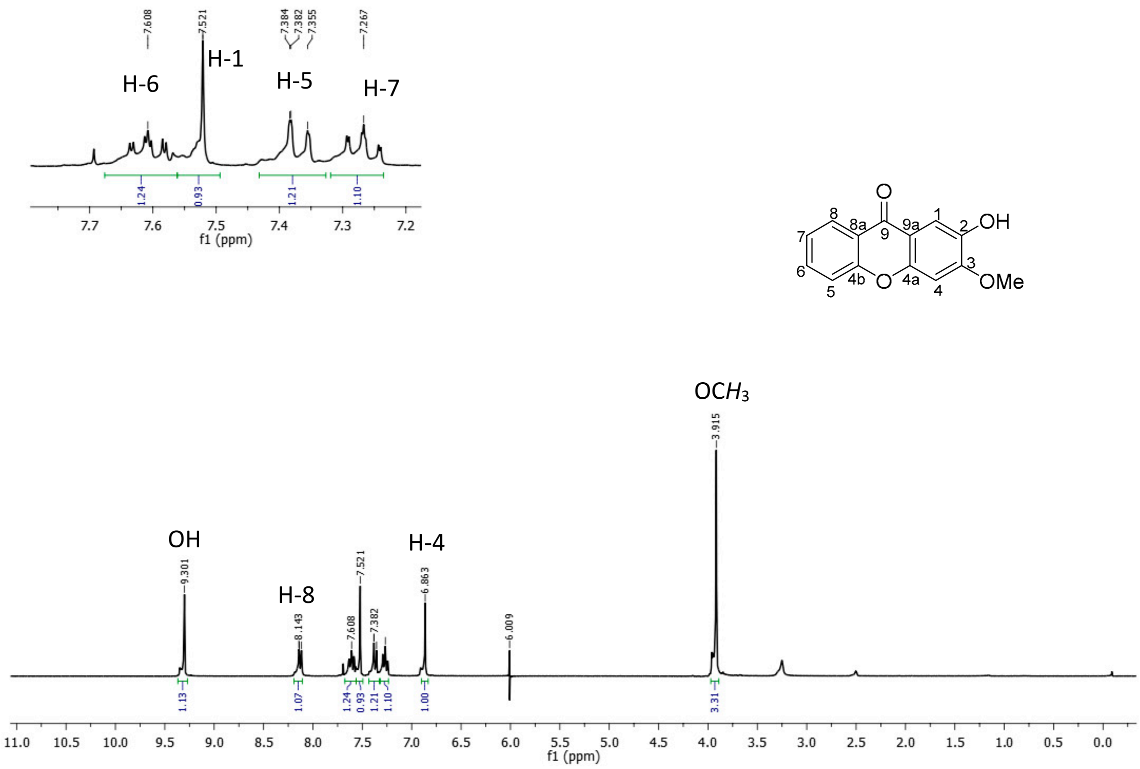

Figure S8. ^{1}H NMR (300 MHz, DMSO-d_6) spectrum of **1**.

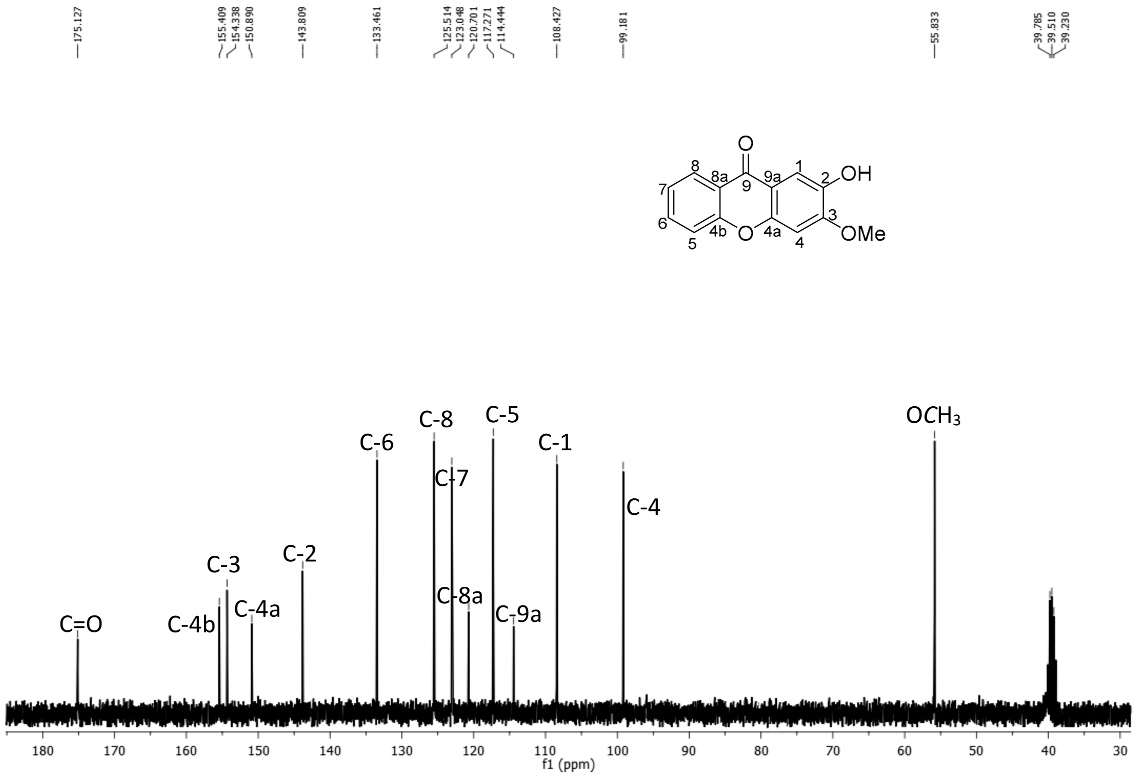

Figure S9. ^{13}C NMR (75 MHz, DMSO-d_6) spectrum of **1**.

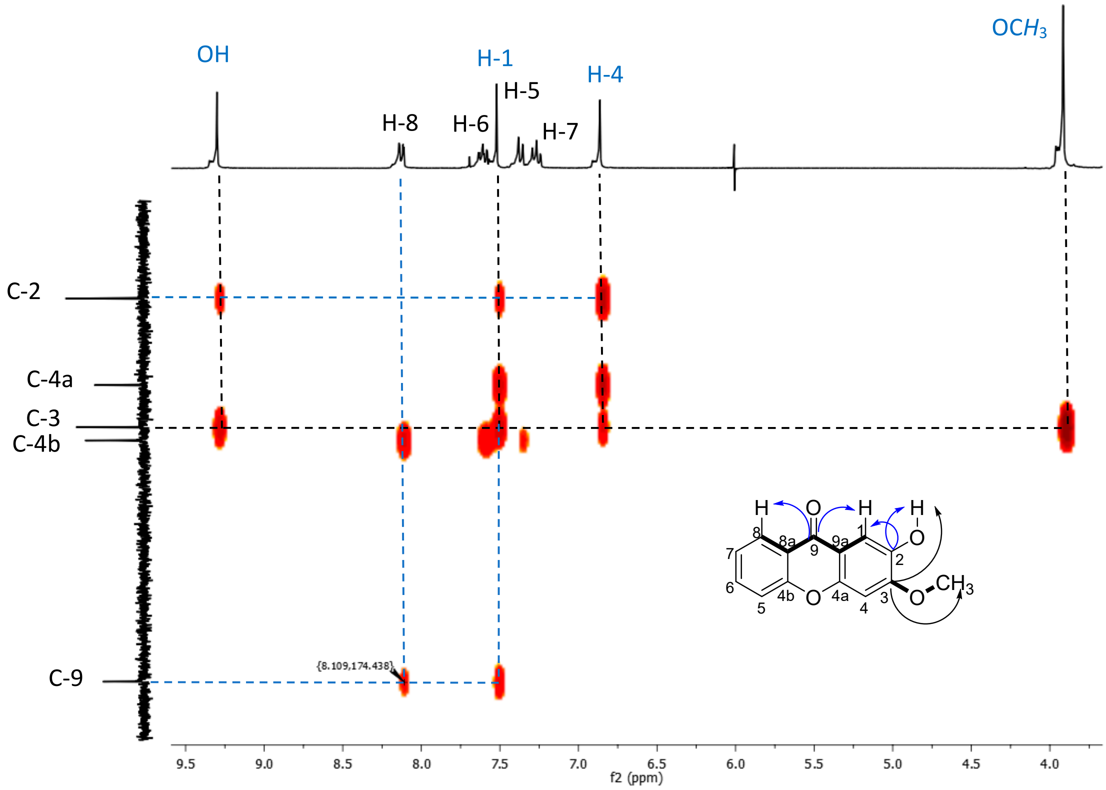

Figure S10. HMBC 2D NMR spectrum of **1**.

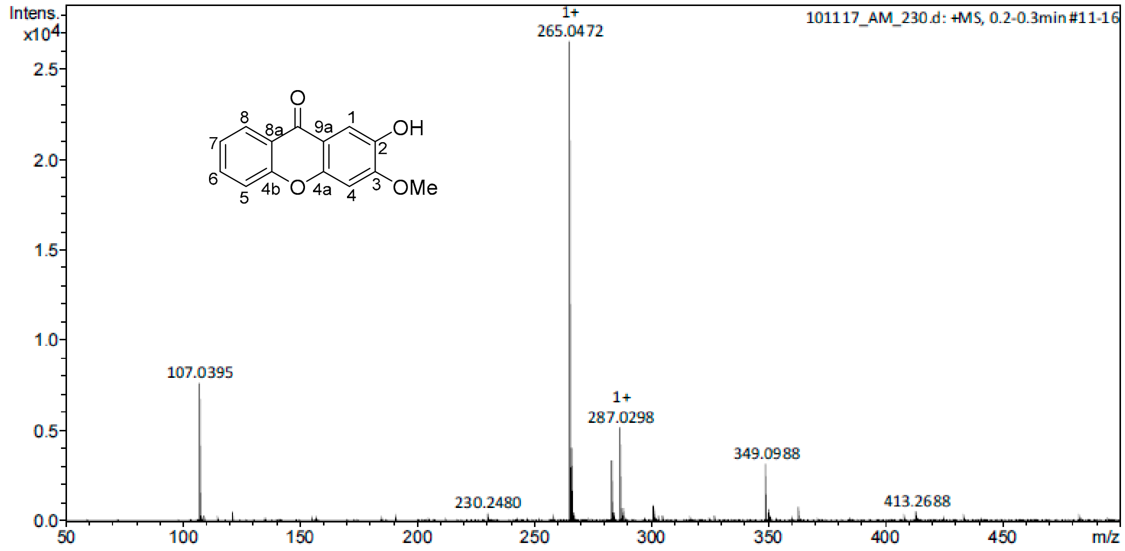

Figure S11. HRMS (ESI) spectra of compound **1**.

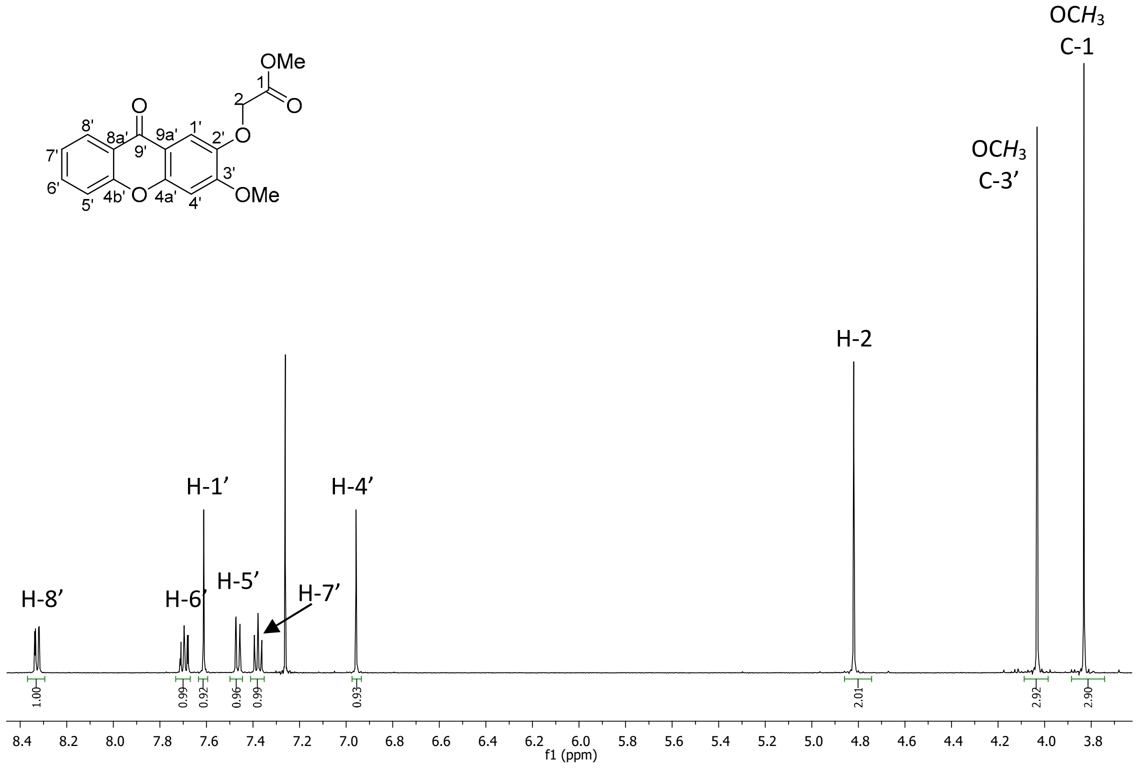

Figure S12. ^{1}H NMR (500 MHz, $CDCl_3$) spectrum of **6a**.

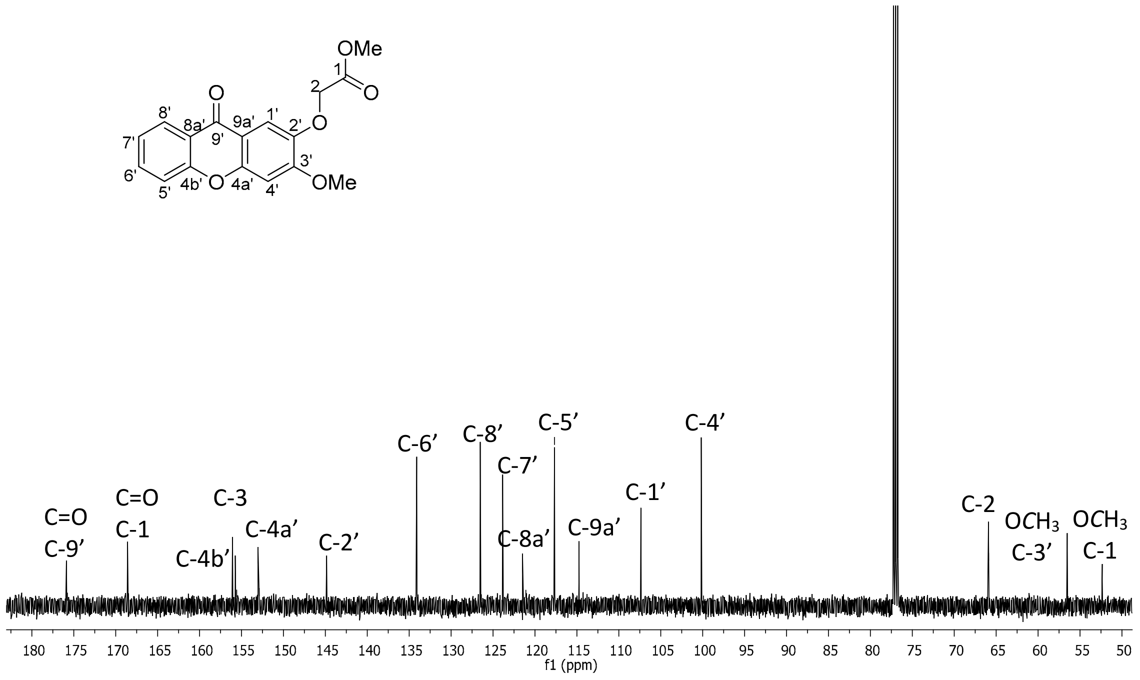

Figure S13. ^{13}C NMR (125 MHz, $CDCl_3$) spectrum of **6a**.

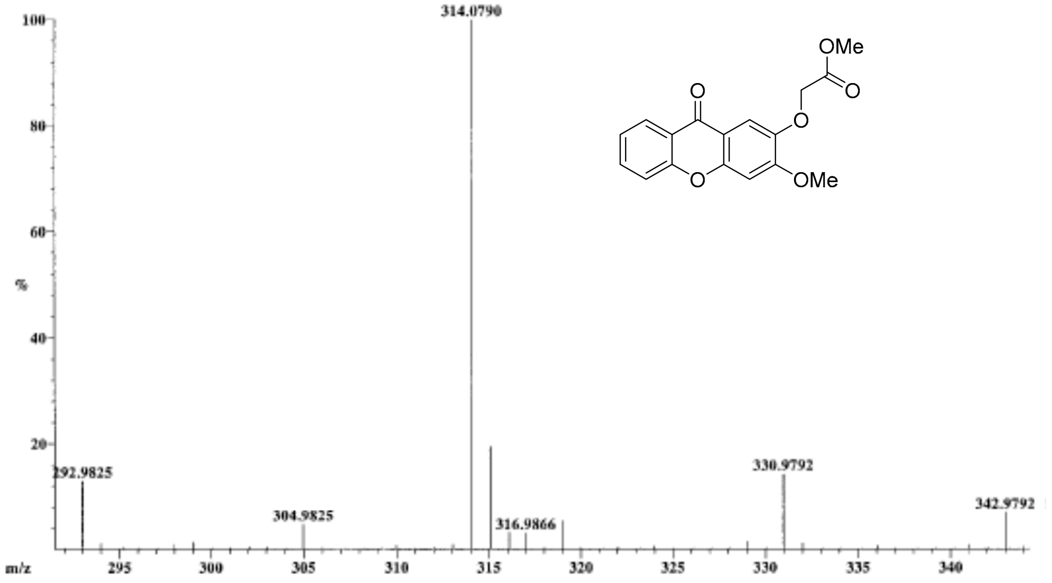

Selected Isotopes : $H_{0\text{-}14}C_{0\text{-}17}O_{0\text{-}6}$ Error Limit : 5 ppm

Measured Mass	% Base	Formula	Calculated Mass	Error
314.0790	100.0%	$C_{17}H_{14}O_6$	314.0790	-0.1

Figure S14. HRMS (EI) spectra of compound **6a**.

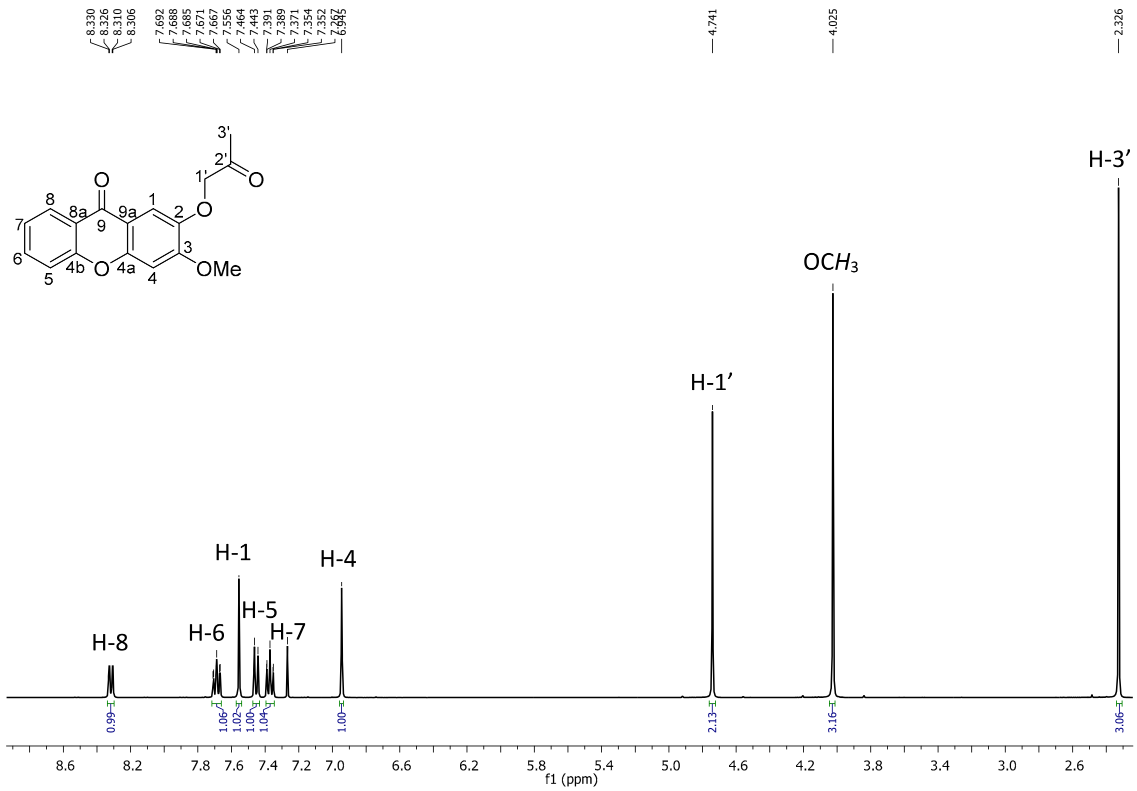

Figure S15. ^{1}H NMR (400 MHz, $CDCl_3$) spectrum of **6b**.

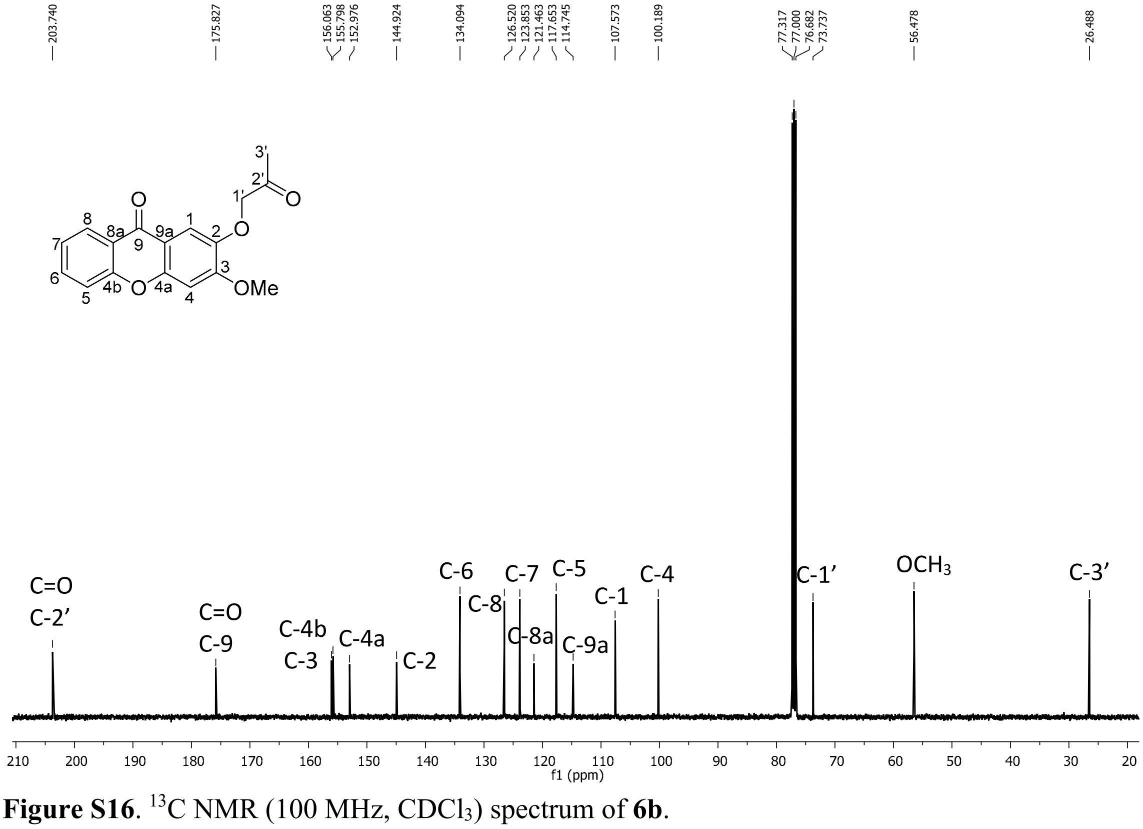

Figure S16. ^{13}C NMR (100 MHz, $CDCl_3$) spectrum of **6b**.

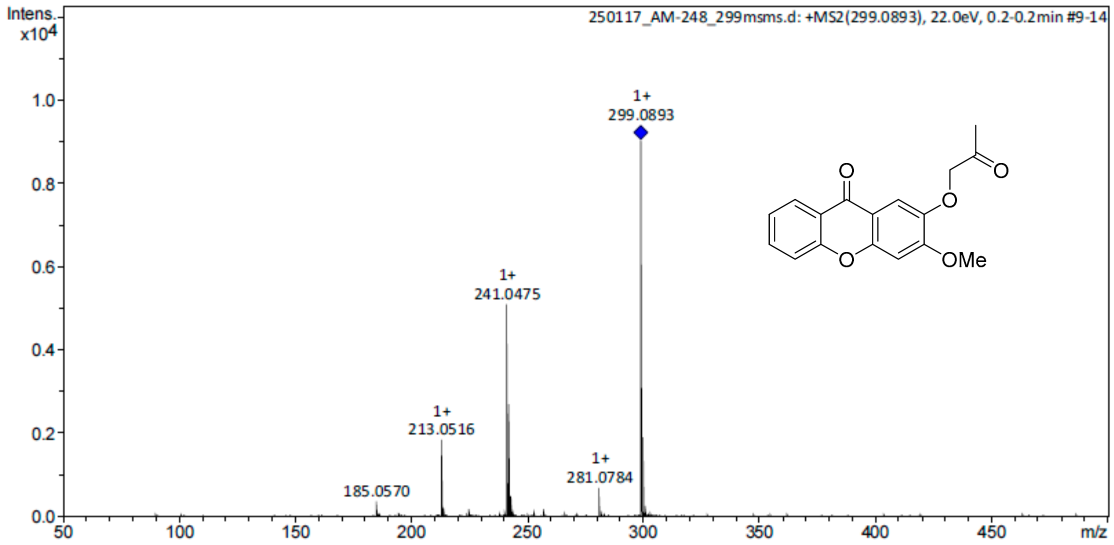

Figure S17. HRMS (ESI) spectra of compound **6b**.

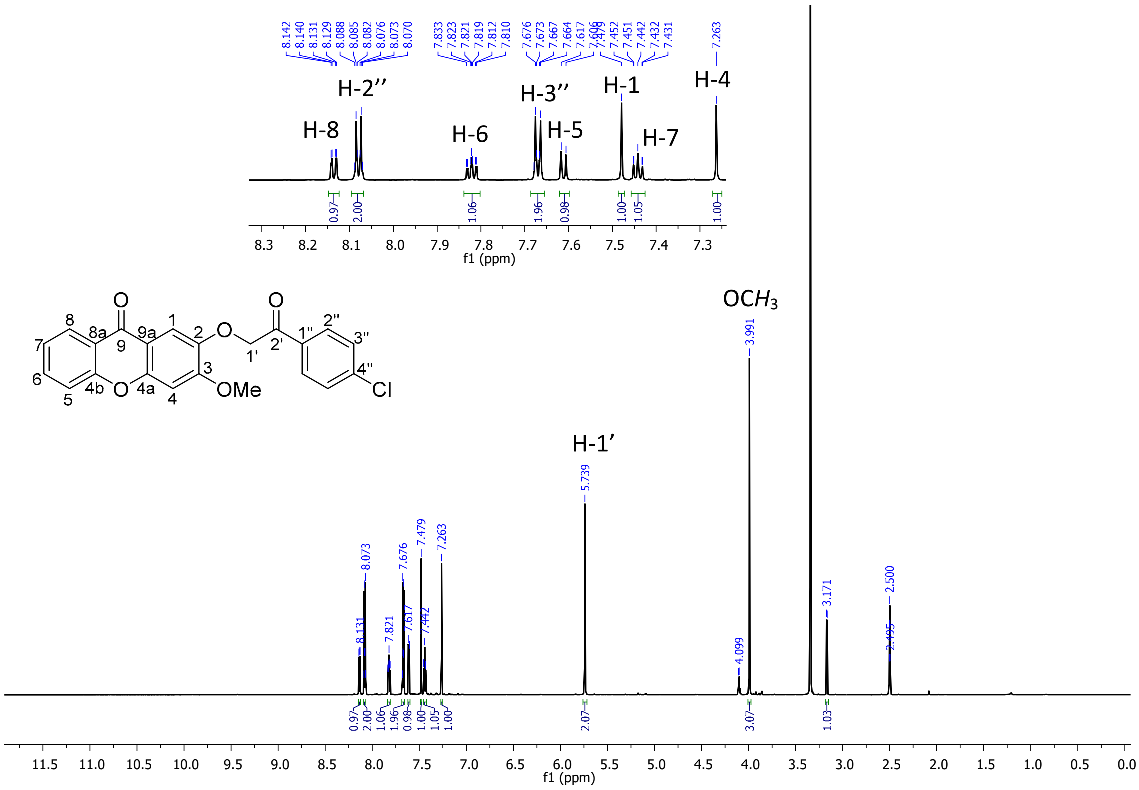

Figure S18. ^{1}H NMR (750 MHz, DMSO-d_6) spectrum of **6c**.

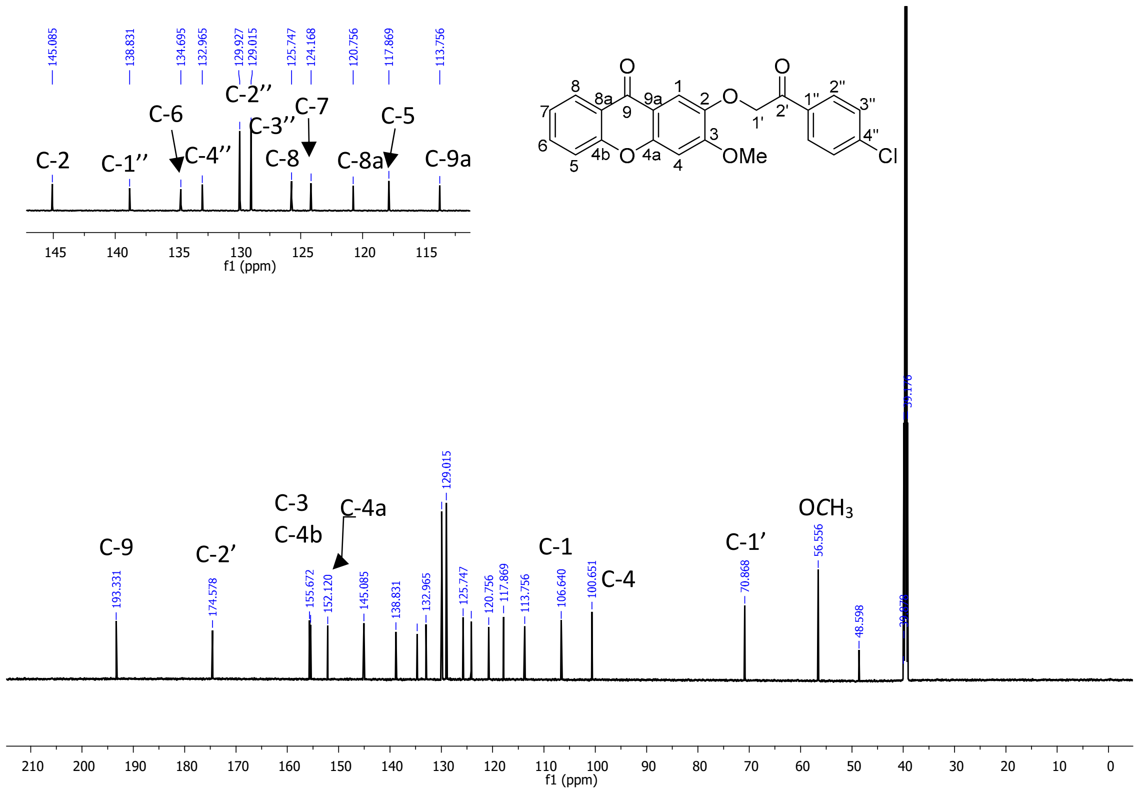

Figure S19. ^{13}C NMR (187.5 MHz, DMSO-d_6) spectrum of **6c**

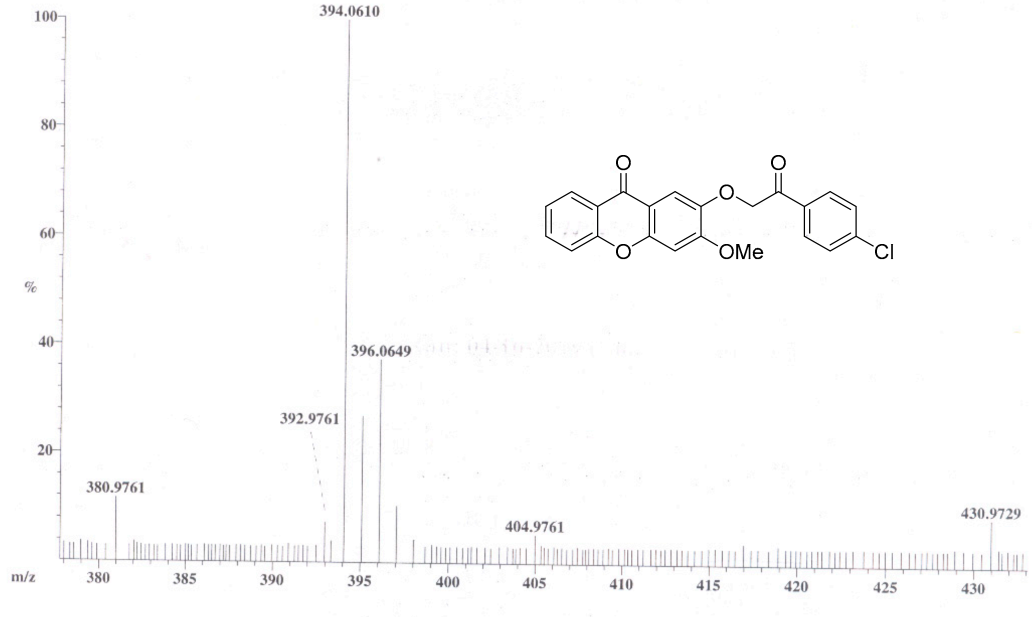

Selected Isotopes : $H_{0-15}C_{0-22}O_{0-5}Cl_{0-1}$

Error Limit : 5 ppm

Measured Mass	% Base	Formula	Calculated Mass	Error
394.0610	100.0%	$C_{22}H_{15}O_5Cl$	394.0608	0.5

Figure S20. HRMS (EI) spectra of compound **6c**.

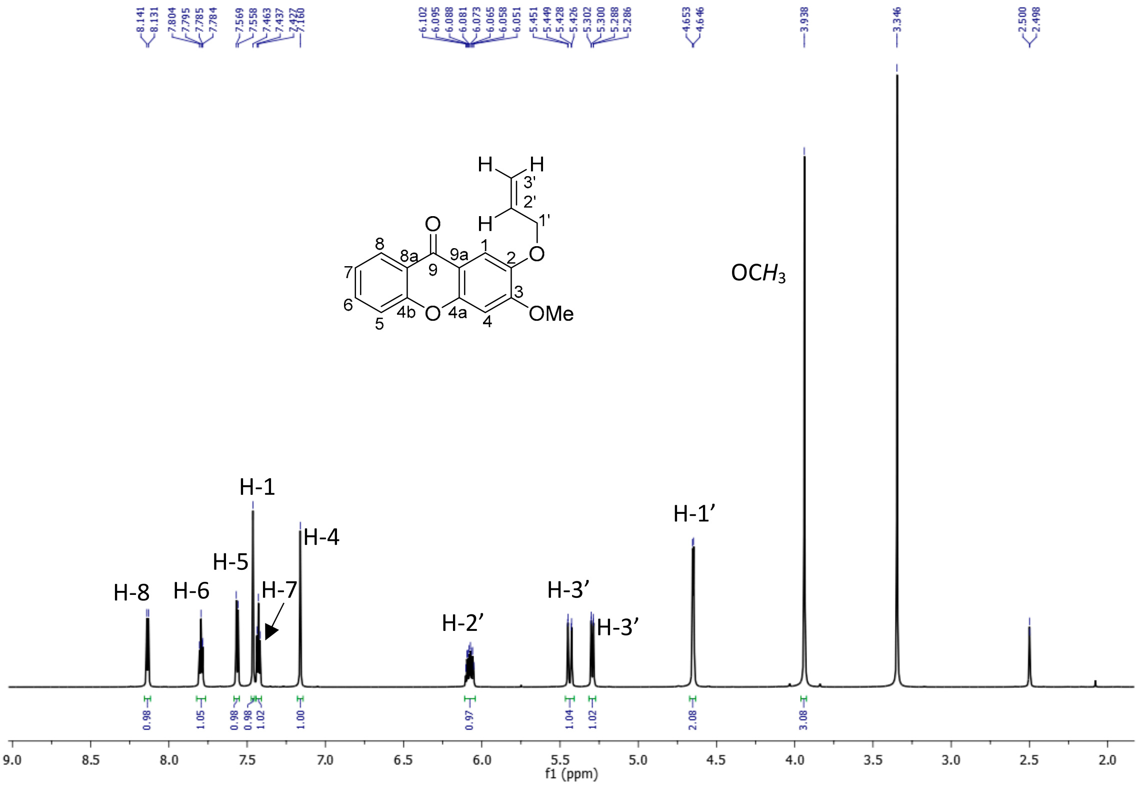

Figure S21. ^{1}H NMR (750 MHz, DMSO-d_6) spectrum of **6d**.

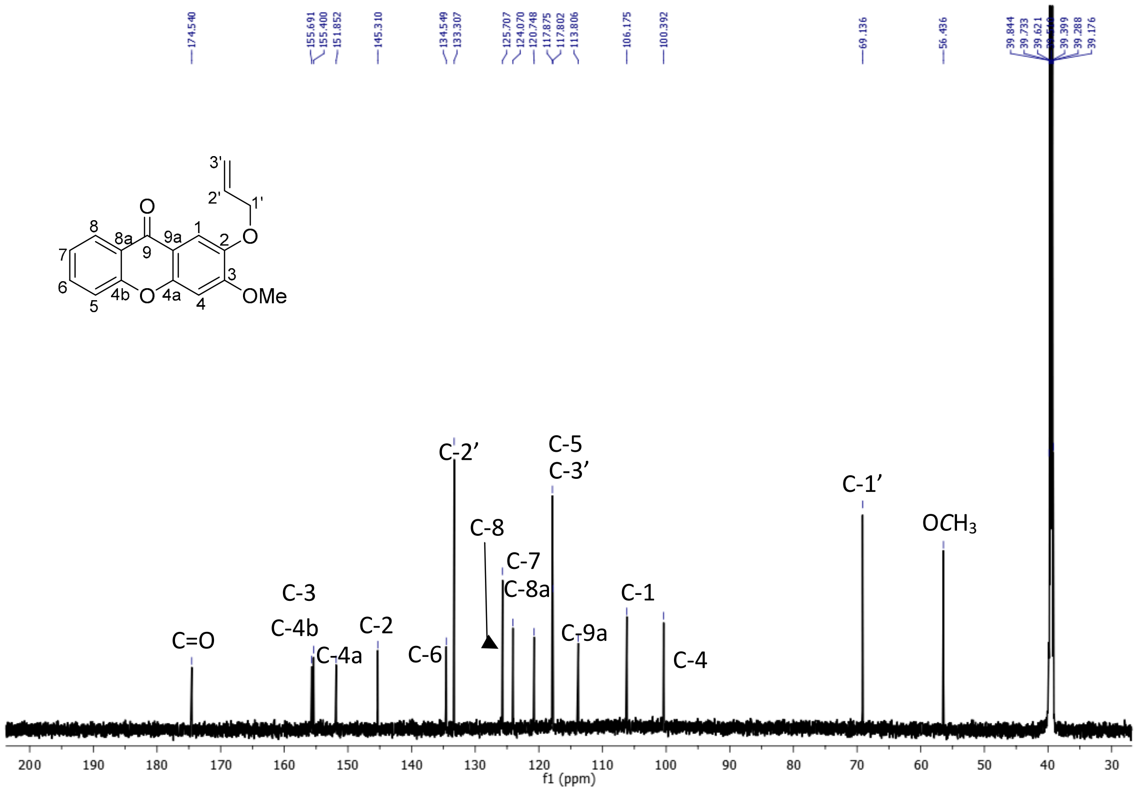

Figure S22. ^{13}C NMR (187.5 MHz, DMSO-d_6) spectrum of **6d**.

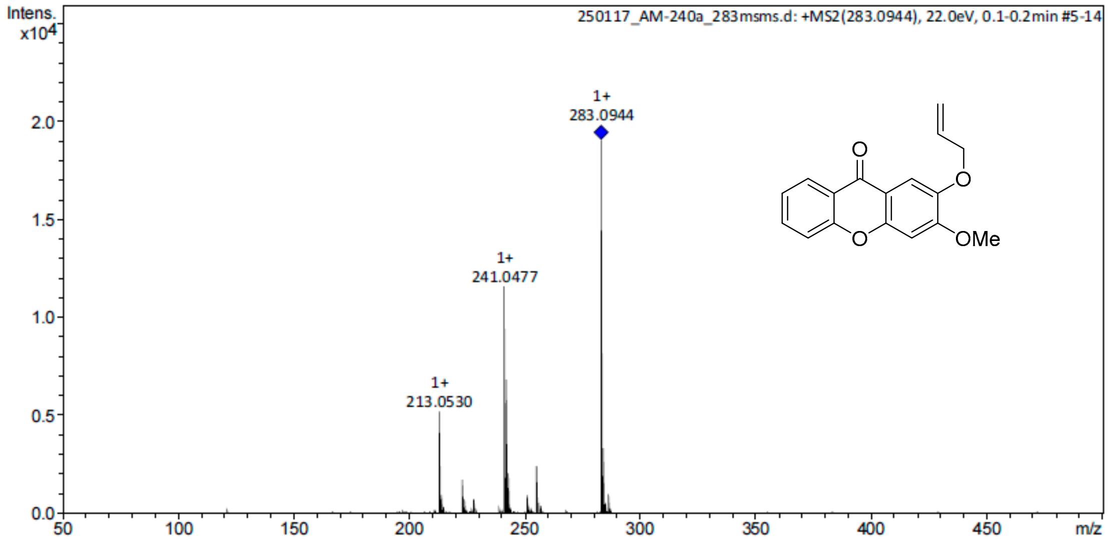

Figure S23. HRMS (ESI) spectra of compound **6d**.

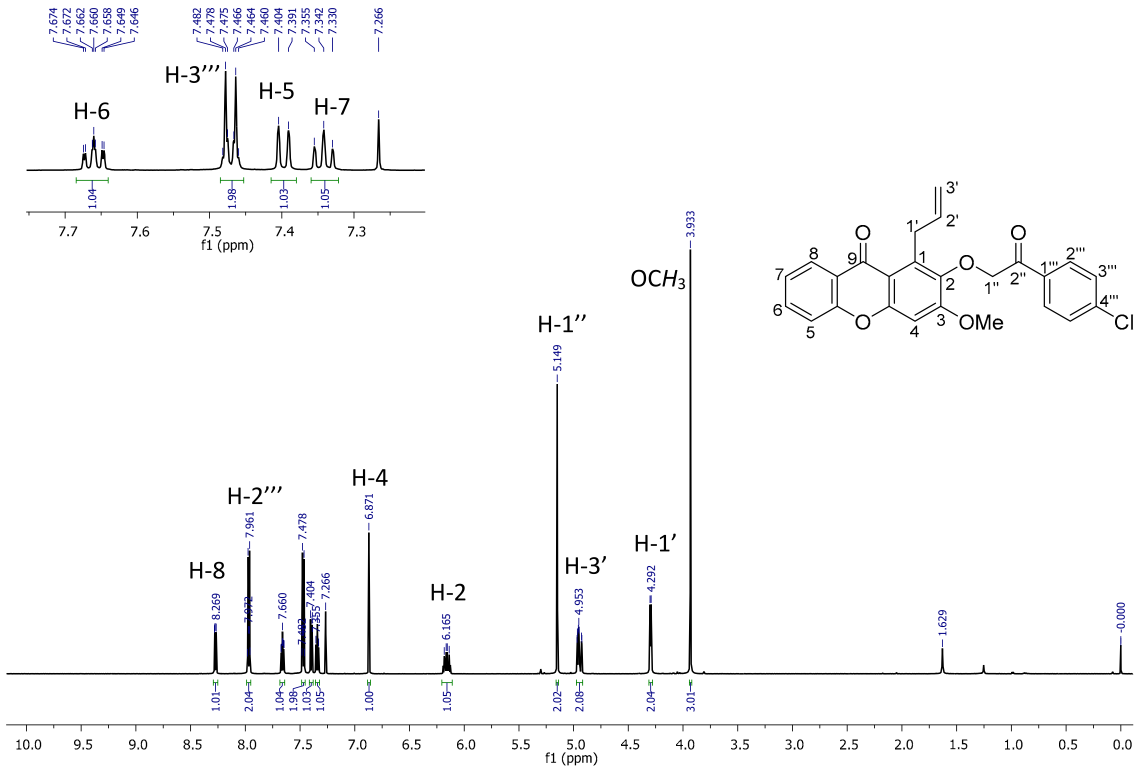

Figure S24. ^{1}H NMR (600 MHz, $CDCl_3$) spectrum of **6e**.

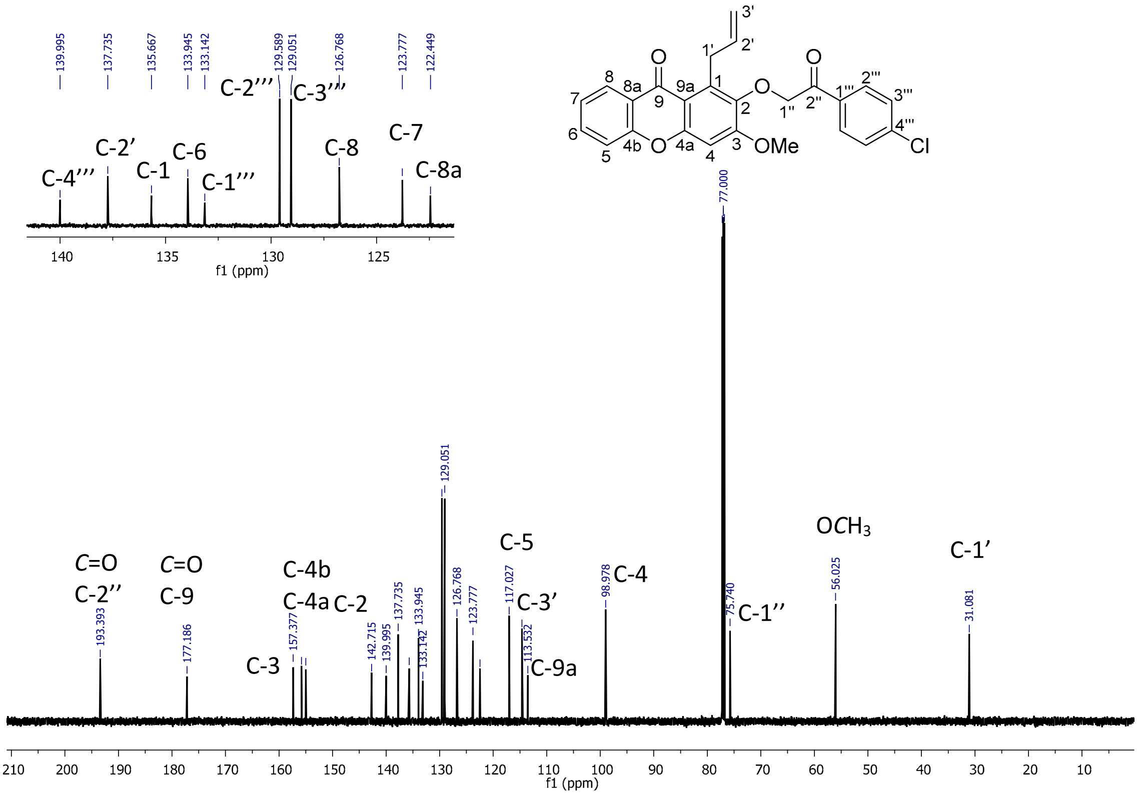

Figure S25. ^{13}C NMR (150 MHz, $CDCl_3$) spectrum of **6e**.

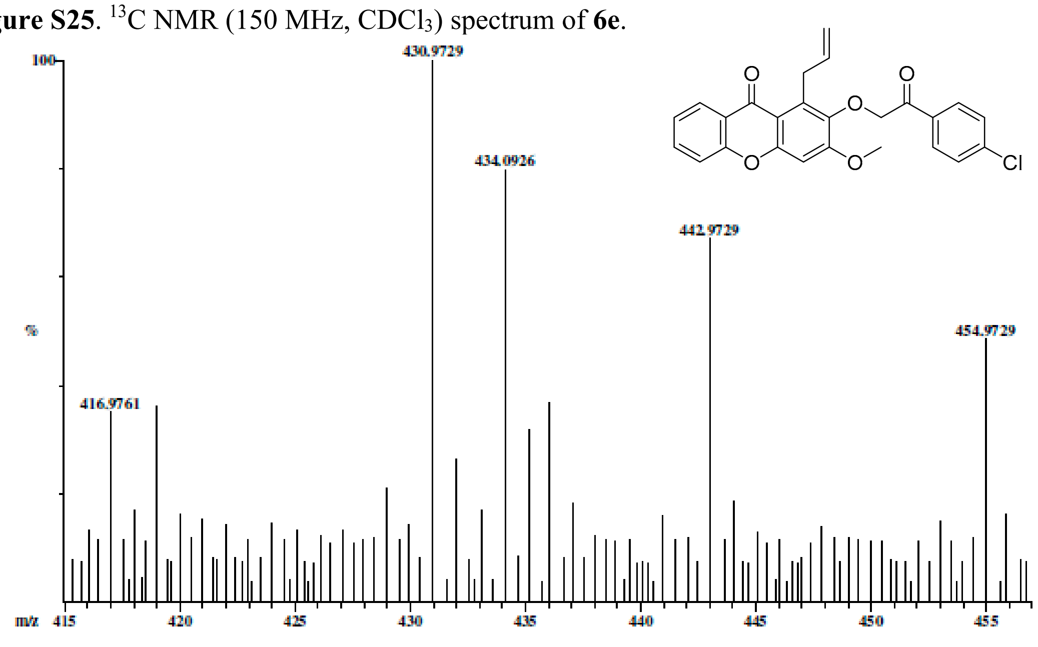

Selected Isotopes : $H_{0-19}C_{0-25}O_{0-5}Cl_{0-1}$

Error Limit : 5 ppm

Measured Mass	% Base	Formula	Calculated Mass	Error
434.0926	79.8%	$C_{25}H_{19}O_5Cl$	434.0921	1.2

Figure S26. HRMS (EI) spectra of compound **6e**.

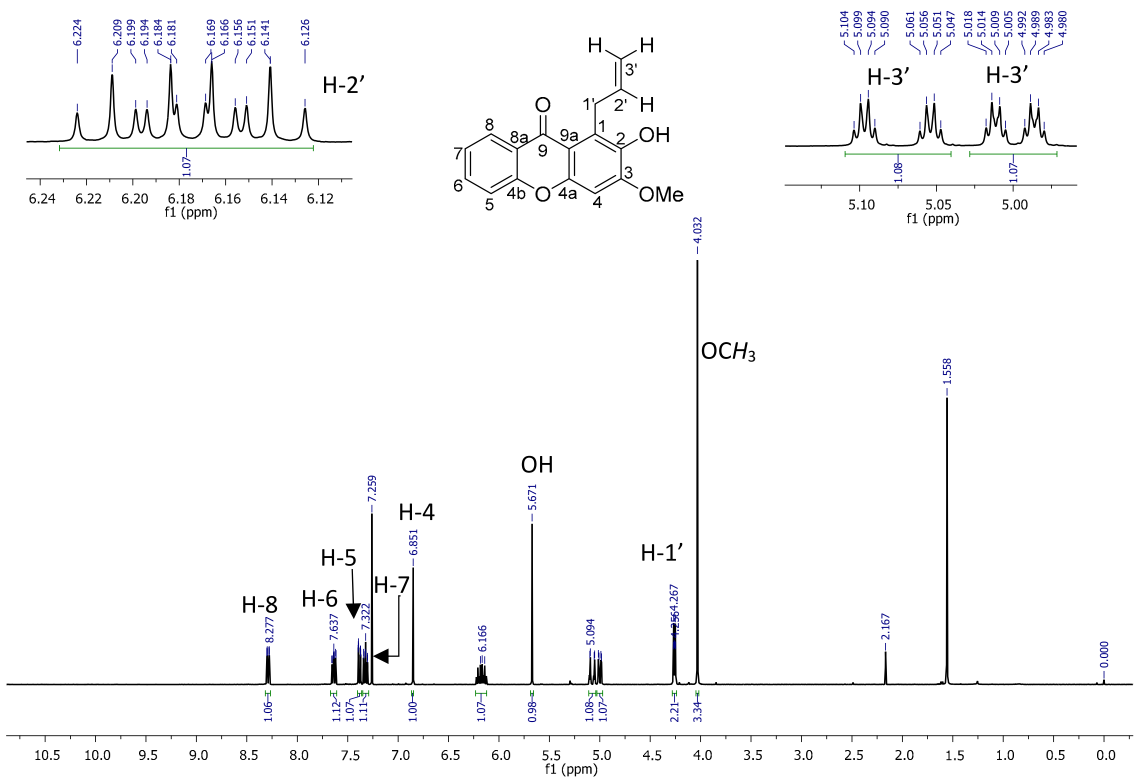

Figure S27. ^{1}H NMR (750 MHz, $CDCl_3$) spectrum of **7**.

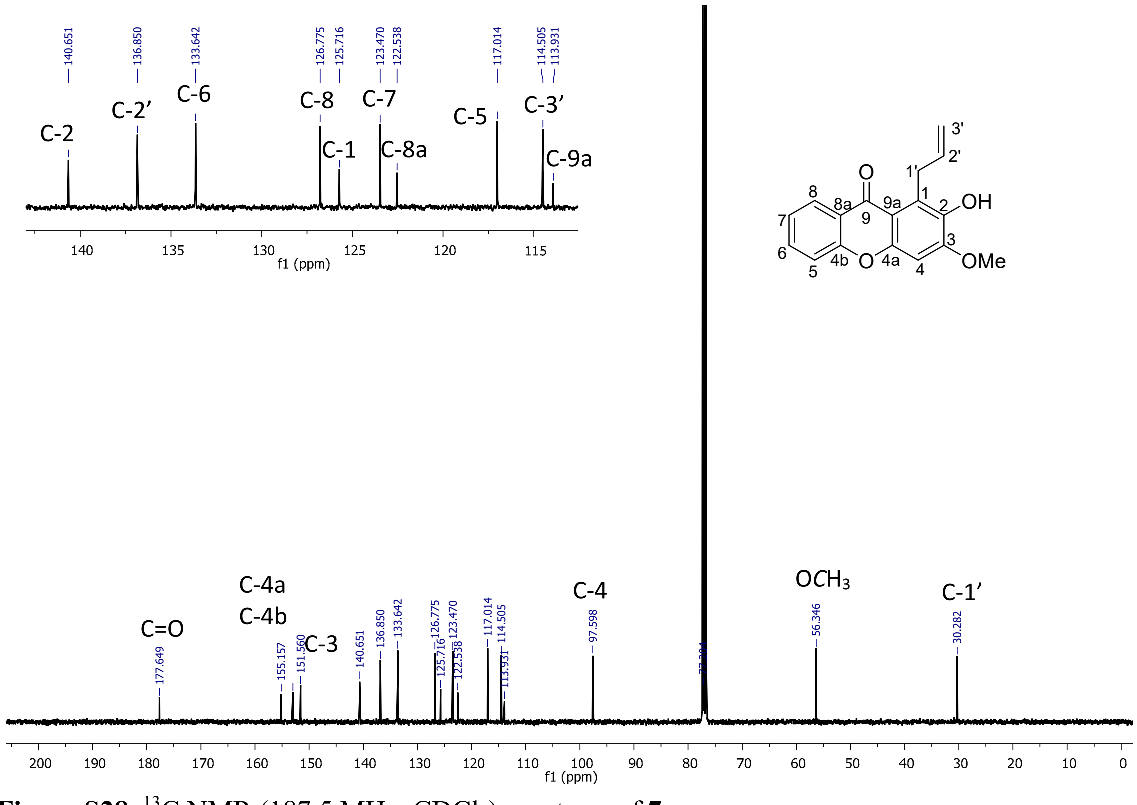

Figure S28. ^{13}C NMR (187.5 MHz, $CDCl_3$) spectrum of **7**.

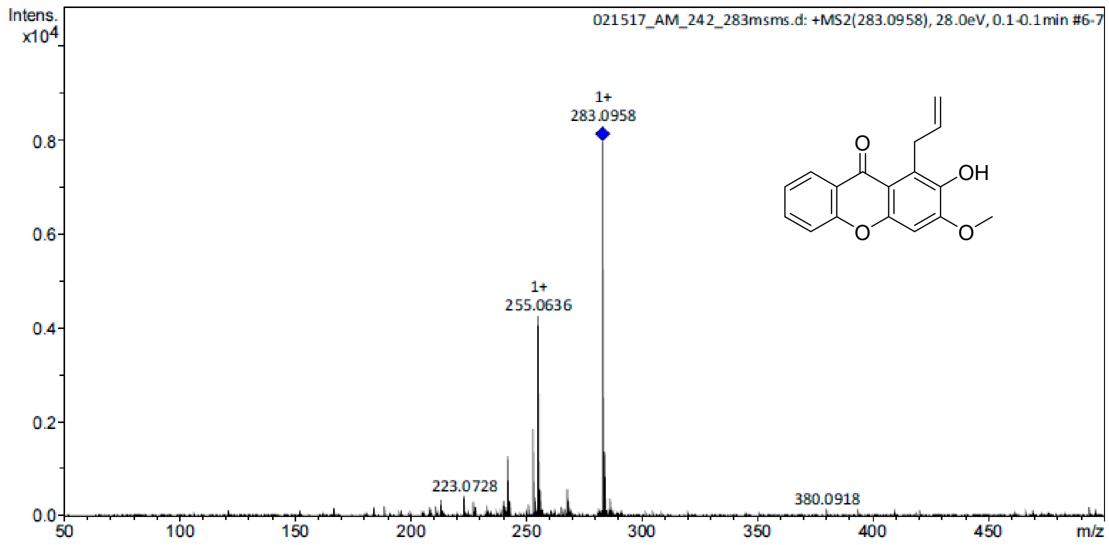

Figure S29. HRMS (ESI) spectra of compound **7**.

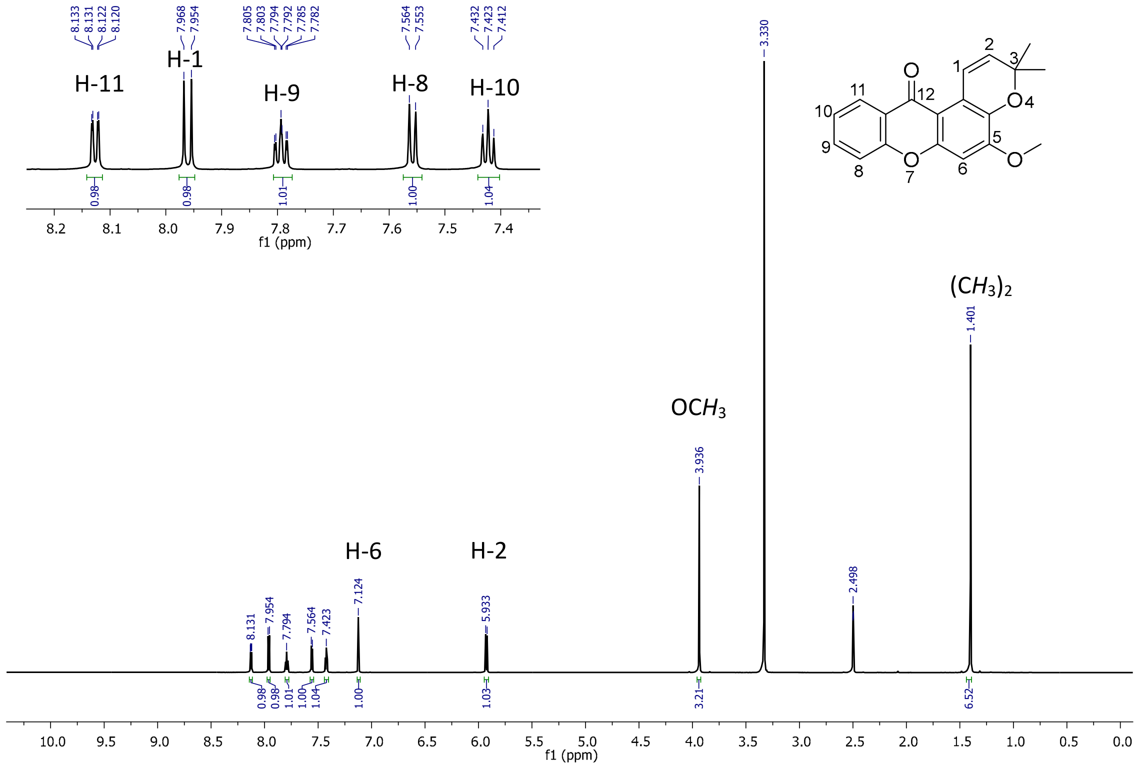

Figure 30 ^{1}H NMR (750 MHz, DMSO-d_6) spectrum of **8**.

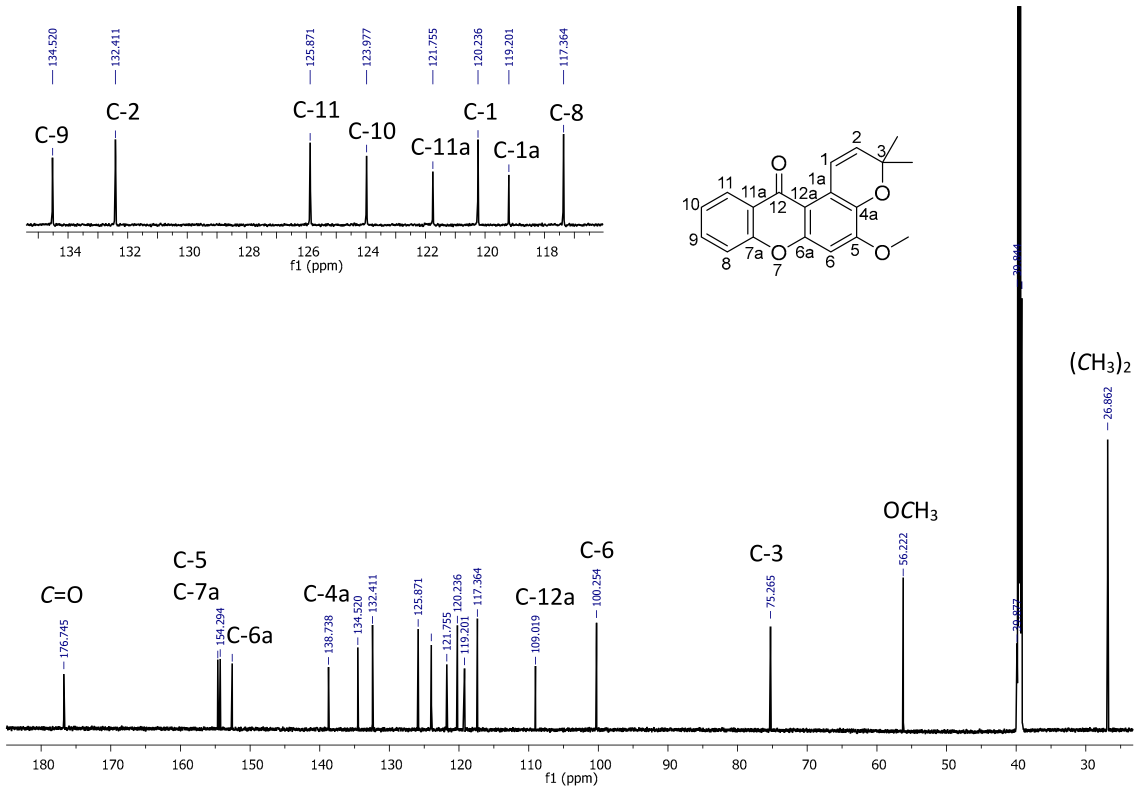

Figure S31. ^{13}C NMR (187.5 MHz, DMSO-d_6) spectrum of **8**.

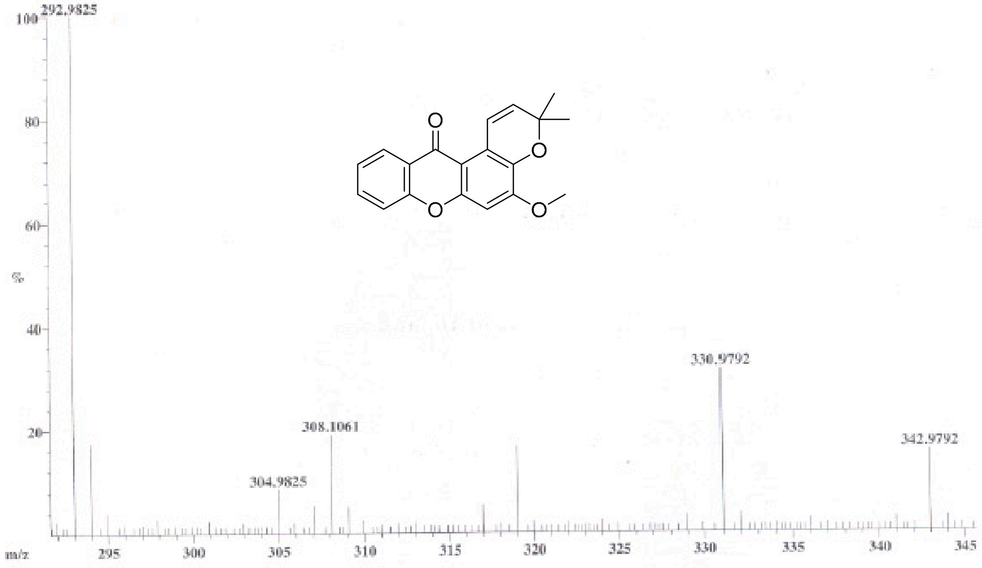

Selected Isotopes : $H_{0-16}C_{0-19}O_{0-4}$

Error Limit : 5 ppm

Measured Mass	% Base	Formula	Calculated Mass	Error
308.1061	19.0%	$C_{19}H_{16}O_4$	308.1049	4.0

Figure S32. HRMS (EI) spectra of compound **8**.

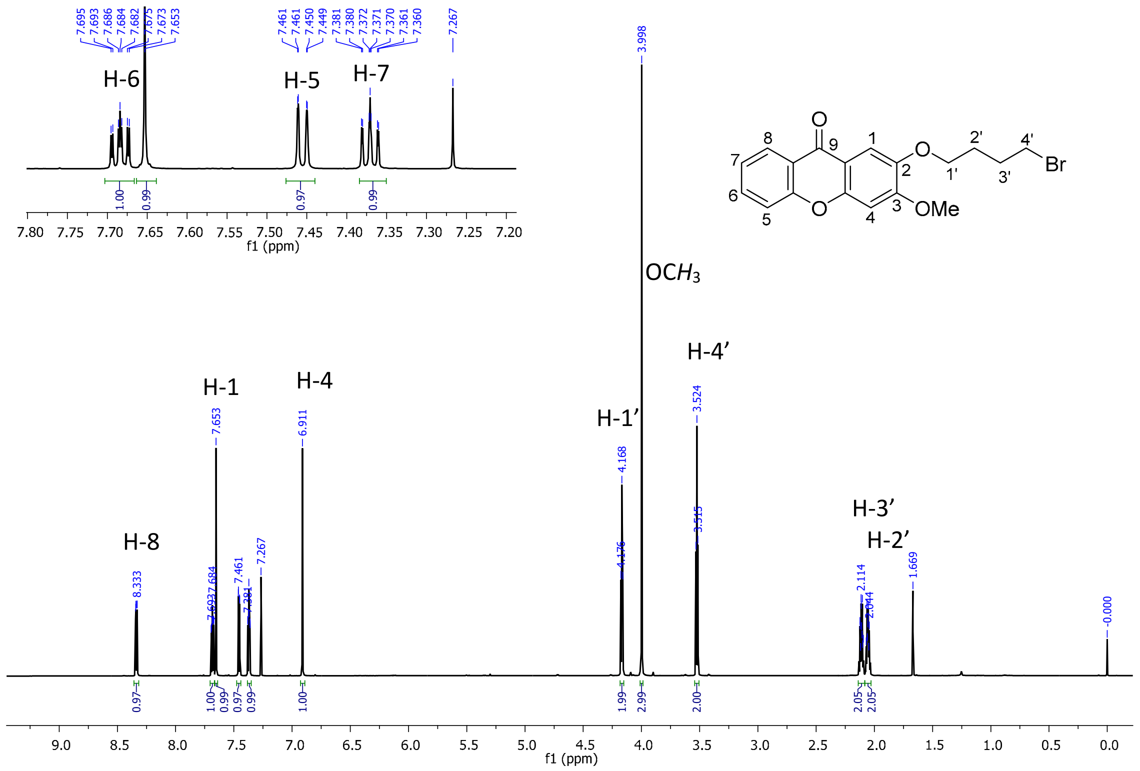

Figure S33. ^{1}H NMR (750 MHz, CDCl$_3$) spectrum of **9a**.

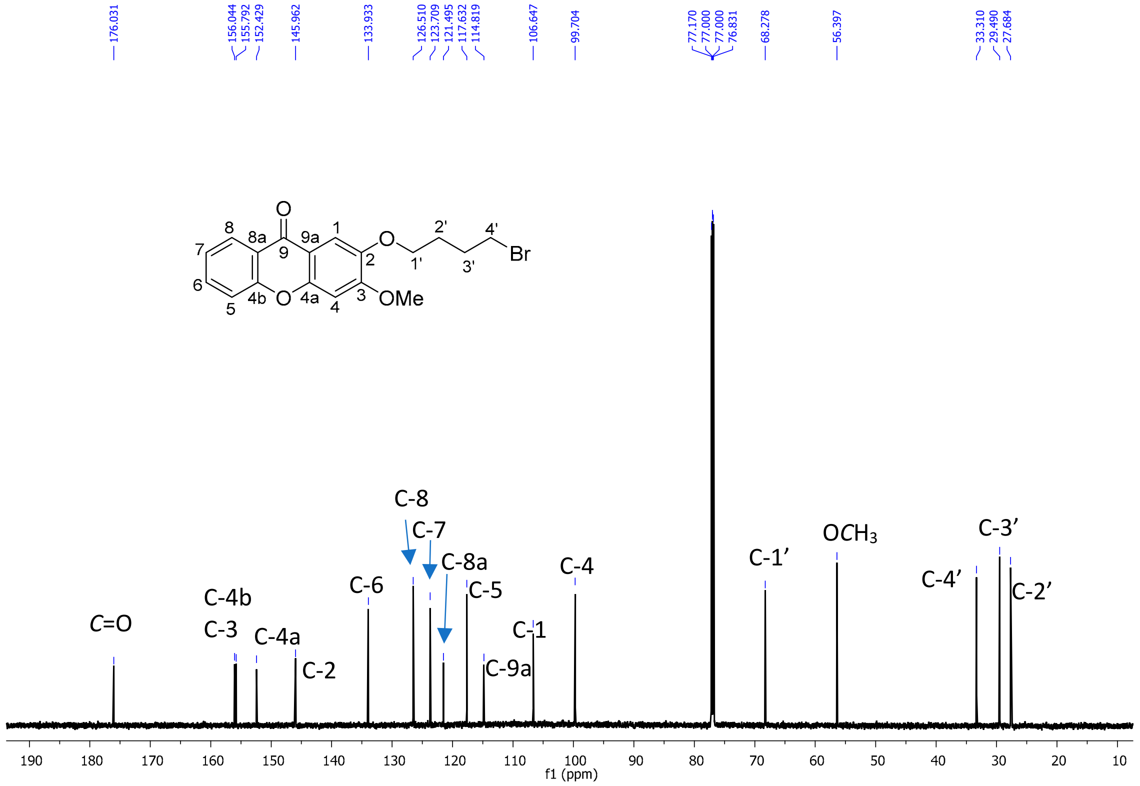

Figure S34. ^{13}C NMR (187.5 MHz, CDCl$_3$) spectrum of **9a**

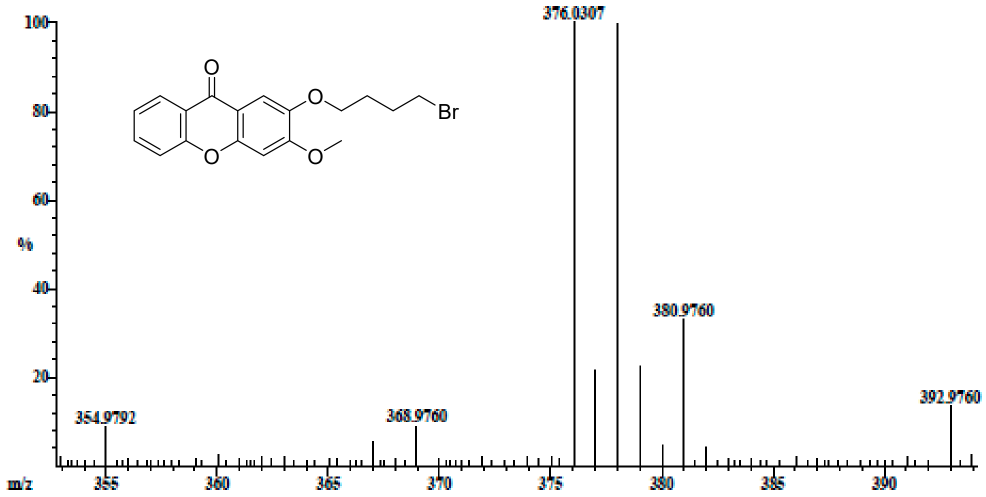

Selected Isotopes : $H_{0-17}C_{0-18}O_{0-4}Br_{0-1}$ Error Limit : 5 ppm Unsaturation Limits : 0 to 50

Measured Mass	% Base	Formula	Calculated Mass	Error	Unsaturation
376.0307	100.0%	$C_{18}H_{17}O_4Br$	376.0310	-0.8	10.0

Figure S35. HRMS (EI) spectra of compound **9a**.

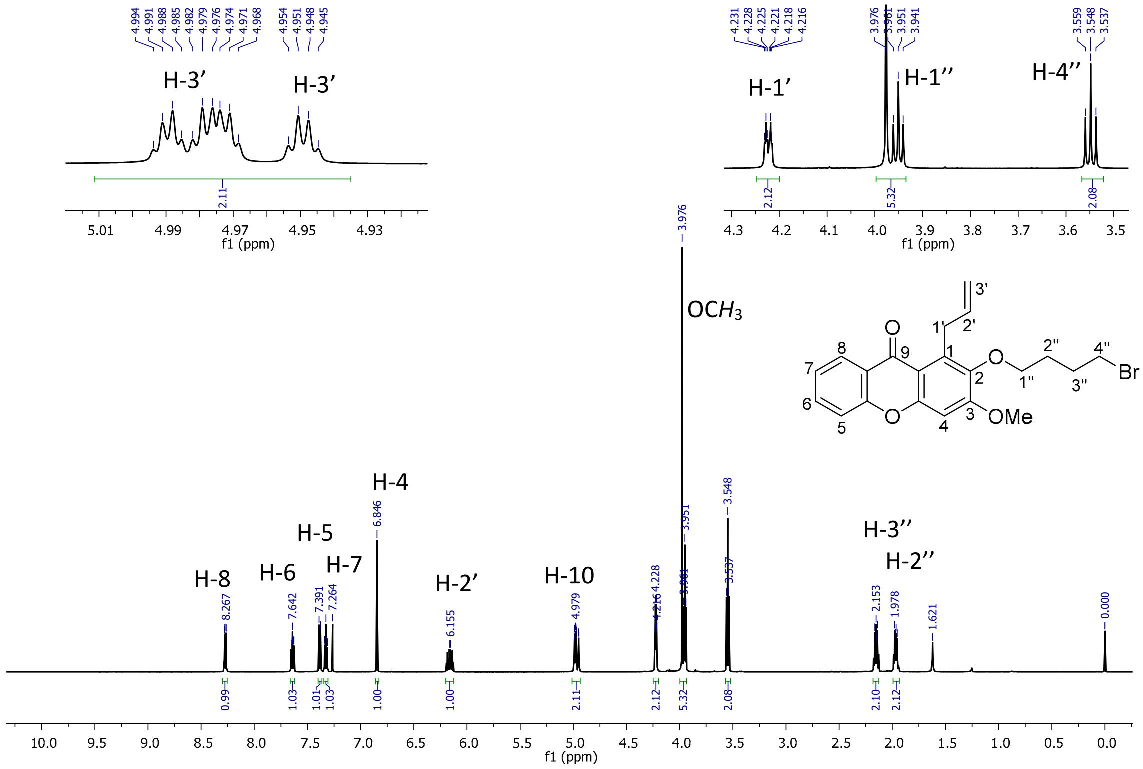

Figure S36. ^{1}H NMR (600 MHz, $CDCl_3$) spectrum of **9b**.

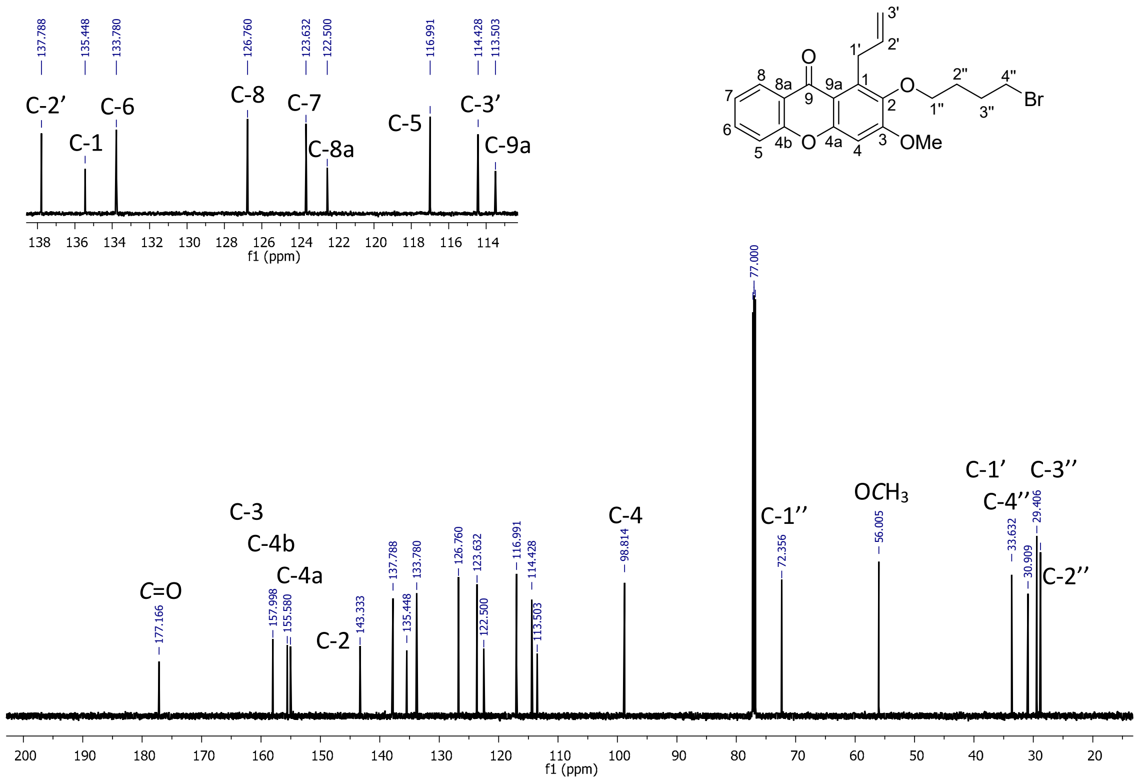

Figure S37. ^{13}C NMR (150 MHz, $CDCl_3$) spectrum of **9b**.

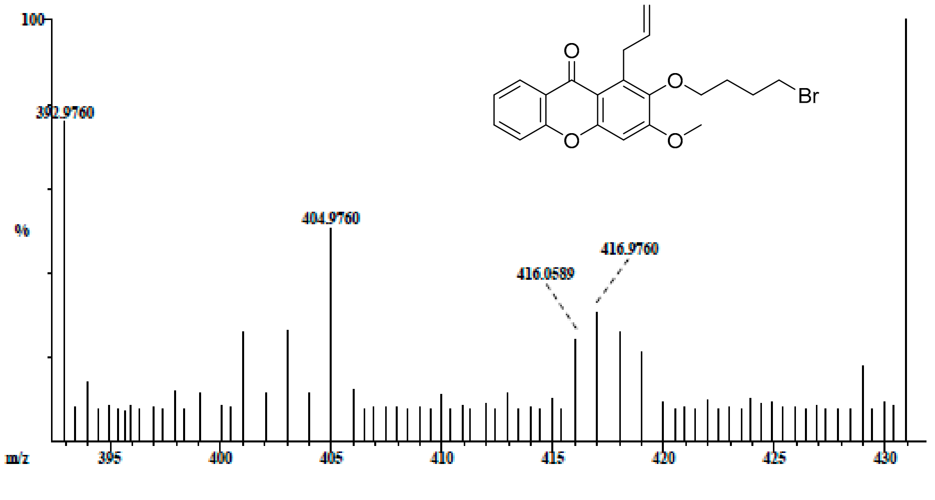

Selected Isotopes : $H_{0-21}C_{0-21}O_{0-4}Br_{0-1}$ Error Limit : 10 ppm Unsaturation Limits : 0 to 50

Measured Mass	% Base	Formula	Calculated Mass	Error	Unsaturation
416.0589	24.2%	$C_{21}H_{21}O_4Br$	416.0623	-8.2	11.0

Figure S38. HRMS (EI) spectra of compound **9b**.

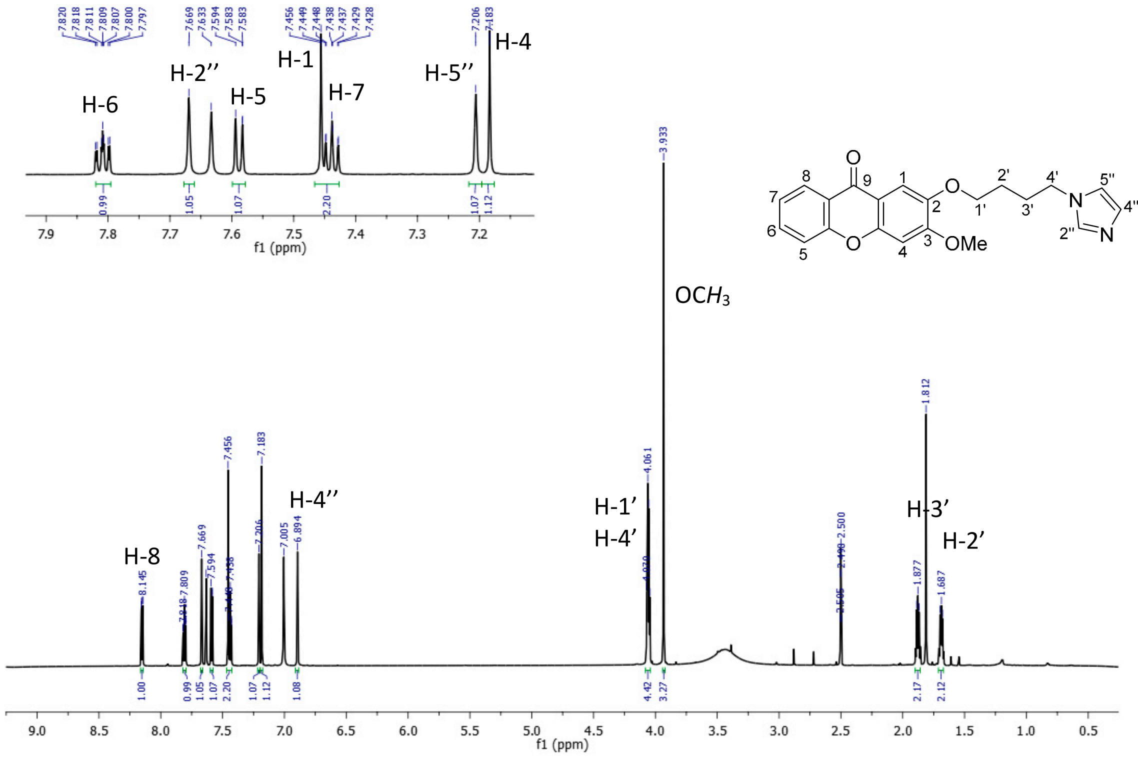

Figure S39. ^{1}H NMR (750 MHz, DMSO-d_6) spectrum of **10a**.

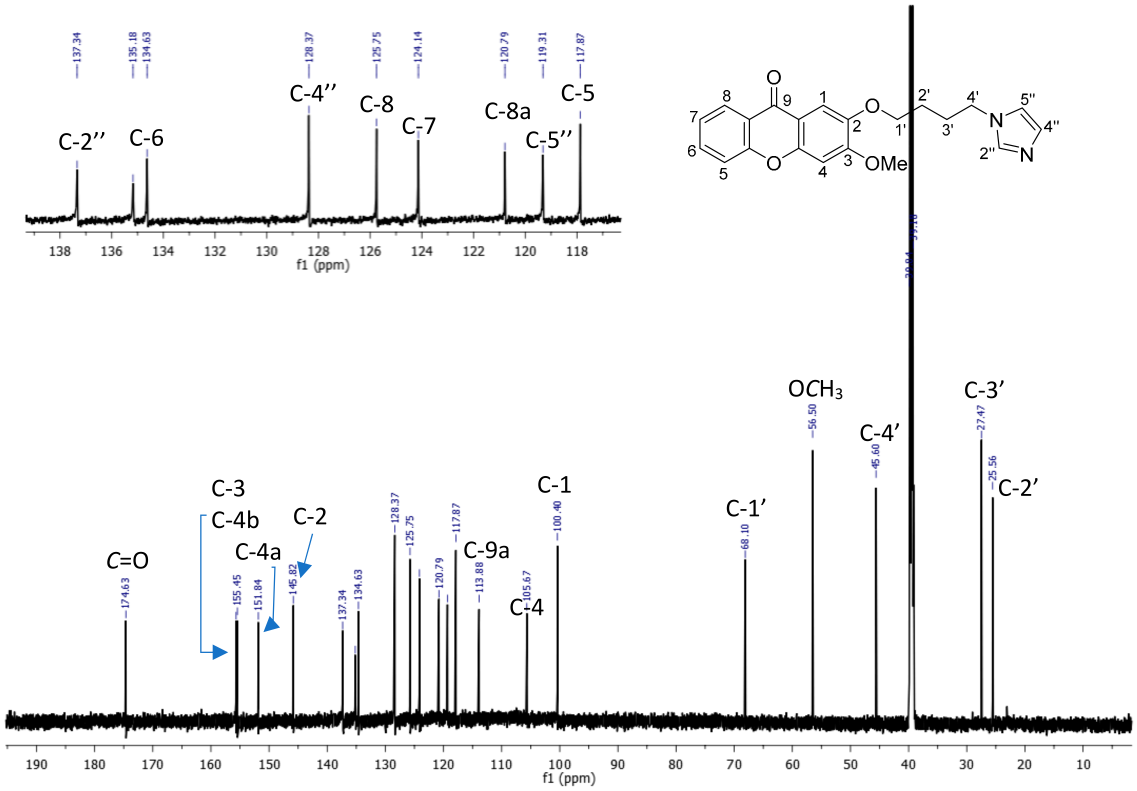

Figure S40. ^{13}C NMR (187.5 MHz, DMSO-d_6) spectrum of **10a**.

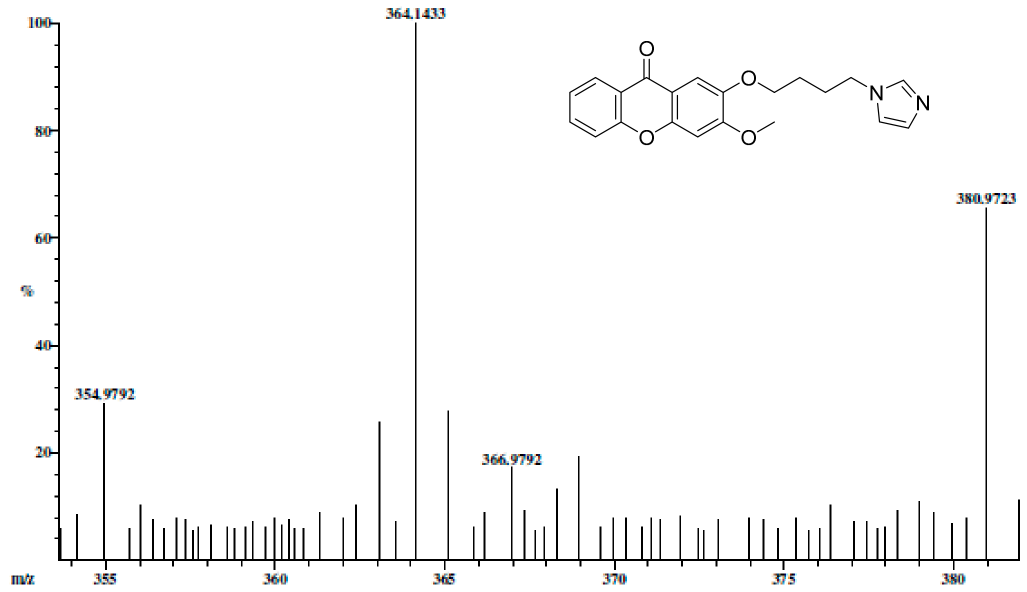

Selected Isotopes : $H_{0-20}C_{0-21}N_{0-2}O_{0-4}$ Error Limit : 5 ppm

Measured Mass	% Base	Formula	Calculated Mass	Error
364.1433	100.0%	$C_{21}H_{20}N_2O_4$	364.1423	2.7

Figure S41. HRMS (EI) spectra of compound **10a**.

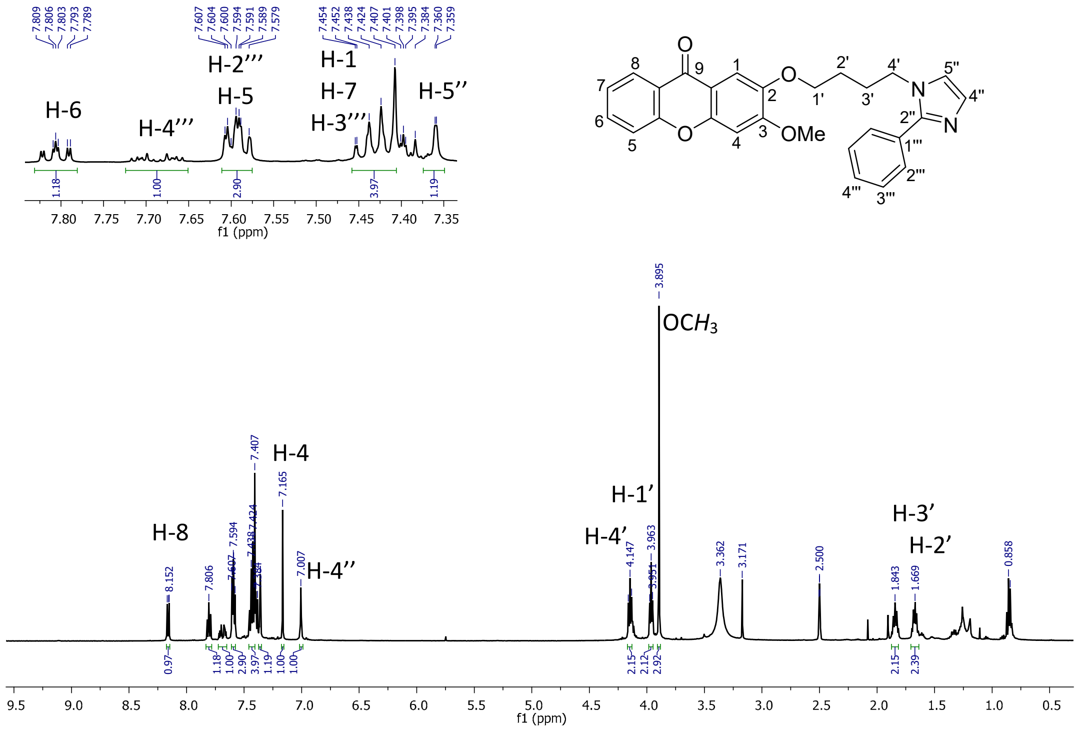

Figure S42. ^{1}H NMR (500 MHz, DMSO-d_6) spectrum of **10b**.

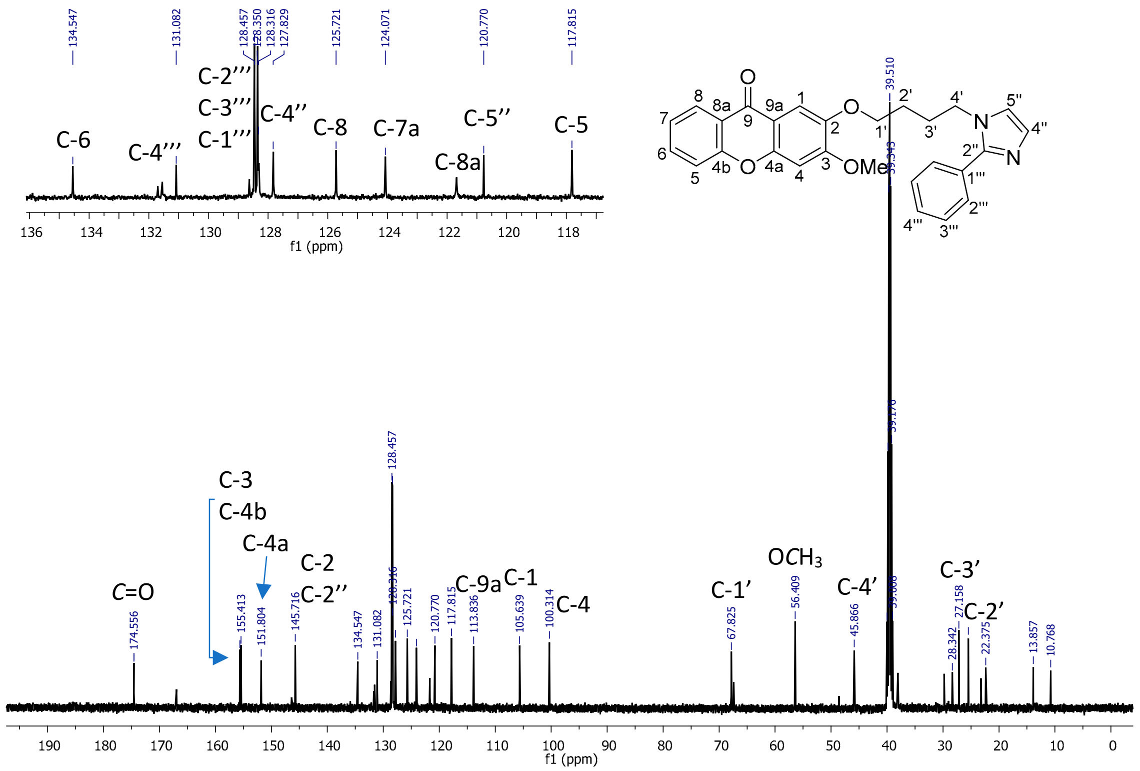

Figure S43. ^{13}C NMR (125 MHz, DMSO-d_6) spectrum of **10b**.

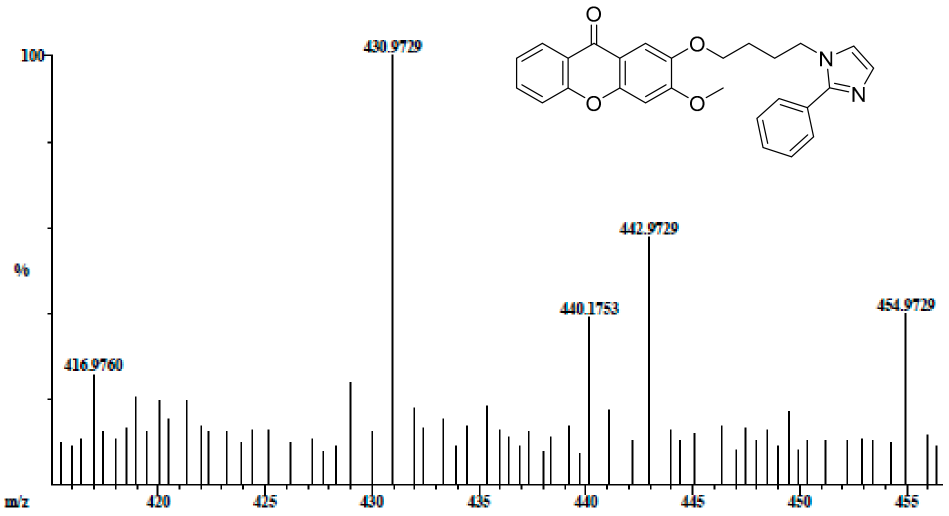

Selected Isotopes : $H_{0-24}C_{0-27}N_{0-2}O_{0-4}$ Error Limit : 5 ppm Unsaturation Limits : 0 to 50

Measured Mass	% Base	Formula	Calculated Mass	Error	Unsaturation
440.1753	39.0%	$C_{27}H_{24}N_2O_4$	440.1736	3.8	17.0

Figure S44. HRMS (EI) spectra of compound **10b**.

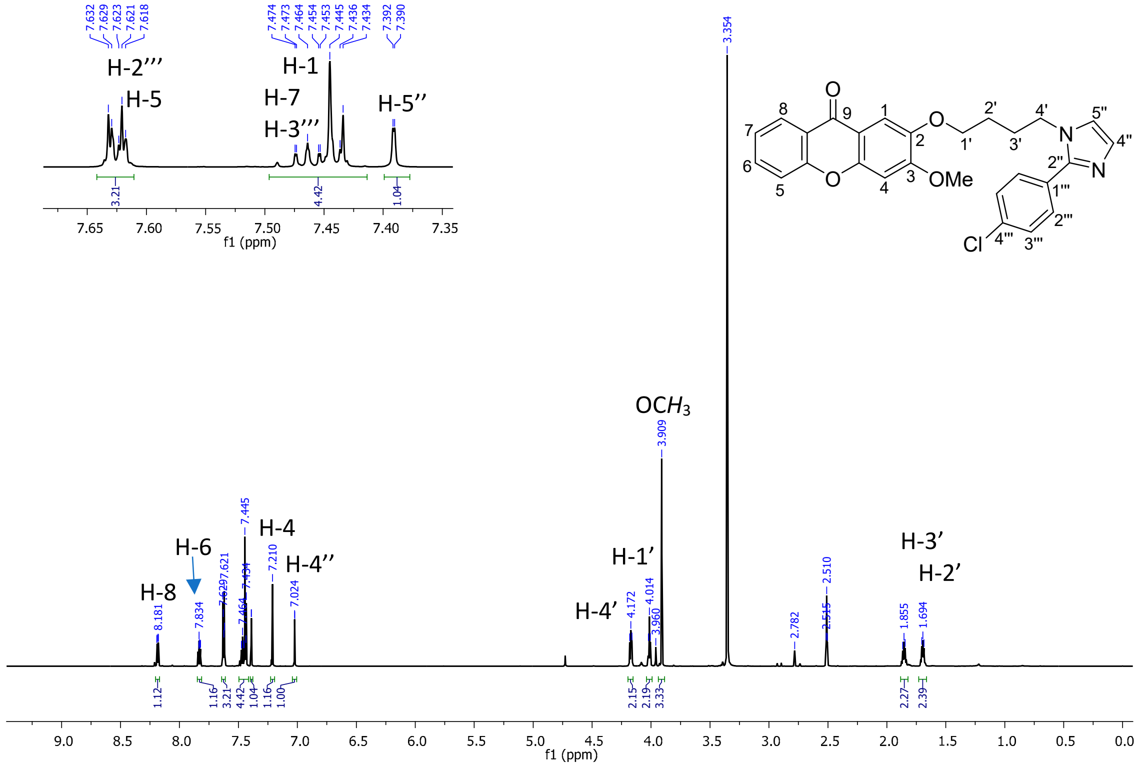

Figure S45. ^{1}H NMR (750 MHz, DMSO-d_6) spectrum of **10c**.

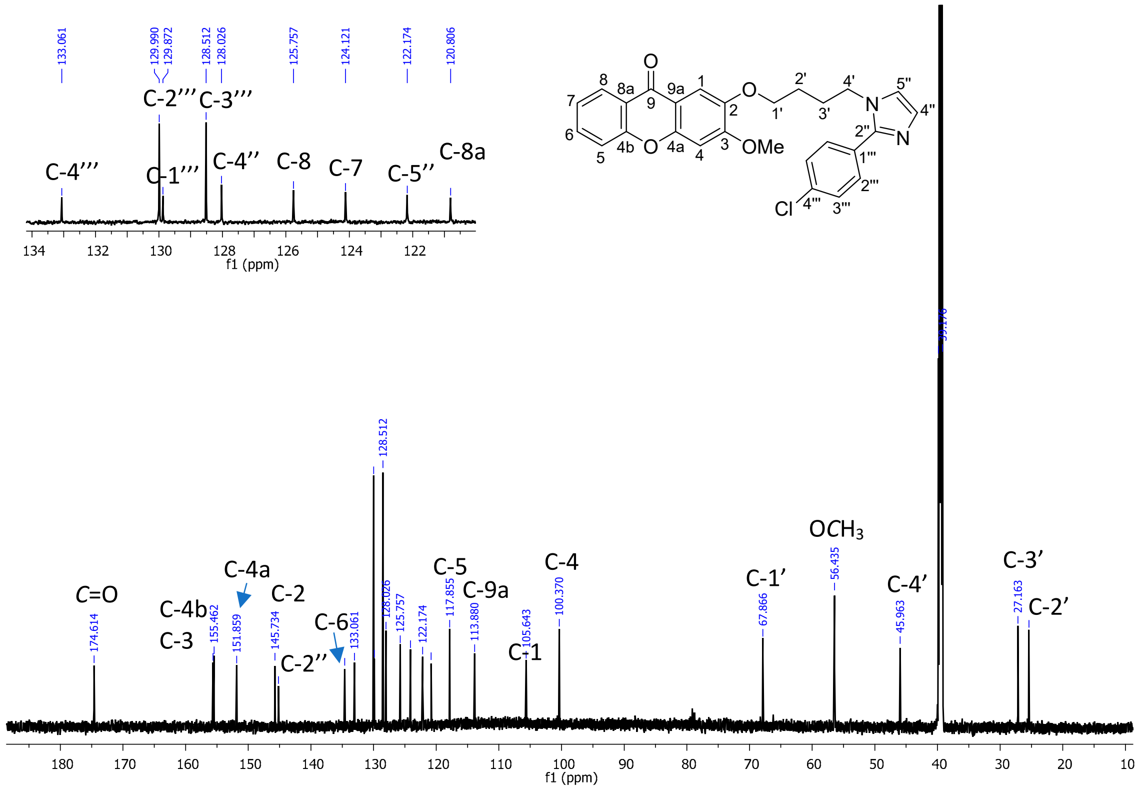

Figure S46. ^{13}C NMR (187.5 MHz, DMSO-d_6) spectrum of **10c**

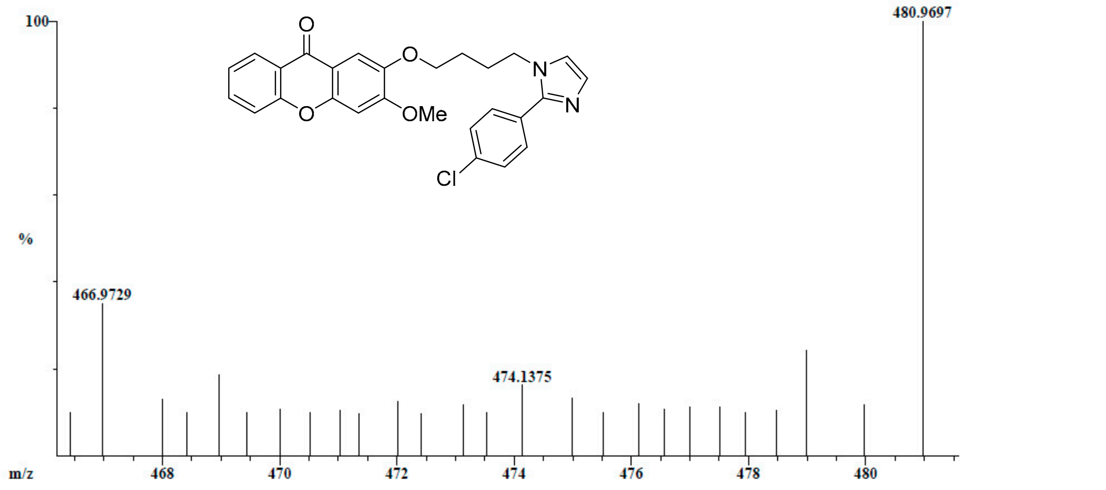

Selected Isotopes : $H_{0-23}C_{0-27}N_{0-2}O_{0-4}Cl_{0-1}$ Error Limit : 10 ppm Unsaturation Limits : 0 to 50

Measured Mass	% Base	Formula	Calculated Mass	Error	Unsaturation
474.1375	16.2%	$C_{27}H_{23}N_2O_4Cl$	474.1346	6.0	17.0

Figure S47. HRMS (EI) spectra of compound **10c**.

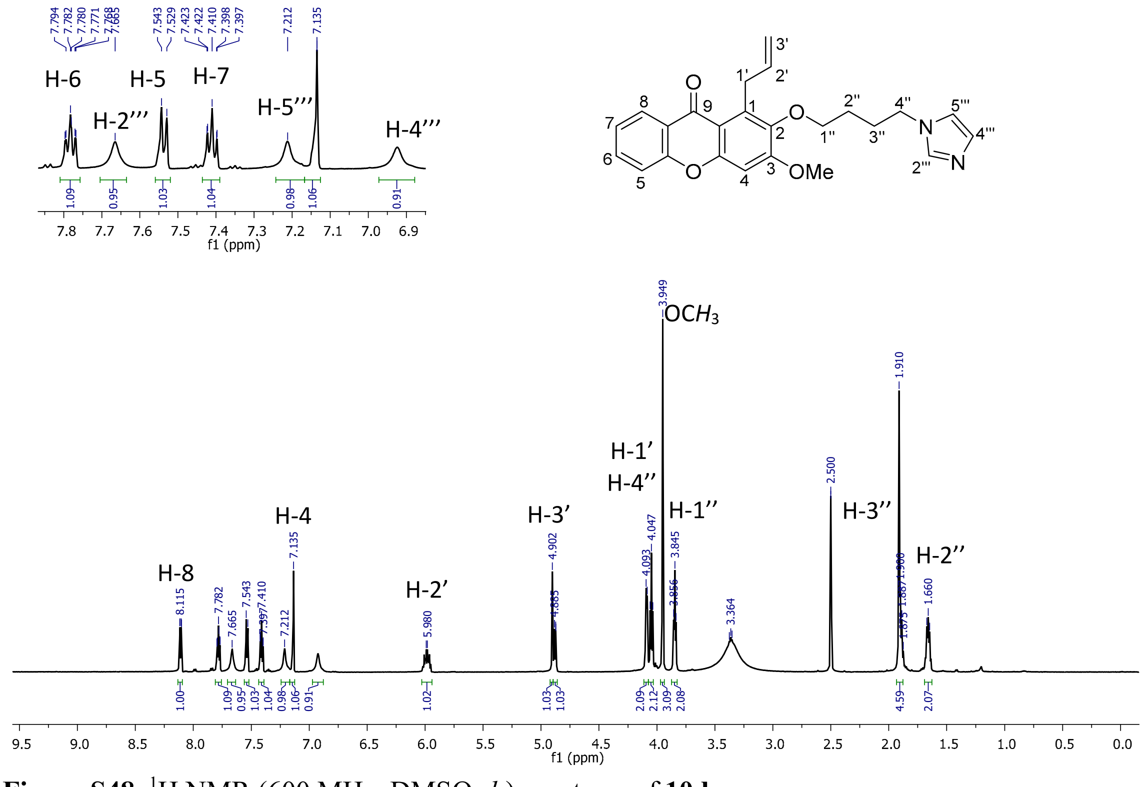

Figure S48. ^{1}H NMR (600 MHz, DMSO-d_6) spectrum of **10d**.

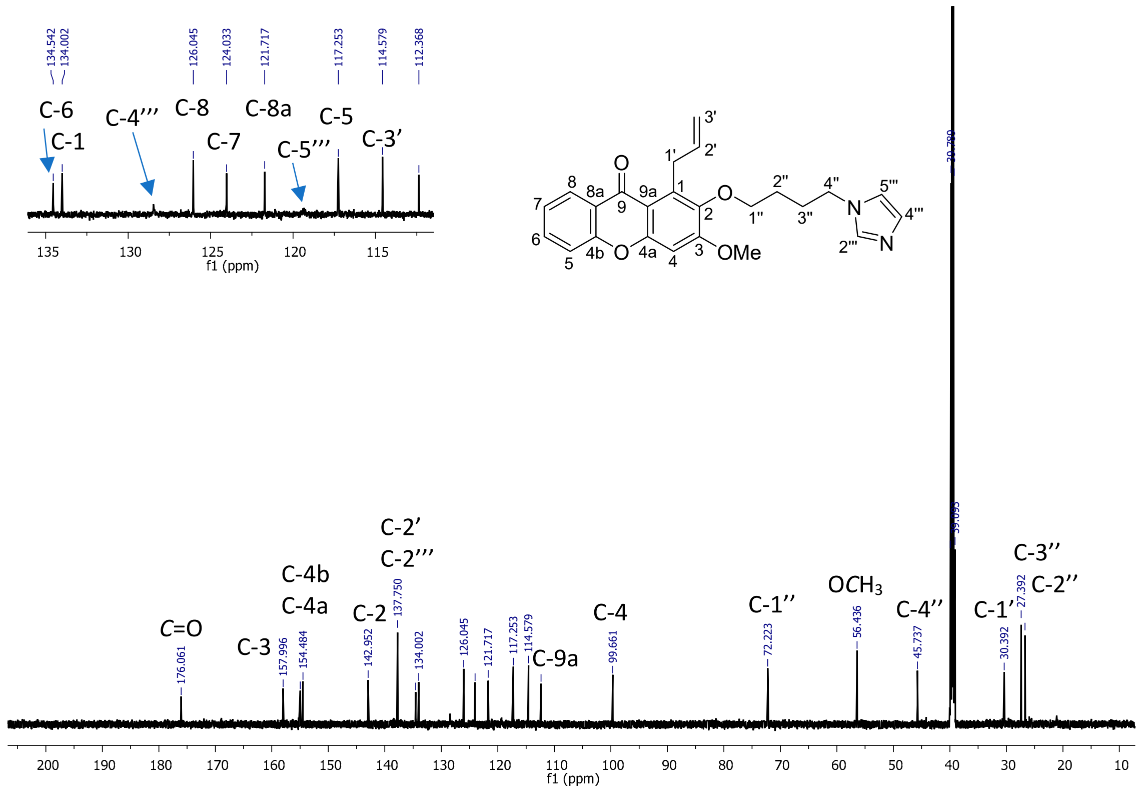

Figure S49. ^{13}C NMR (150 MHz, DMSO-d_6) spectrum of **10d**.

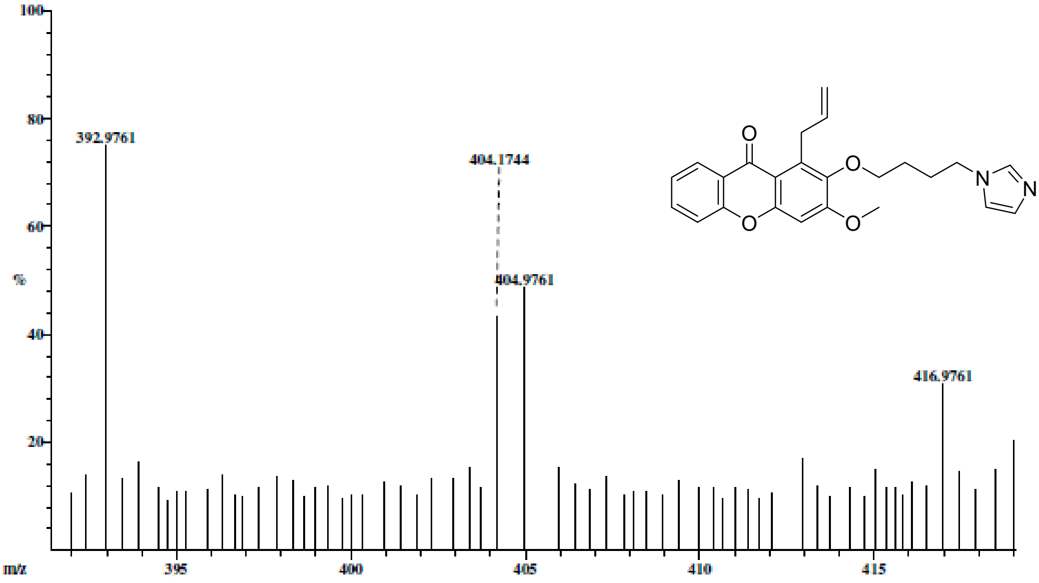

Selected Isotopes : $H_{0-24}C_{0-24}N_{0-2}O_{0-4}$ **Error Limit : 5 ppm**

Measured Mass	% Base	Formula	Calculated Mass	Error
404.1744	43.2%	$C_{24}H_{24}N_2O_4$	404.1736	2.0

Figure S50. HRMS (EI) spectra of compound **10d**.

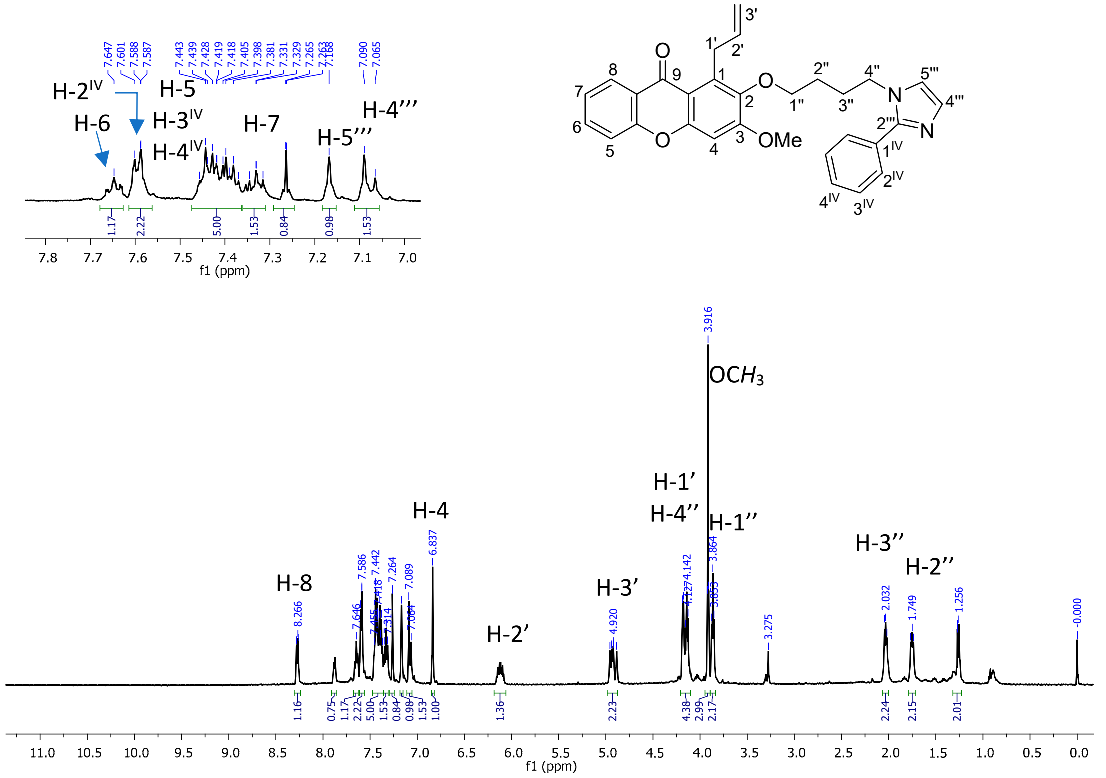

Figure S51 ^{1}H NMR (500 MHz, $CDCl_3$) spectrum of **10e**.

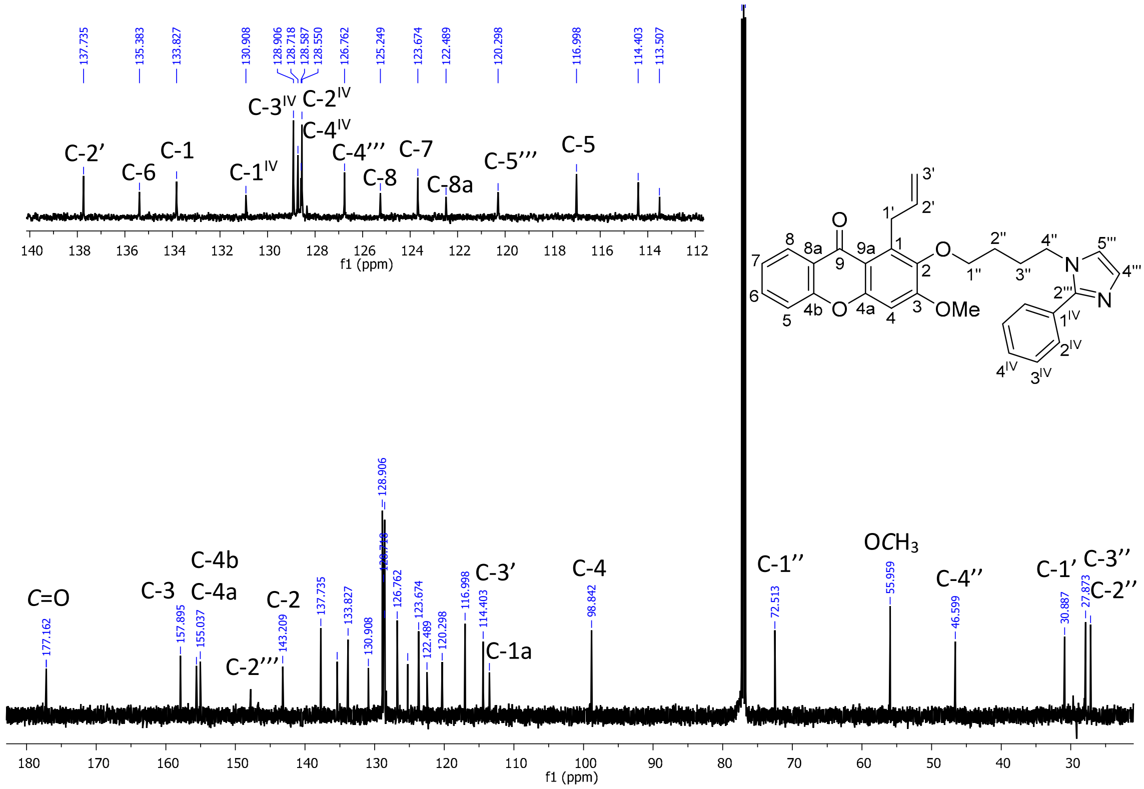

Figure S52. ^{13}C NMR (125 MHz, $CDCl_3$) spectrum of **10e**.

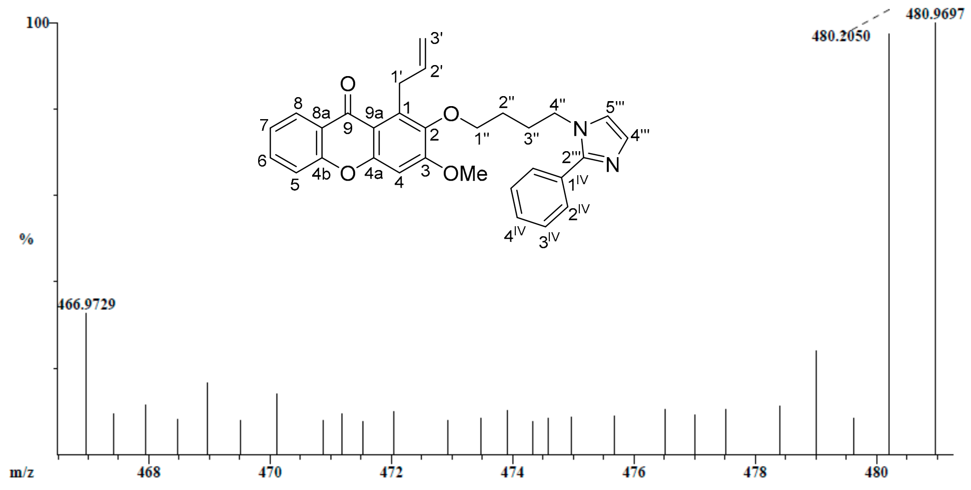

Selected Isotopes : $H_{0-28}C_{0-30}N_{0-2}O_{0-4}$ Error Limit : 5 ppm Unsaturation Limits : 0 to 50

Measured Mass	% Base	Formula	Calculated Mass	Error	Unsaturation
480.2050	97.4%	$C_{30}H_{28}N_2O_4$	480.2049	0.2	18.0

Figure S53. HRMS (EI) spectra of compound **10e**.

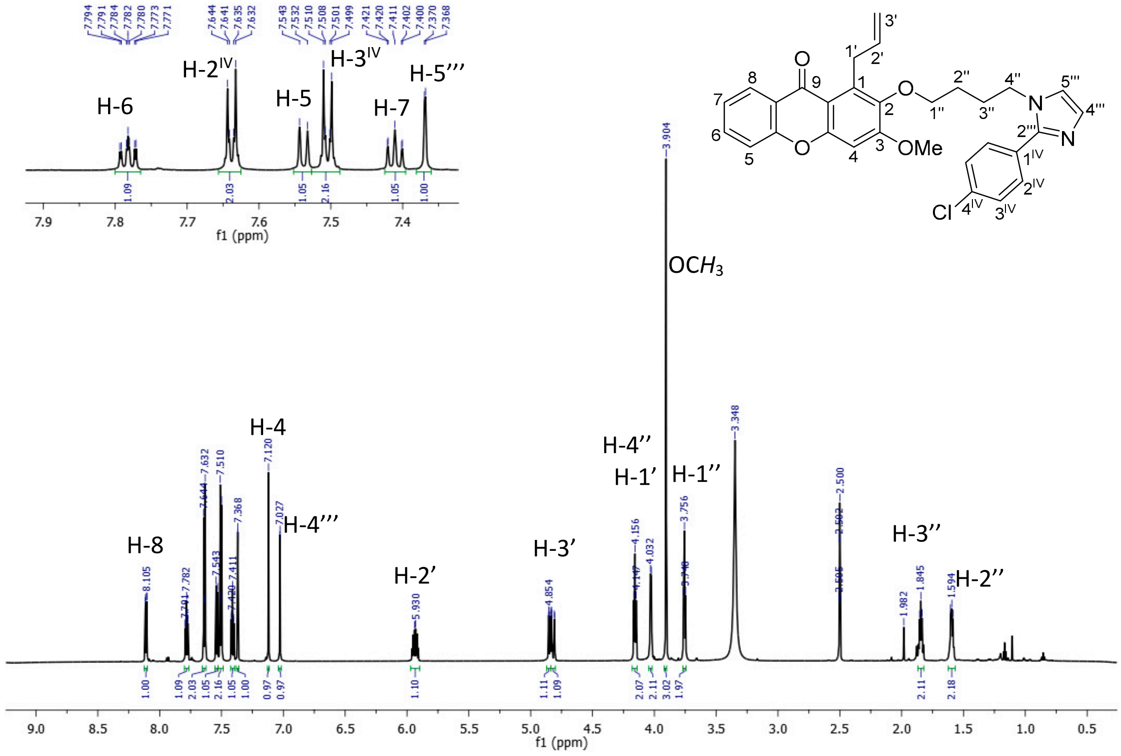

Figure S54. ^{1}H NMR (750 MHz, DMSO-d_6) spectrum of **10f**.

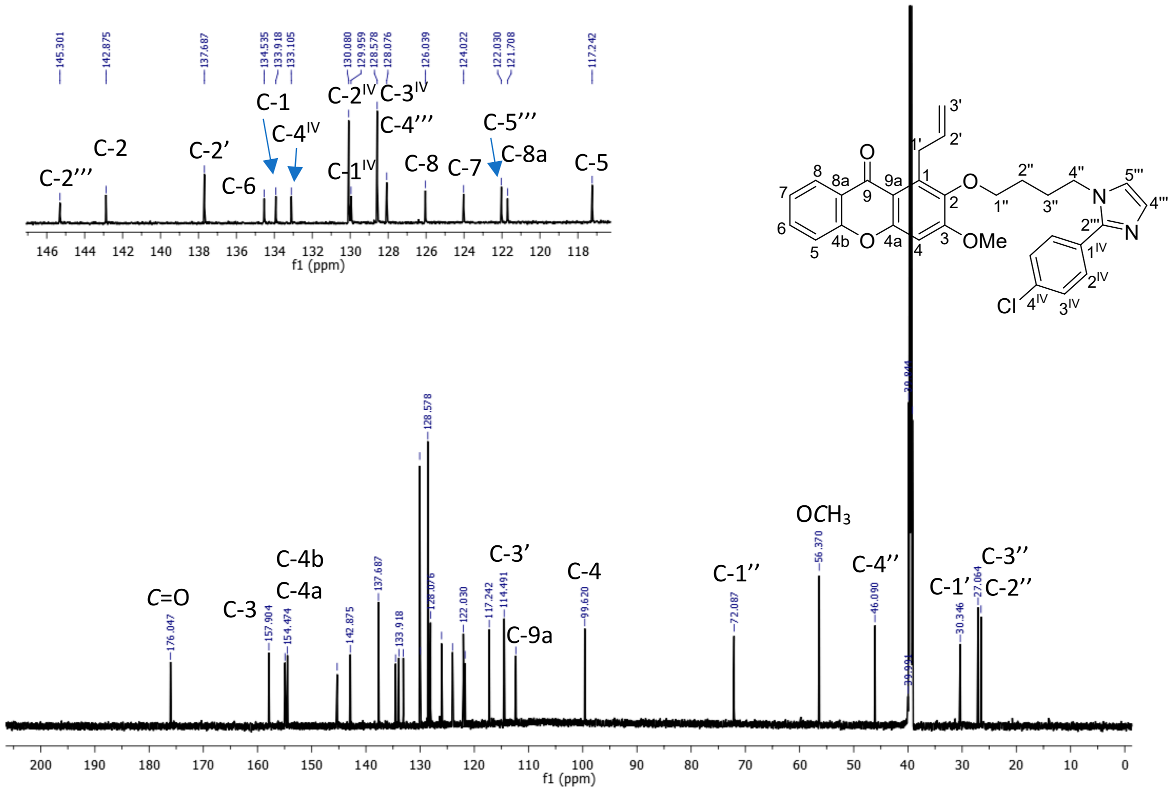

Figure S55. ^{13}C NMR (187.5 MHz, DMSO-d_6) spectrum of **10f**.

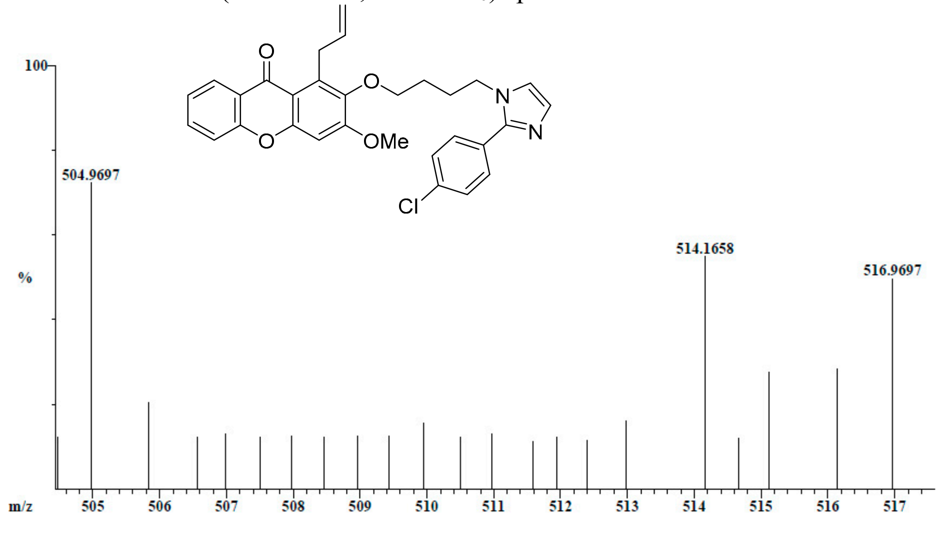

Selected Isotopes : $H_{0-27}C_{0-30}N_{0-2}O_{0-4}Cl_{0-1}$ Error Limit : 5 ppm Unsaturation Limits : 0 to 50

Measured Mass	% Base	Formula	Calculated Mass	Error	Unsaturation
514.1658	54.8%	$C_{30}H_{27}N_2O_4Cl$	514.1659	-0.3	18.0

Figure S56. HRMS (EI) spectra of compound **10f**.

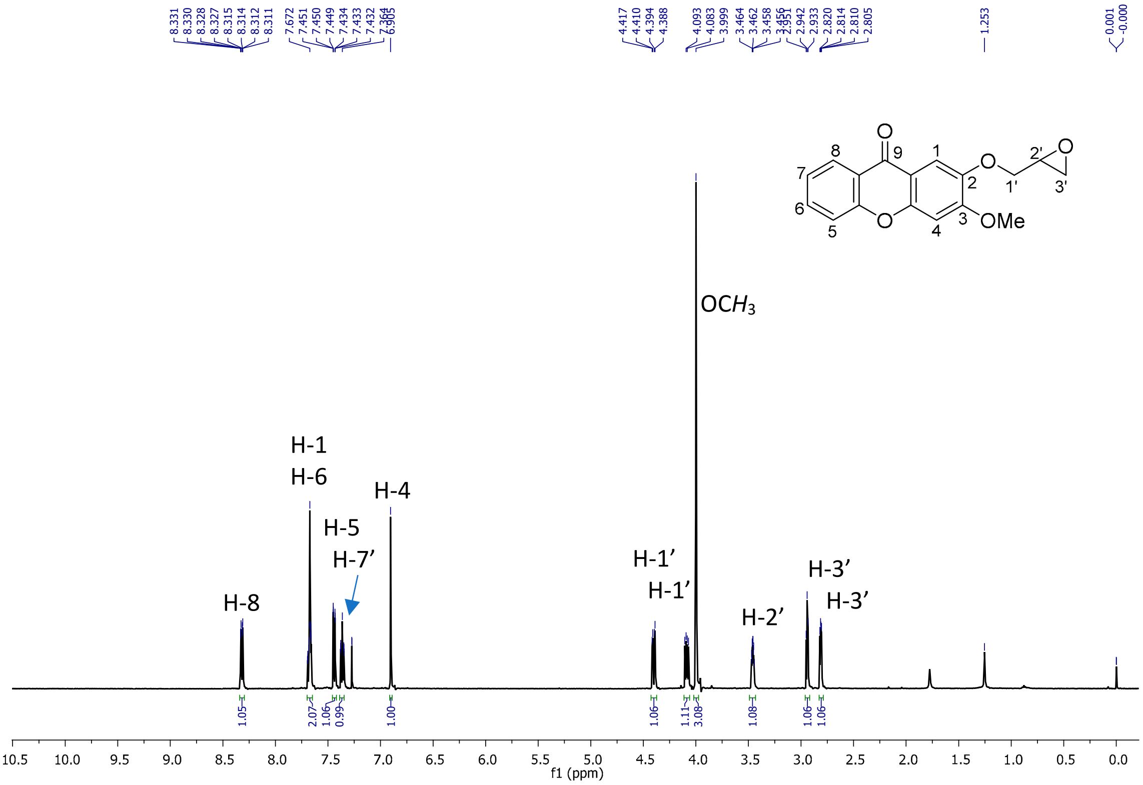

Figure S57. ^{1}H NMR (500 MHz, $CDCl_3$) spectrum of **11a**.

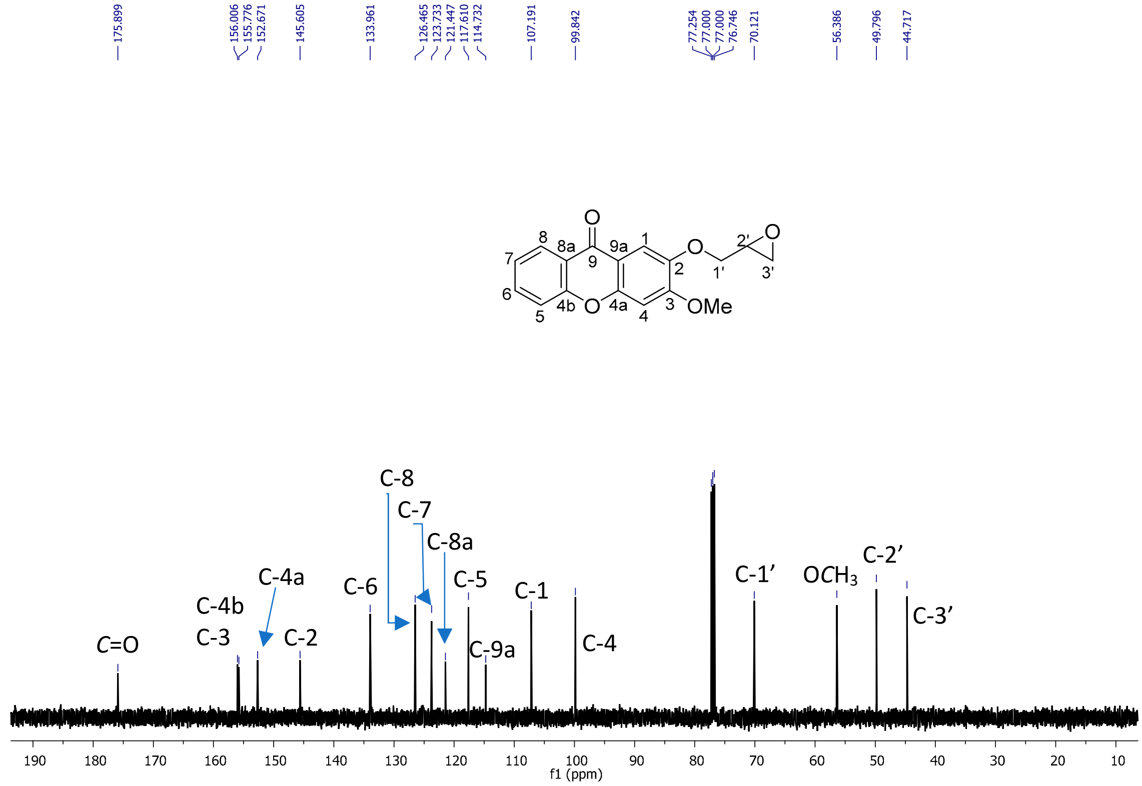

Figure S58. ^{13}C NMR (125 MHz, $CDCl_3$) spectrum of **11a**.

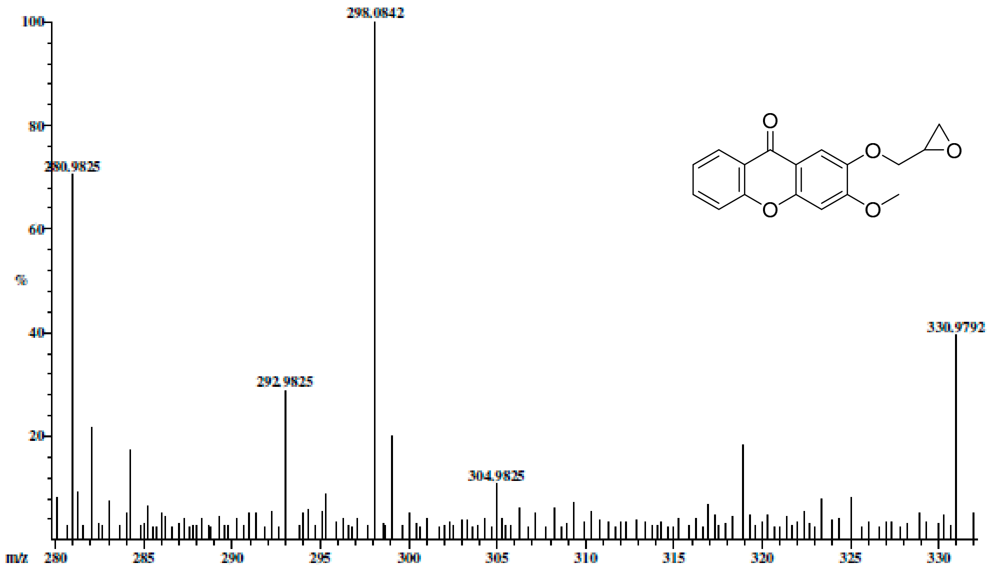

Selected Isotopes : $H_{0-14}C_{0-17}O_{0-5}$ Error Limit : 5 ppm

Measured Mass	% Base	Formula	Calculated Mass	Error
298.0842	100.0%	$C_{17}H_{14}O_5$	298.0841	0.3

Figure S59. HRMS (EI) spectra of compound **11a**.

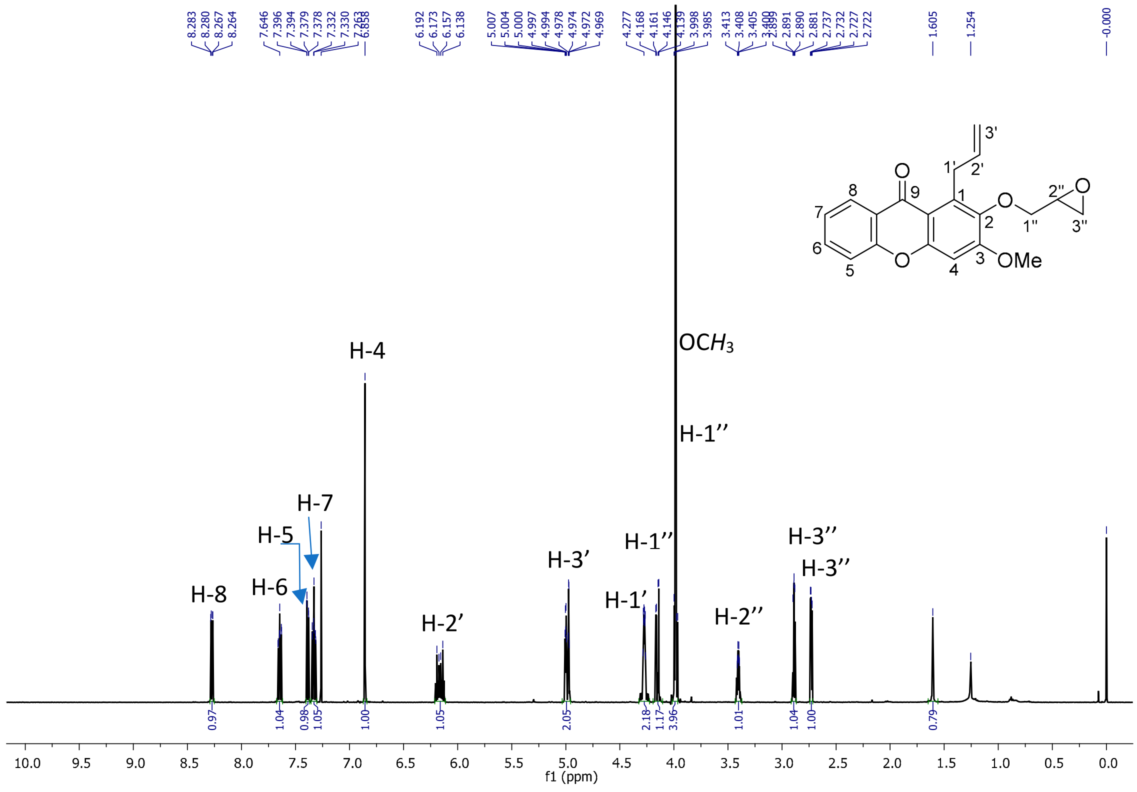

Figure S60. ^{1}H NMR (500 MHz, $CDCl_3$) spectrum of **11b**.

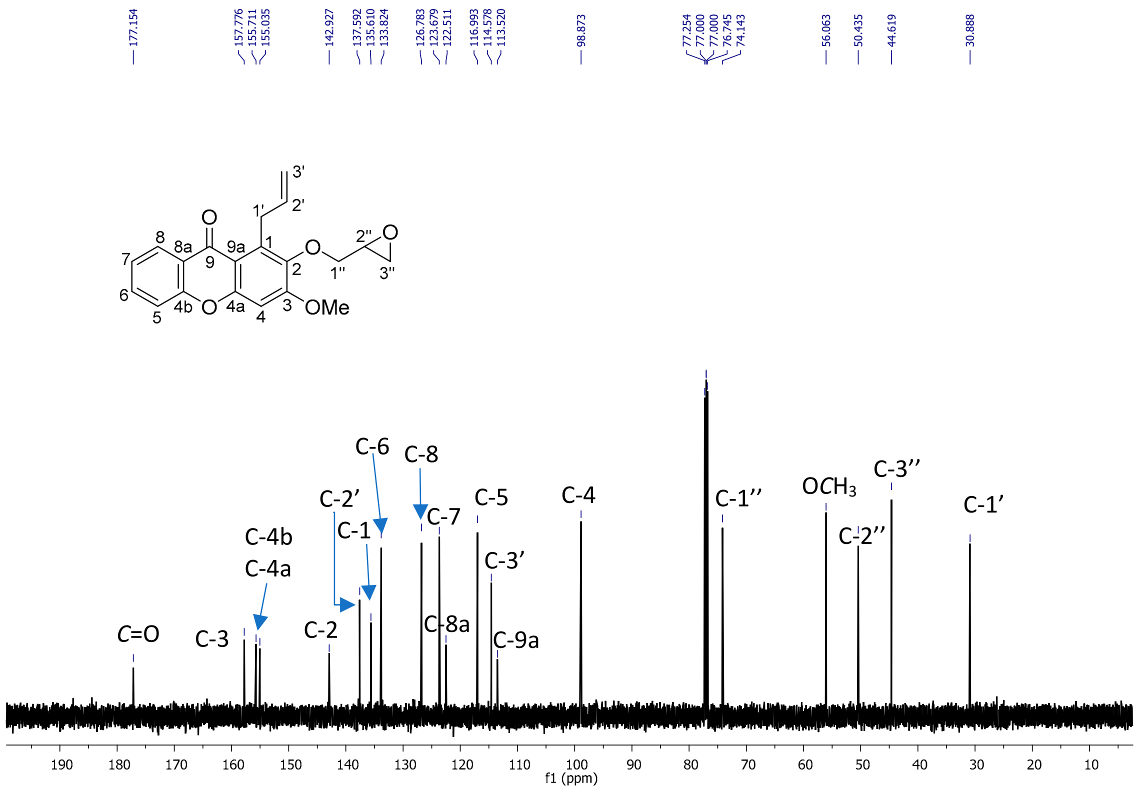

Figure S61. ^{13}C NMR (125 MHz, $CDCl_3$) spectrum of **11b**.

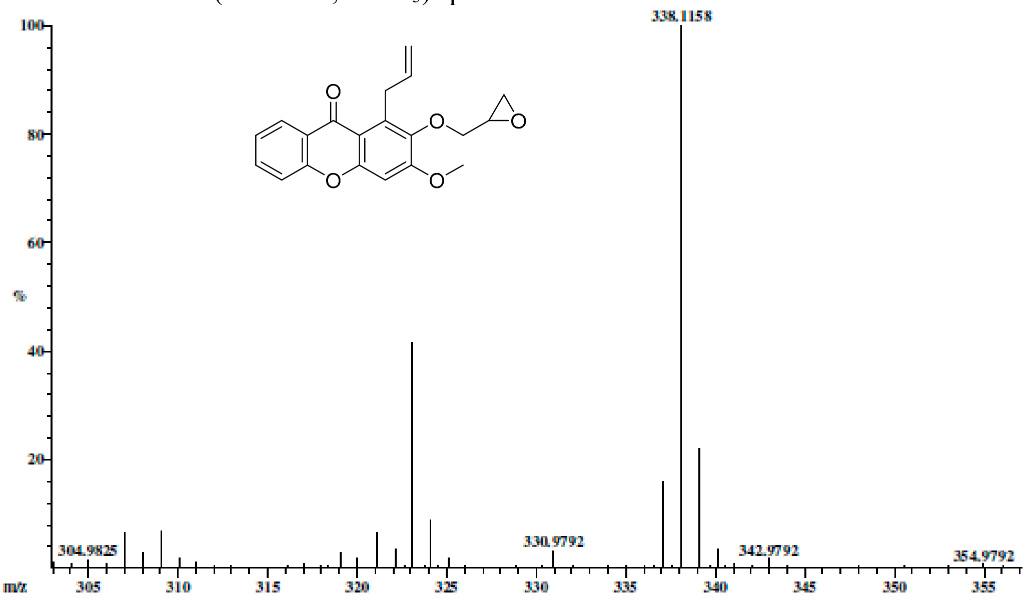

Selected Isotopes : $H_{0-18}C_{0-20}O_{0-5}$

Error Limit : 5 ppm

Measured Mass	*% Base*	*Formula*	*Calculated Mass*	*Error*
338.1158	100.0%	$C_{20}H_{18}O_5$	338.1154	1.1

Figure S62. HRMS (EI) spectra of compound **11b**.

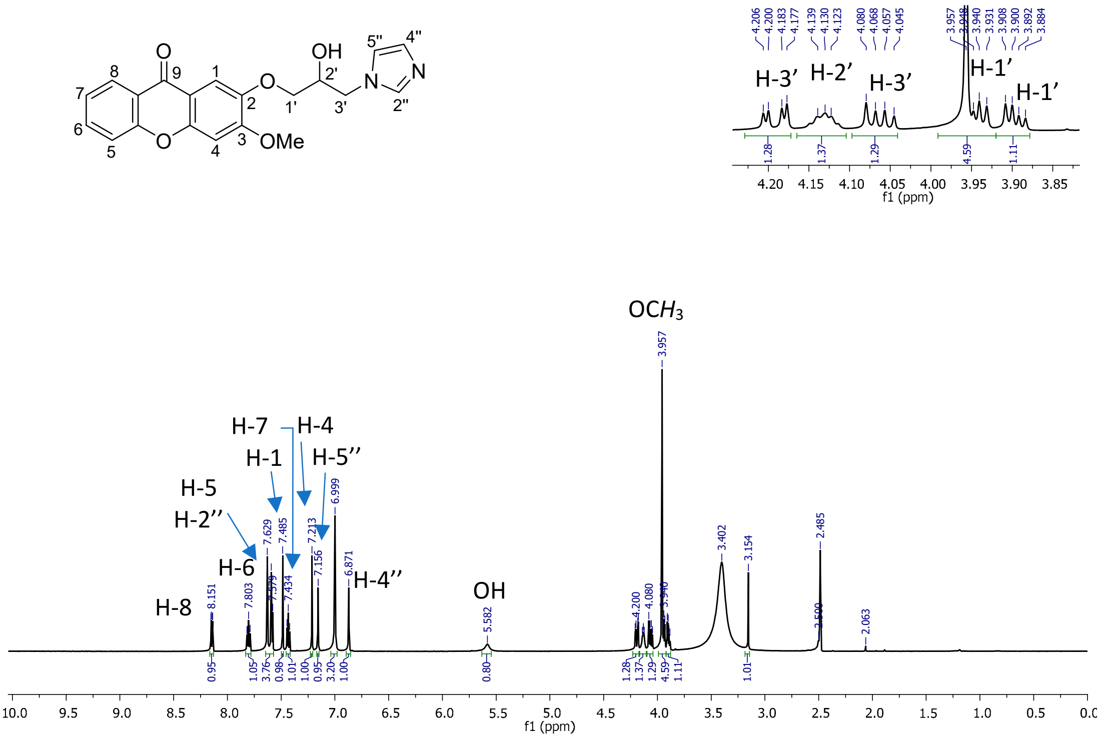

Figure S63. ^{1}H NMR (600 MHz, DMSO-d_6) spectrum of **12a**.

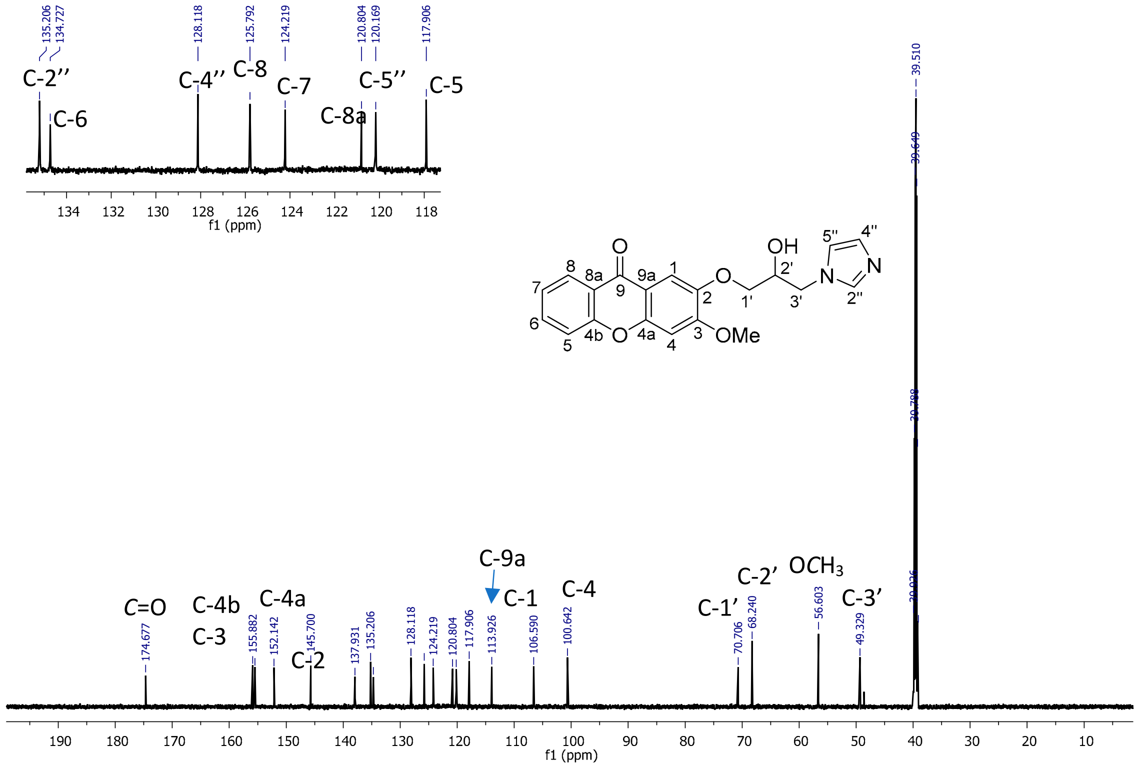

Figure S64. ^{13}C NMR (150 MHz, DMSO-d_6) spectrum of **12a.**

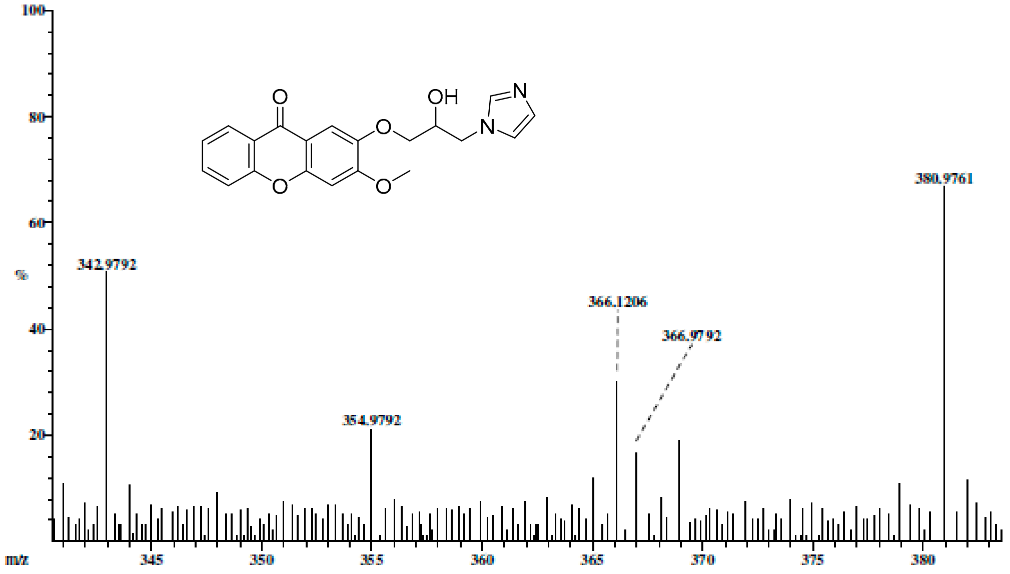

Selected Isotopes : $H_{0-18}C_{0-20}N_{0-2}O_{0-5}$ Error Limit : 5 ppm

Measured Mass	% Base	Formula	Calculated Mass	Error
366.1206	30.1%	$C_{20}H_{18}N_2O_5$	366.1216	-2.7

Figure S65. HRMS (EI) spectra of compound **12a**.

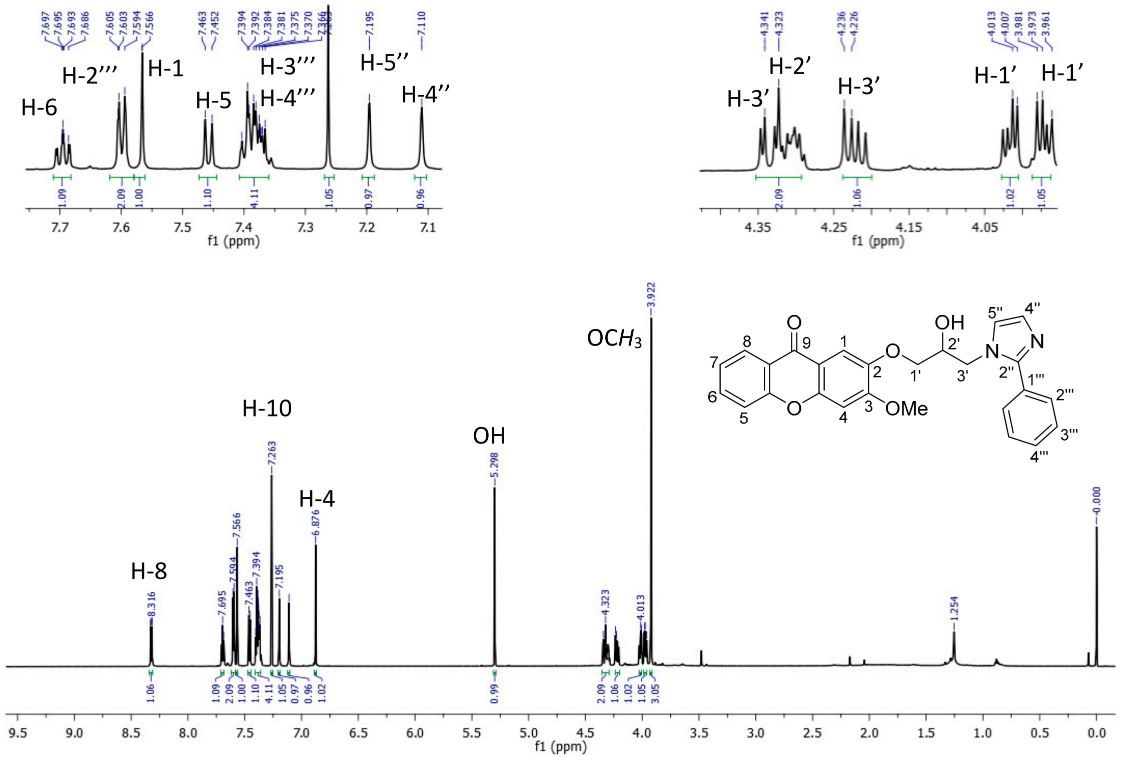

Figure S66. ^{1}H NMR (750 MHz, $CDCl_3$) spectrum of **12b**.

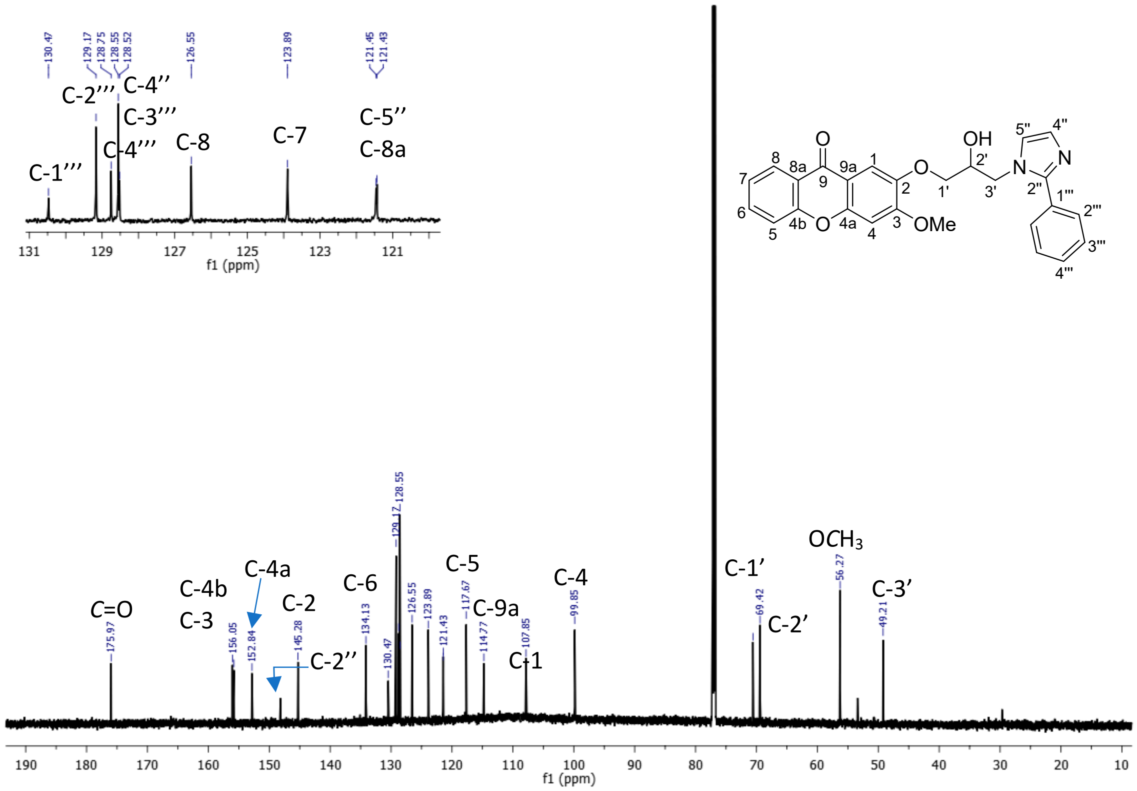

Figure S67. ^{13}C NMR (187.5 MHz, $CDCl_3$) spectrum of **12b**

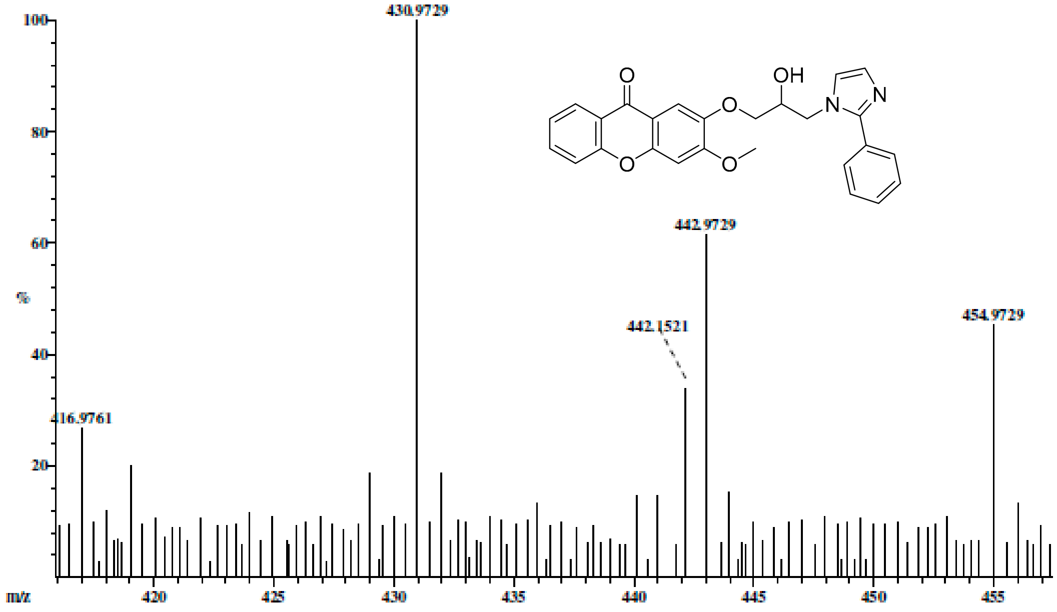

Selected Isotopes : $H_{0-22}C_{0-26}N_{0-2}O_{0-5}$ Error Limit : 5 ppm

Measured Mass	% Base	Formula	Calculated Mass	Error
442.1521	33.6%	$C_{26}H_{22}N_2O_5$	442.1529	-1.7

Figure S68. HRMS (EI) spectra of compound **12b**.

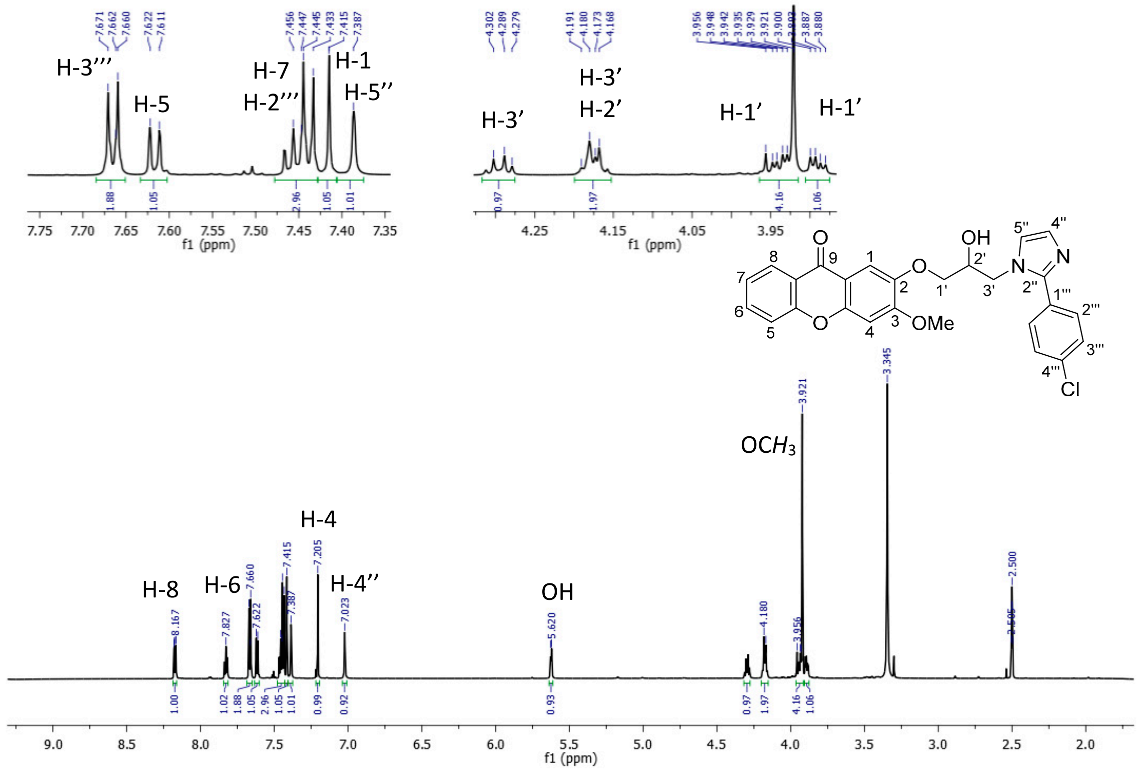

Figure S69. ^{1}H NMR (750 MHz, DMSO-d_6) spectrum of **12c**.

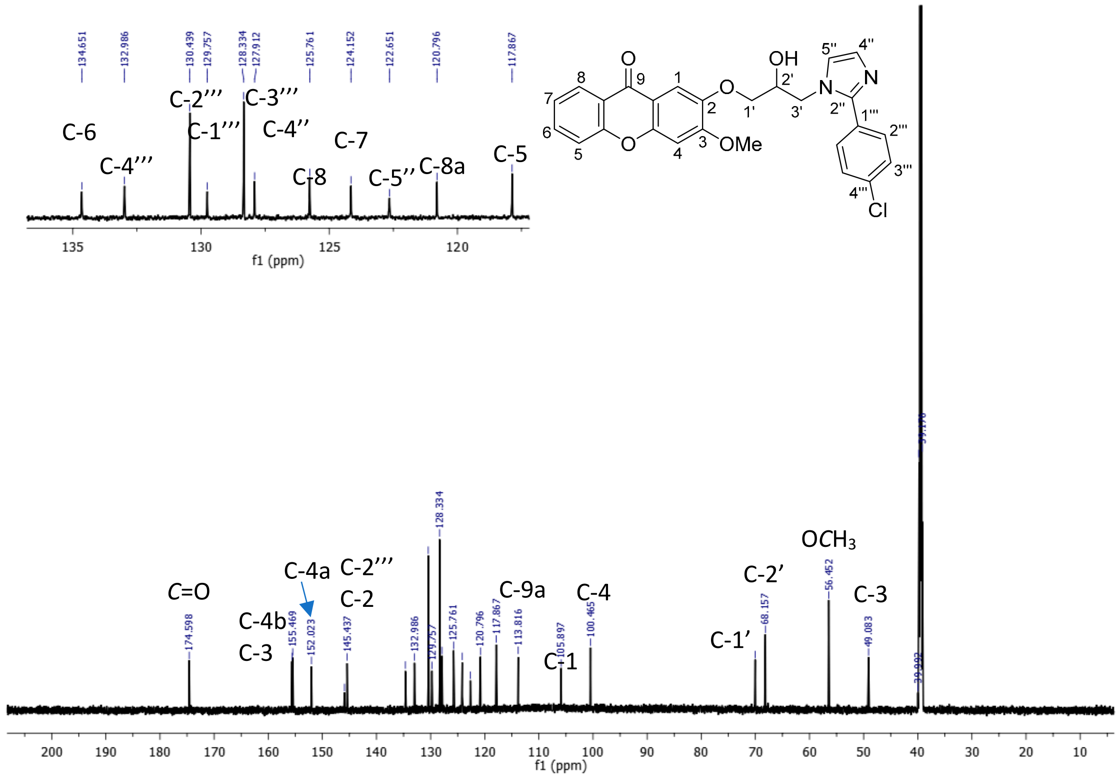

Figure S70. ^{13}C NMR (187.5 MHz, DMSO-d_6) spectrum of **12c**.

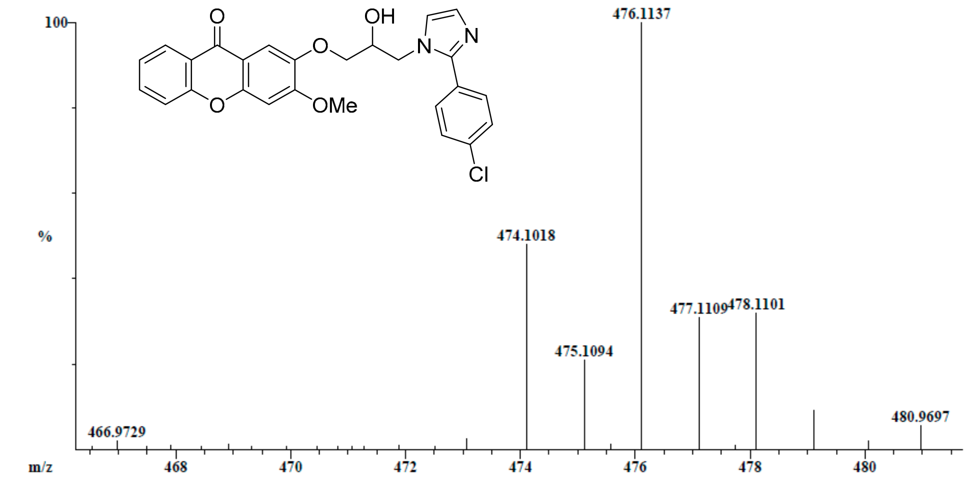

Selected Isotopes : $H_{0\text{-}21}C_{0\text{-}26}N_{0\text{-}2}O_{0\text{-}5}Cl_{0\text{-}1}$ Error Limit : 5 ppm Unsaturation Limits : 0 to 50

Measured Mass	% Base	Formula	Calculated Mass	Error	Unsaturation
476.1137	100.0%	$C_{26}H_{21}N_2O_5Cl$	476.1139	-0.4	17.0

Figure S71. HRMS (EI) spectra of compound **12c**.

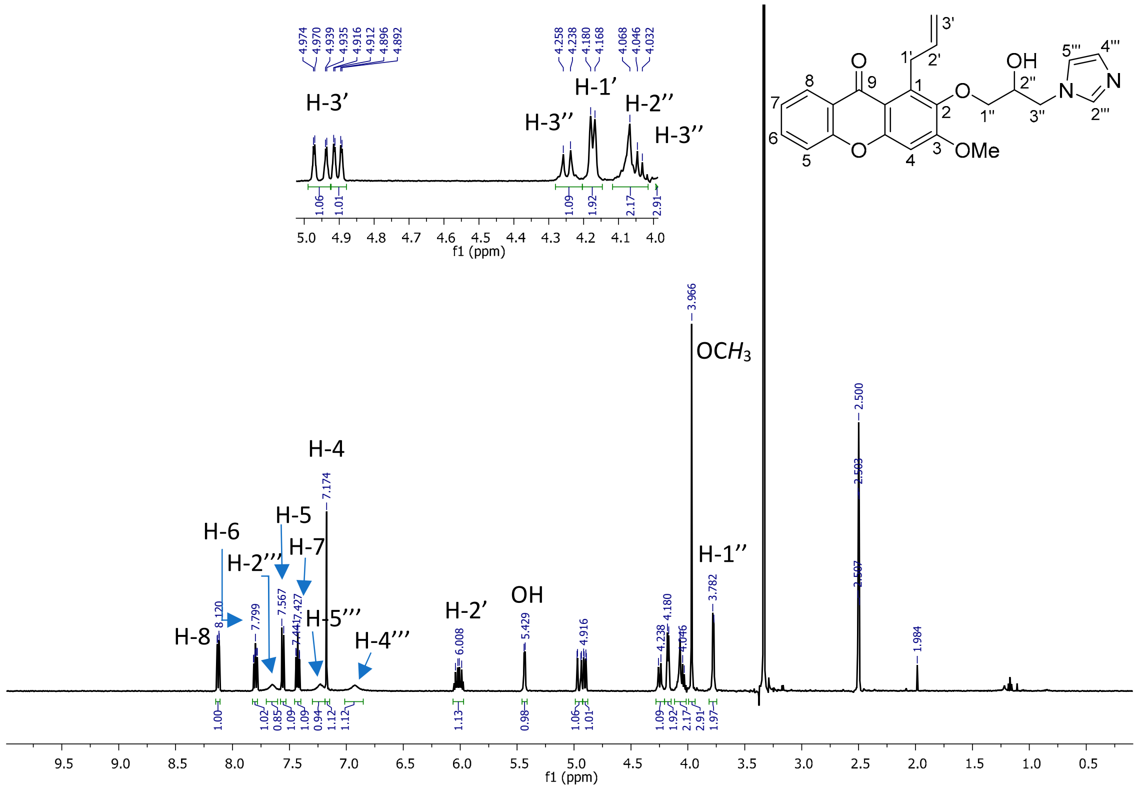

Figure S72. ^{1}H NMR (500 MHz, DMSO-d_6) spectrum of **12d**.

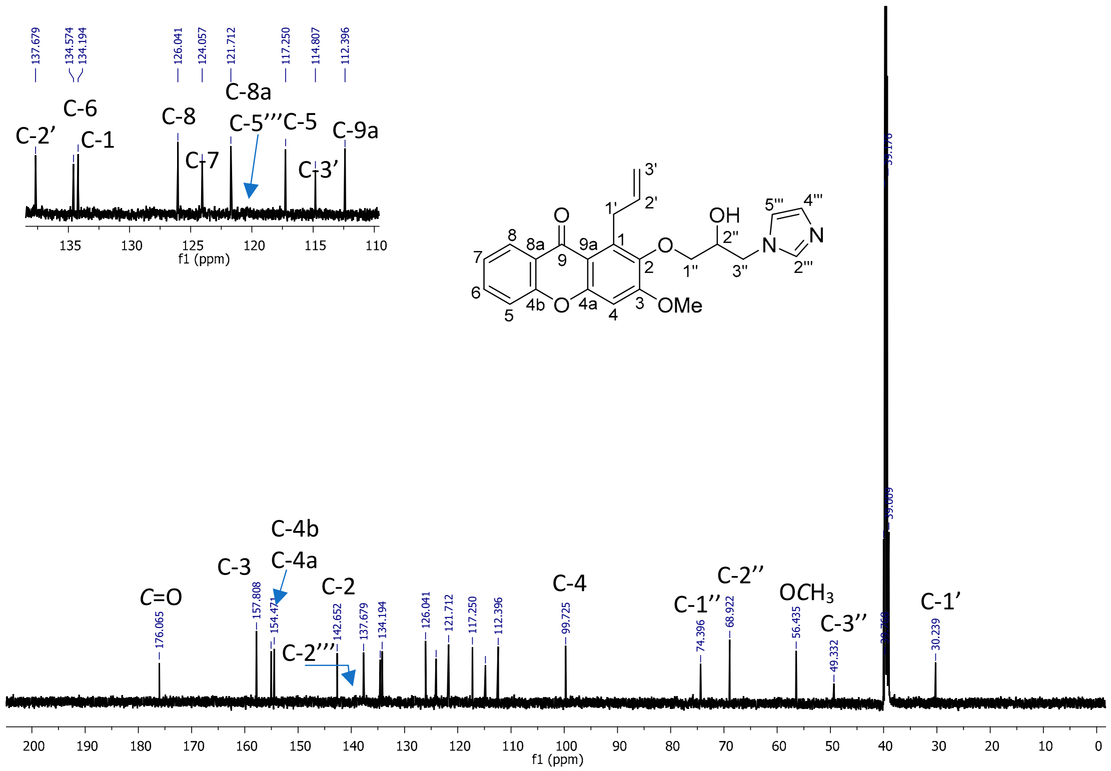

Figure S73. ^{13}C NMR (125 MHz, DMSO-d_6) spectrum of **12d**.

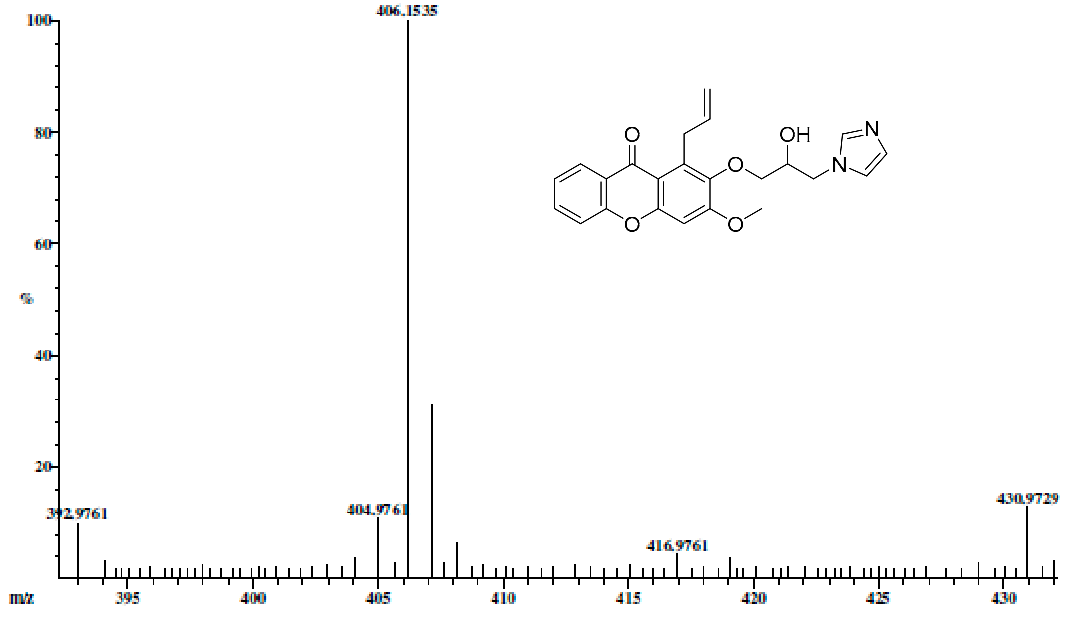

Selected Isotopes : $H_{0-22}C_{0-23}N_{0-2}O_{0-5}$

Error Limit : 5 ppm

Measured Mass	% Base	Formula	Calculated Mass	Error
406.1535	100.0%	$C_{23}H_{22}N_2O_5$	406.1529	1.5

Figure S74. HRMS (EI) spectra of compound **12d**.

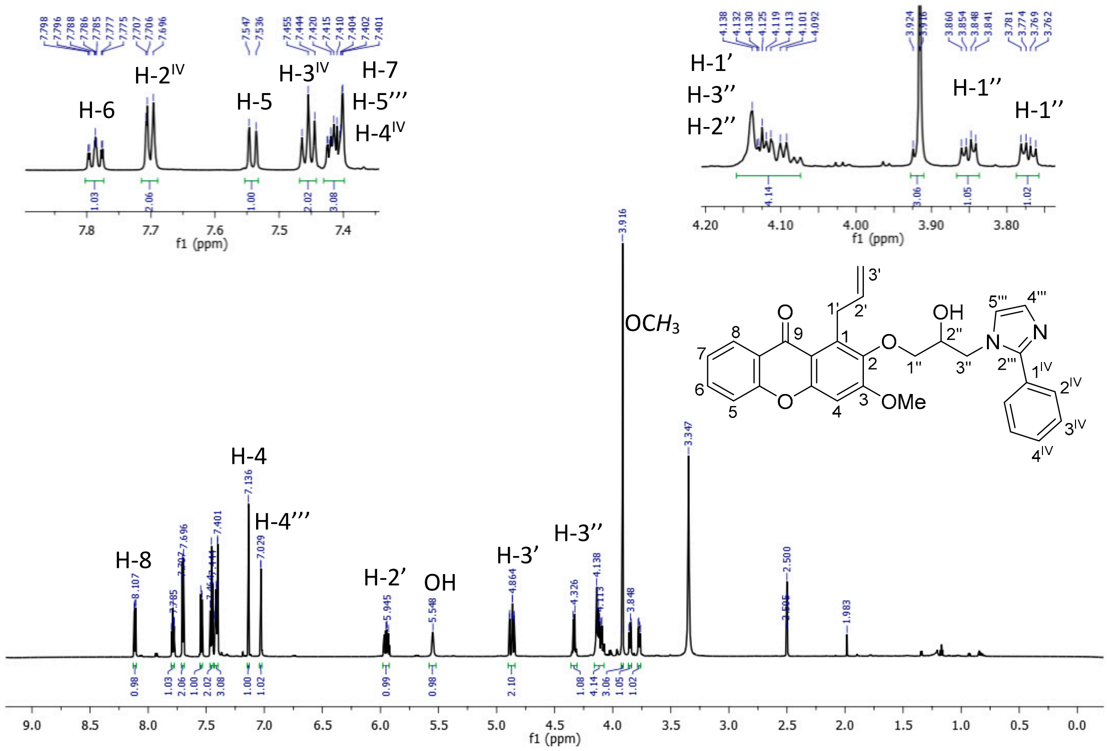

Figure S75. ^{1}H NMR (750 MHz, DMSO-d_6) spectrum of **12e**.

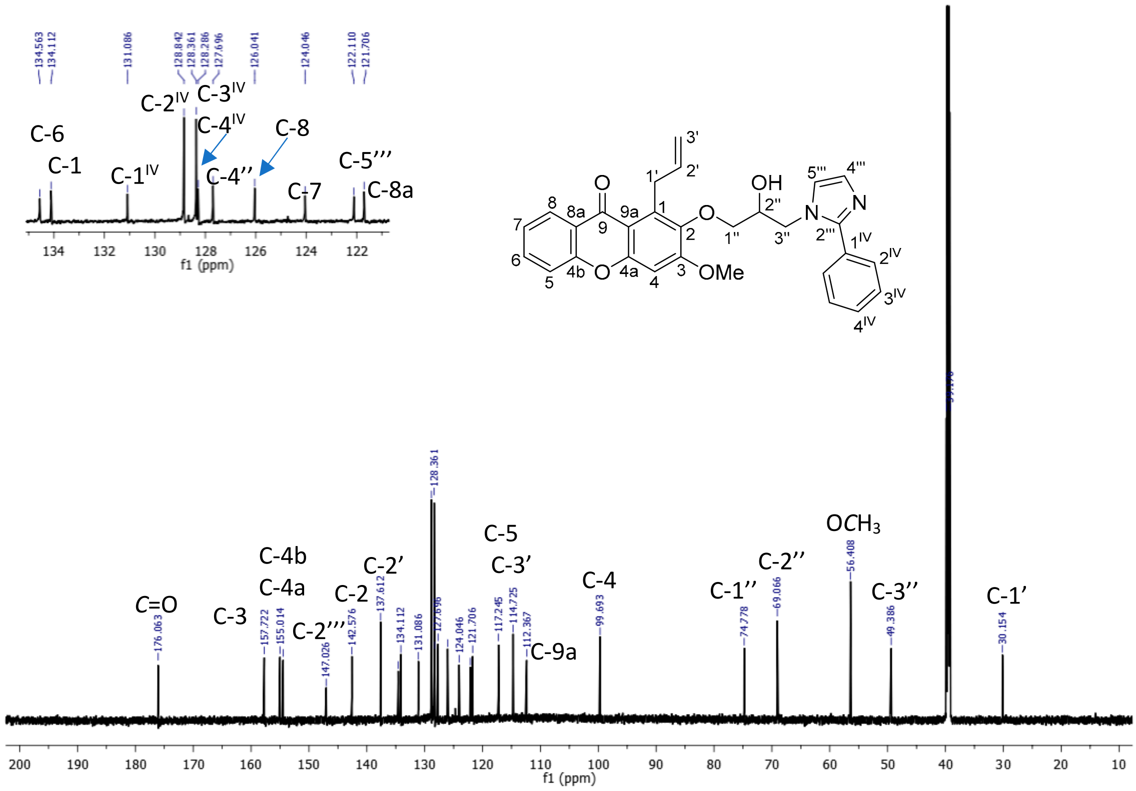

Figure S76. ^{13}C NMR (187.5 MHz, DMSO-d_6) spectrum of **12e**

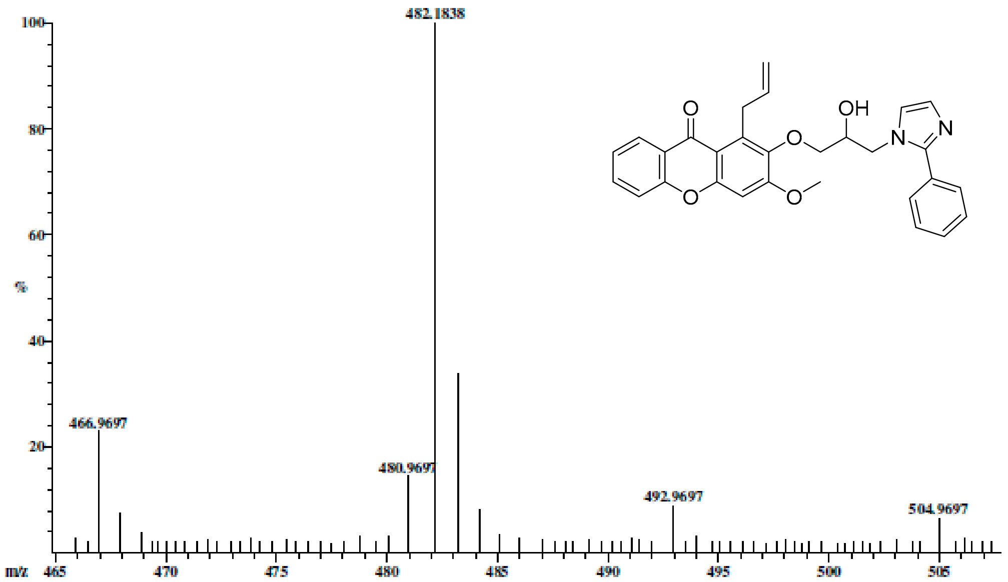

Selected Isotopes : $H_{0-26}C_{0-29}N_{0-2}O_{0-5}$ Error Limit : 5 ppm

Measured Mass	% Base	Formula	Calculated Mass	Error
482.1838	100.0%	$C_{29}H_{26}N_2O_5$	482.1842	-0.8

Figure S77. HRMS (EI) spectra of compound **12e**.

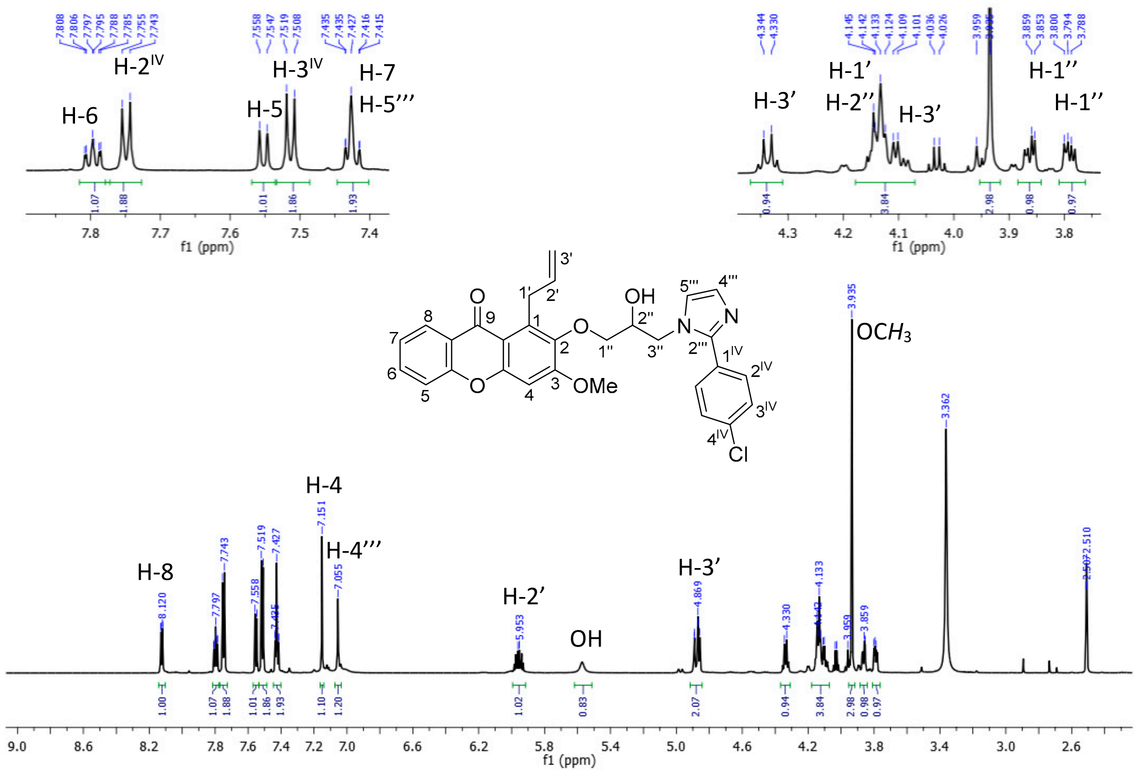

Figure S78. ^{1}H NMR (750 MHz, DMSO-d_6) spectrum of **12f**.

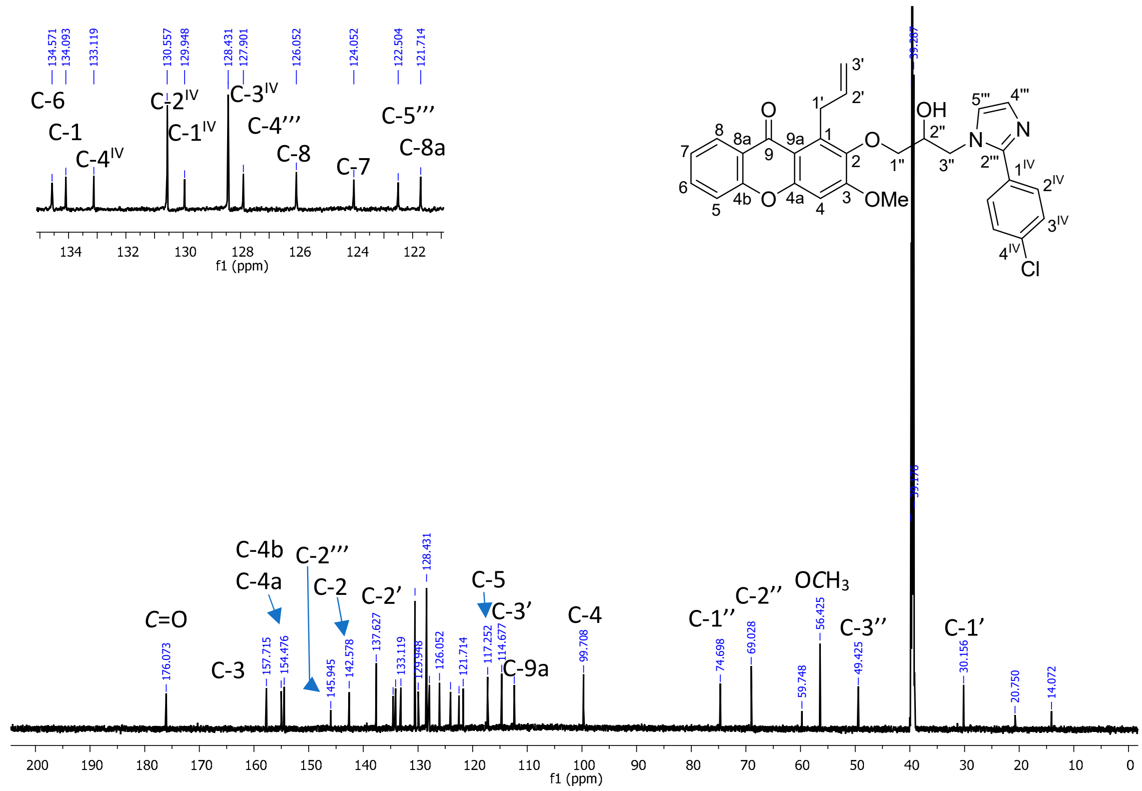

Figure S79. ^{13}C NMR (187.5 MHz, DMSO-d_6) spectrum of **12f**.

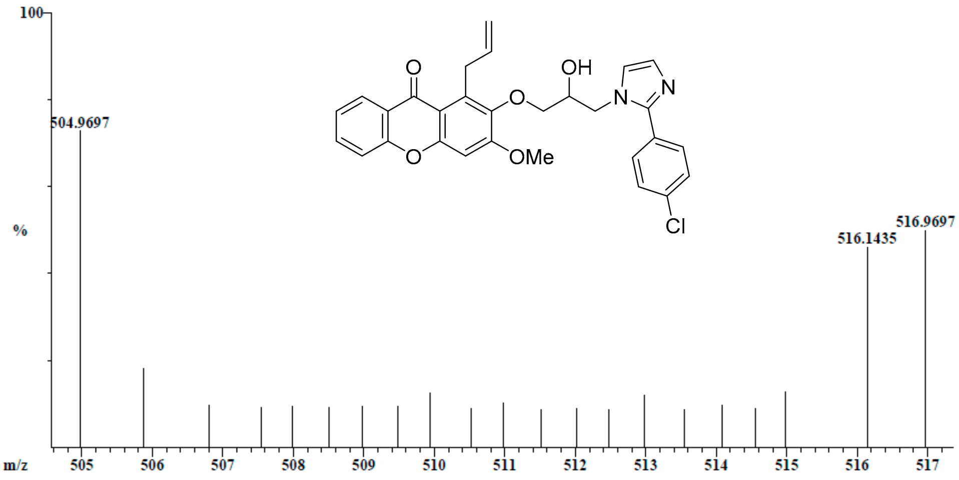

Selected Isotopes : $H_{0-25}C_{0-29}N_{0-2}O_{0-5}Cl_{0-1}$ Error Limit : 5 ppm Unsaturation Limits : 0 to 50

Measured Mass	% Base	Formula	Calculated Mass	Error	Unsaturation
516.1435	46.1%	$C_{29}H_{25}N_2O_5Cl$	516.1452	-3.3	18.0

Figure S80. HRMS (EI) spectra of compound **12f**.

Table S1. Docking results of **10c** at the active site binding pocket of isomaltase.

Compound	Binding energy Δ*G* (kcal/mol)	Interaction residues	Polar interactions	Hydrophobic interactions
**10c**	-9.82	Tyr158, Phe159, Phe178, Val216, Gln279, His280, Phe303, Arg315, Tyr316, Asp352, Glu411, Arg442	C-H····O (Tyr316)<br>C-H····O (Glu411)<br>O···H-N (Arg442)	**π-alkyl-**Phe178, Val216<br>**π-cation-**Arg315<br>**π-anion-**Asp352

10c

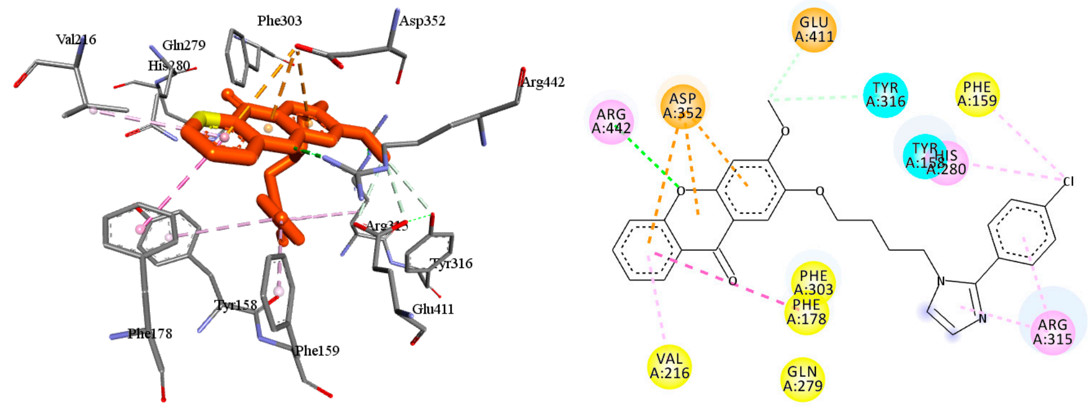

Figure 81. Molecular docking found for **10c** within the active pocket of isomaltase.

Table S2. Docking results of imidazole-substituted xanthones **6c**, **9a** at the active site binding pocket of α-amylase.

Compound	Binding energy Δ*G* (kcal/mol)	Interaction residues	Polar interactions	Hydrophobic interactions
**6c**	-7.86	Trp58, Trp59, Tyr62, His101, Leu165, Arg195, Asp197, Glu233, His299, Asp300, His305	C-H…...O (His299)	**π-π stacked-**Tyr62<br>**π-alkyl-**Leu165<br>**π-anion-**Asp300<br>**π-sigma-**His305
**9a**	-6.67	Trp58, Trp59, Tyr62, Leu165, Arg195, Asp197, Glu233, His299, Asp300, His305	C-H…...O (His299)	**π-π T-shaped-**Tyr62<br>**π-anion-**Asp300<br>**π-sigma-**His305

6c

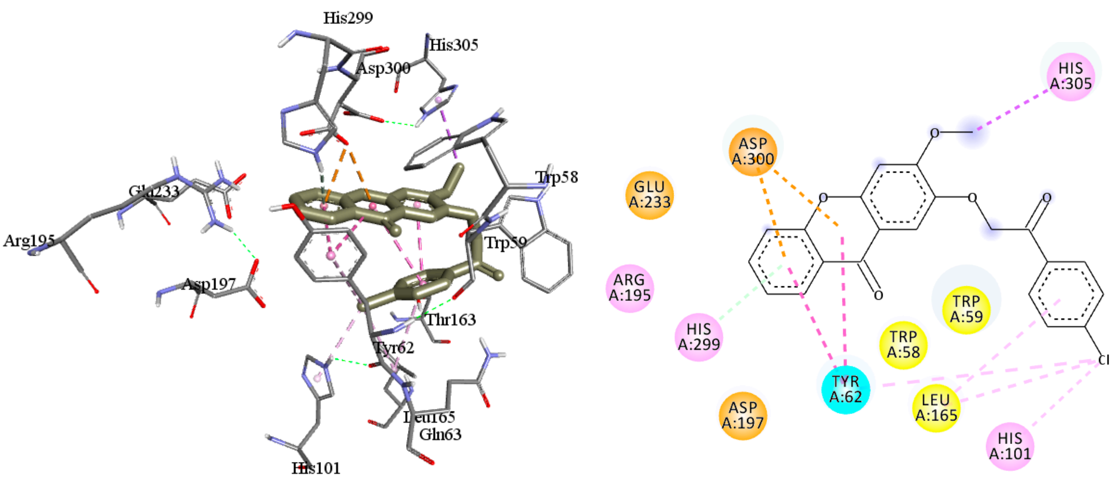

9a

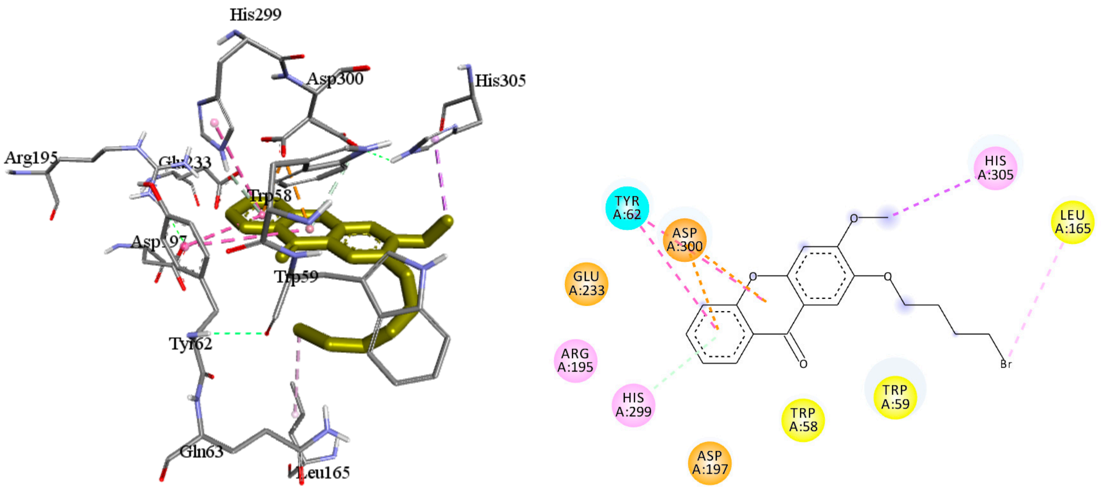

Fig. S82. Molecular docking found for **6c** and **9a** at the active site binding pocket of α-amylase.

Table S3. Calculated physicochemical properties of **1**, **6a**, **6c**, **6e**, **7**, **9a-b**, **10c**, **10f**, **11b**, **12b-f**, and (**14**).

Compound	MW (g/mol)	Log P	Log S	PSA	H-A	H-D
**14**	645.605	-7.17	0.58	321.17	19	14
**1**	242.229	2.95	-4.64	55.76	4	1
**6a**	314.292	2.78	-4.86	71.06	6	0
**6c**	394.809	4.79	-7.31	61.83	5	0
**6e**	434.874	5.82	-8.02	61.83	6	0
**7**	282.294	3.97	-5.36	55.76	4	1
**9a**	377.233	4.90	-6.34	44.76	4	0
**9b**	417.298	5.93	-7.05	44.76	4	0
**10c**	474.943	6.02	-7.79	62.58	6	0
**10f**	515.007	7.05	-8.51	62.58	6	0
**11b**	338.358	3.87	-5.82	57.29	5	0
**12b**	442.470	3.93	-6.39	82.81	7	1
**12c**	476.915	4.54	-7.13	82.81	7	1
**12d**	406.437	3.32	-4.96	82.81	7	1
**12e**	482.534	4.96	-7.10	82.81	7	1
**12f**	516.980	5.57	-7.84	82.81	7	1

MW, molecular weight (<500 g/mol); *Log P,* octanol/water partition coefficient (<5); *Log S,* aqueous solubility; *PSA,* topological polar surface area; *HA,* hydrogen bond acceptor (<10); *HD,* hydrogen bond donor (<5)

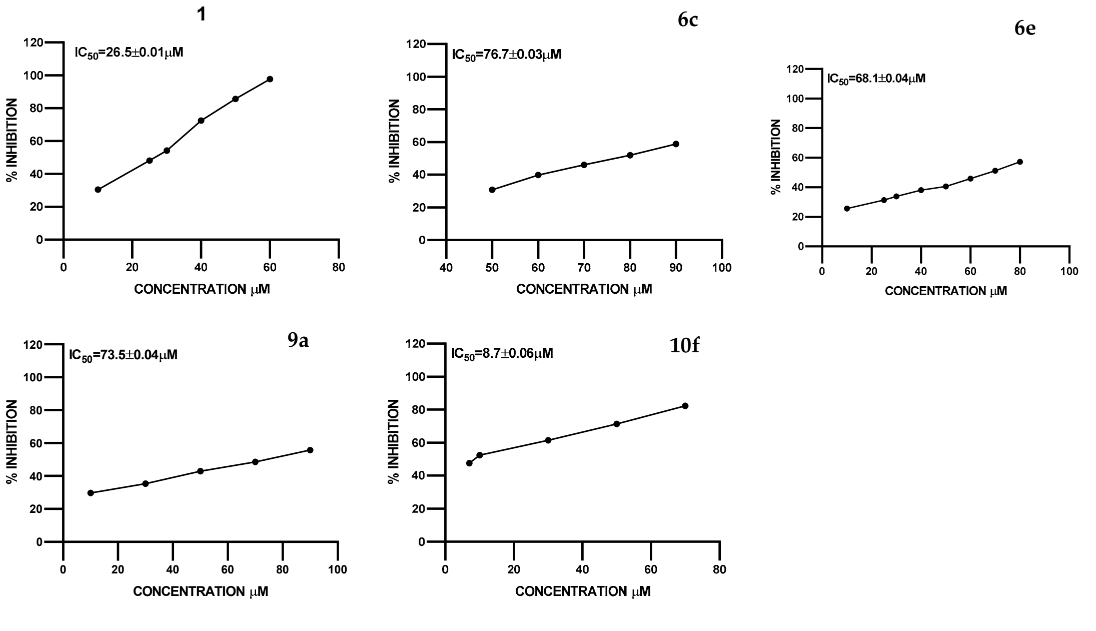

Figure S83. IC_{50} of compounds against α-amylase enzyme.

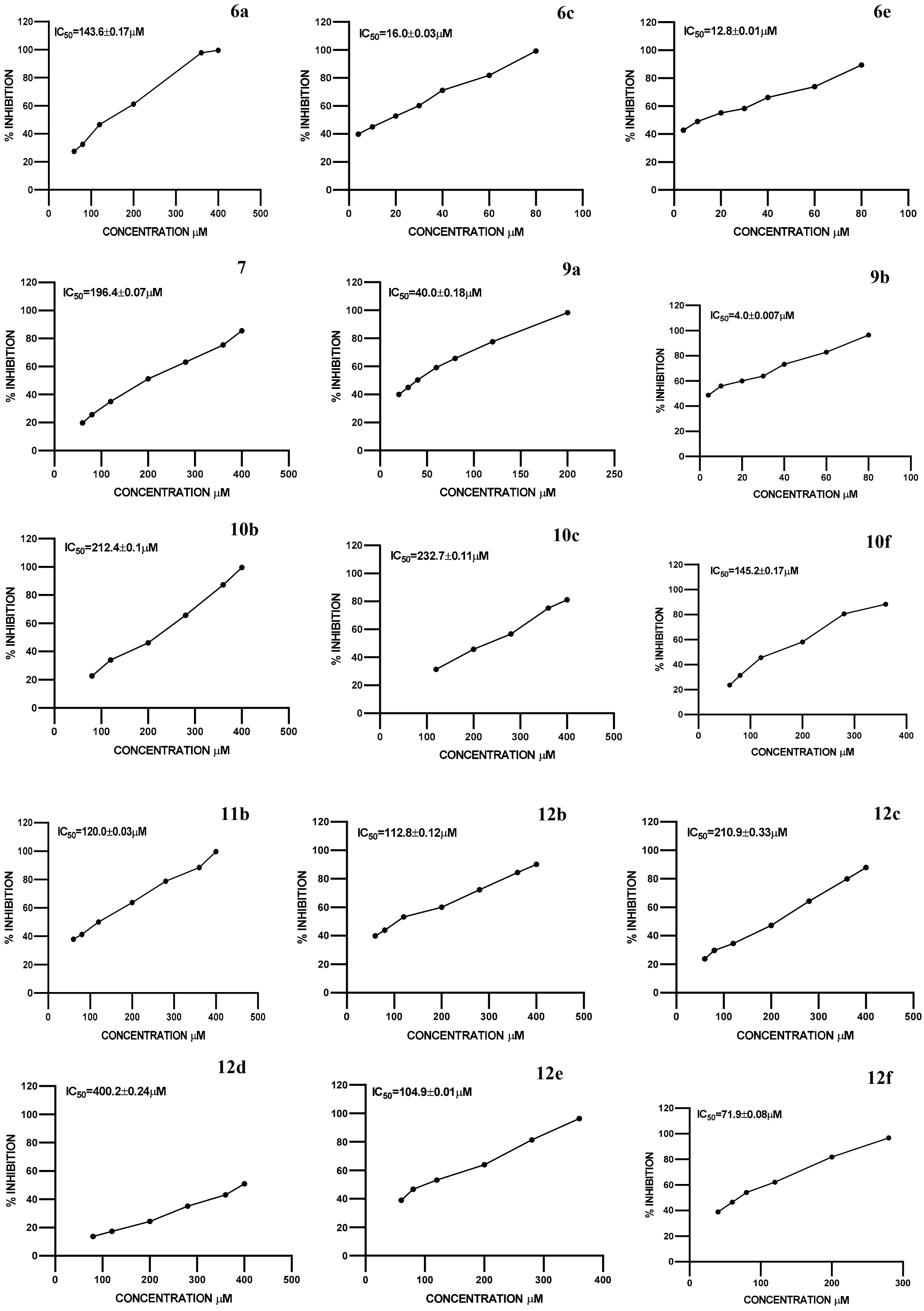

Figure S84. IC_{50} of compounds against α-glucosidase enzyme.
